# Spiral-Wave Turbulence and Its Control in the Presence of Inhomogeneities in Four Mathematical Models of Cardiac Tissue

**DOI:** 10.1371/journal.pone.0004738

**Published:** 2009-03-09

**Authors:** T. K. Shajahan, Alok Ranjan Nayak, Rahul Pandit

**Affiliations:** 1 Centre for Condensed Matter Theory, Department of Physics, Indian Institute of Science, Bangalore, India; 2 Indian Institute of Science Eduation and Research (IISER), Thiruvananthapuram, CET Campus, Thiruvananthapuram, Kerala, India; 3 Jawaharlal Nehru Centre for Advanced Scientific Research, Bangalore, India; University of Nottingham, United Kingdom

## Abstract

Regular electrical activation waves in cardiac tissue lead to the rhythmic contraction and expansion of the heart that ensures blood supply to the whole body. Irregularities in the propagation of these activation waves can result in cardiac arrhythmias, like ventricular tachycardia (VT) and ventricular fibrillation (VF), which are major causes of death in the industrialised world. Indeed there is growing consensus that spiral or scroll waves of electrical activation in cardiac tissue are associated with VT, whereas, when these waves break to yield spiral- or scroll-wave turbulence, VT develops into life-threatening VF: in the absence of medical intervention, this makes the heart incapable of pumping blood and a patient dies in roughly two-and-a-half minutes after the initiation of VF. Thus studies of spiral- and scroll-wave dynamics in cardiac tissue pose important challenges for in vivo and in vitro experimental studies and for in silico numerical studies of mathematical models for cardiac tissue. A major goal here is to develop low-amplitude defibrillation schemes for the elimination of VT and VF, especially in the presence of inhomogeneities that occur commonly in cardiac tissue. We present a detailed and systematic study of spiral- and scroll-wave turbulence and spatiotemporal chaos in four mathematical models for cardiac tissue, namely, the Panfilov, Luo-Rudy phase 1 (LRI), reduced Priebe-Beuckelmann (RPB) models, and the model of ten Tusscher, Noble, Noble, and Panfilov (TNNP). In particular, we use extensive numerical simulations to elucidate the interaction of spiral and scroll waves in these models with conduction and ionic inhomogeneities; we also examine the suppression of spiral- and scroll-wave turbulence by low-amplitude control pulses. Our central qualitative result is that, in all these models, the dynamics of such spiral waves depends very sensitively on such inhomogeneities. We also study two types of control schemes that have been suggested for the control of spiral turbulence, via low amplitude current pulses, in such mathematical models for cardiac tissue; our investigations here are designed to examine the efficacy of such control schemes in the presence of inhomogeneities. We find that a local pulsing scheme does not suppress spiral turbulence in the presence of inhomogeneities; but a scheme that uses control pulses on a spatially extended mesh is more successful in the elimination of spiral turbulence. We discuss the theoretical and experimental implications of our study that have a direct bearing on defibrillation, the control of life-threatening cardiac arrhythmias such as ventricular fibrillation.

## Introduction

Cardiac arrhythmias like ventricular tachycardia (VT) and ventricular fibrillation (VF) are a major cause of death in industrialised countries. Experimental studies over the past decade or so have suggested that VT is associated with an unbroken spiral wave of electrical activation on cardiac tissue but VF with broken spiral waves [1]–[3]. There is growing consensus [4], [5] that the analogs of VT and VF in mathematical models for cardiac tissue are, respectively, (a) a single rotating spiral wave in two dimensions or a scroll wave in three dimensions and (b) spiral-wave or scroll-wave turbulence [6]–[8]. It is imperative, therefore, to study spiral- and scroll-wave turbulence systematically in such mathematical models. There are several such studies [9]–[11] but none, to the best of our knowledge that compares several models with a view to elucidating low-amplitude defibrillation schemes, which are designed to eliminate spiral-wave turbulence, especially in the presence of inhomogeneities, such as conduction inhomogeneities in cardiac tissue. We address this question here by considering four models of cardiac tissue that are, in order of increasing complexity, (a) the Panfilov model [12], (b) the Luo-Rudy Phase I (LRI) model [13], (c) the reduced Priebe-Beuckelmann (RPB) model [14], and (d) the TNNP model of ten Tusscher, *et al*. [15].

The Panfilov model [12] is of the Fitzhugh-Nagumo [16], [17] type with two fields, namely, the transmembrane potential V and the recovery variable g, which depend on space and time; it is much simpler than models that account for ion channels in the membrane; nevertheless, it yields spiral waves and their break up in a manner that is qualitatively similar to such pattern formation in more realistic ionic models; given its simplicity, the Panfilov model is a good testing ground for numerical and semi-analytical studies. Realistic ionic models, such as the LRI, RPB, and TNNP models, are based on the Hodgkin-Huxley [18] formalism for ion channels. The LRI model [13] uses data from measurements on guinea-pig myocardial cells and accounts for ion channels and voltage dependent ion-channel gating variables. The RPB model [14] improves on the LRI model by incorporating data obtained from human ventricular muscles; furthermore, it includes an ion pump and an ion exchanger. The TNNP model is based on recent experimental data from human ventricular cells; it includes an ion pump, an ion exchanger, and more details of the calcium-ion dynamics [15] than the LRI and RPB models.

Our goal is to carry out a comparison of spiral-wave dynamics in these four models of cardiac tissue especially in the presence of different types of inhomogeneities, such as conduction and ionic inhomogeneities; a comparison of such spiral waves in the Priebe Beuckelmann (PB), RPB, TNNP, models and the model of Iyer *et al*. [19] has been presented in Ref. [20] but without inhomogeneities. We also study schemes for eliminating spiral-wave chaos from the simulation domain in Panfilov, LRI, RPB, and TNNP models in both homogeneous and inhomogeneous cases. The motivation for undertaking this study is that cardiac tissue contains both conduction and ionic heterogeneities. These can be caused, *inter alia*, by (a) a myocardial infarction that leads to ischemia [21], the subsequent damage or death of the affected cardiac cells, and, in the latter case, the formation of scar tissue that is non-conducting, (b) chronic heart failure, (c) genetic disorders, or (d) the presence of major blood vessels.

### Review of Previous Work

Conduction inhomogeneities in cardiac tissue can affect spiral waves in several ways. Experimental studies [22]–[24] have found that such inhomogeneities can anchor a spiral wave or, in some cases, can even eliminate it completely [2]. Studies of the dependence of such anchoring on the size of the obstacle [22]–[24] reveal that the larger the obstacle the more likely is the anchoring; however, even if the obstacle is large, the wave might not attach to it; furthermore, small obstacles can anchor spiral waves, albeit infrequently [24]. Such behaviors have also been seen in numerical simulations of spiral-wave turbulence in models for cardiac tissue: In particular, Xie *et al.* [25], have studied the dynamics of spiral waves in the LRI model in a two-dimensional (2D) circular domain with a circular hole in the middle: The parameters and initial condition are so chosen that, without the hole, spiral waves break up in the simulation domain. By shrinking the radius of the hole, the system is changed continuously from a 1D ring to 2D tissue with an obstacle, and, finally, to homogeneous 2D tissue [the hole radius is changed from that of the simulation domain (≃9.2 cm) to zero]. When the radius of the hole is very large, the system is effectively a 1D ring; the wave just goes around this ring. As the radius of the hole is decreased, the wave appears as a spiral anchored on the hole but rotating around it periodically, if the hole is large. As the hole radius is decreased a transition occurs first to a quasiperiodically rotating spiral wave and, eventually, to spiral-wave break up and spatiotemporal chaos [25] with the spirals not attached to the hole.

ten Tusscher *et al.* [26] have studied the Panfilov model with nonexcitable cells distributed randomly in it. In particular, they investigate spiral-wave dynamics as a function of the percentage of the simulation domain covered by such nonexcitable cells and find that, when this percentage is high, spiral-wave break up can be suppressed.

A detailed numerical and analytical study of the interaction of excitation waves with a piecewise linear obstacle has been carried out in Ref. [27]. This study finds that, if the excitability of the medium is high, the wave moves around the obstacle boundary, rejoins itself, and then proceeds as if it had not encountered any obstacles in its path. However, if the excitability is low, the two ends of this wavefront are unable to join, so two free ends survive, curl up, and then develop into two spiral waves, which can in turn break up again. In addition, apart from the excitability of the medium and the local curvature of the wave front, the shape of the obstacle also affects the attachment of spiral waves to it. We have carried out a detailed numerical study [28] of spiral-wave dynamics in the presence of conduction inhomogeneities in the Panfilov and LRI models; our study has shown that the dynamics of spiral waves depends very sensitively on the position of a conduction inhomogeneity.

### Summary of Our Results

Ionic inhomogeneities, formed say by local modifications of the maximal conductance of calcium ion channels, affect the action potential of a cardiac cell; in particular, the action potential duration (APD) and other time scales, such as the extent of the plateau region and the refractory period [29], are modified by these inhomogeneities and affect spiral wave dynamics in turn. For example, the stability of a spiral wave, in homogeneous, two-dimensional cardiac tissue depends on the maximal amplitude of the slow inward calcium current (governed by the conductance G_si_) as illustrated by the numerical study of Qu *et al.* [30] for the LRI model: As they increased G_si_ they first observed a rigidly rotating spiral wave, then one in which the spiral tip meandered quasiperiodically, and eventually chaotically; finally they obtained spiral turbulence with broken spiral waves. Furthermore, the numerical studies of Refs. [28], [31] have found that ionic heterogeneities can play an important role in the initiation and break up of spiral waves; and Ref. [28] has presented preliminary studies of the Panfilov-model analog of ionic inhomogeneities.

We consider spiral-wave dynamics in an otherwise homogeneous medium with a square region in which the conduction or ionic parameters are different from their values in the rest of the simulation domain. We find that such inhomogeneities can have dramatic effects on spiral wave dynamics. We have reported earlier that conduction inhomogeneities can act as anchoring sites for spirals, or lead to the complete elimination of spiral waves, or have no effect on spiral-wave break up; which one of these results is obtained depends on the size and position of the conduction inhomogeneity [28]. In this paper we extend our work to ionic inhomogeneities. We find that such inhomogeneities can also result in the elimination of spiral waves; this depends on the position of the inhomogeneity. Here too we find anchored spirals, but with richer dynamics than with conduction inhomogeneities; e.g., we find states with rotating spiral waves that show period-4 and period-5 cycles and also states that show a coexistence of a periodically rotating spiral-wave and chaotic patterns with broken spiral waves, in the region of the ionic inhomogeneity. Lastly we investigate the efficacy of two low-amplitude schemes [32], [33] that have been suggested for the control of spiral-wave turbulence in mathematical models for cardiac tissue. In particular, we carry out detailed simulations of such control schemes in the presence of conduction inhomogeneities; our study shows that the elimination of spiral-wave turbulence is considerably more complicated if inhomogeneities are present than if they are not.

This paper is organised as follows: In the Section on “Methods” we present the models and numerical methods that we use in our study. In the Section on “Results” we present our results on studies of spiral-wave dynamics in the presence of conduction and ionic inhomogeneities; we then give an analysis of two different low-amplitude control schemes for the elimination of spiral-wave turbulence in models for cardiac tissue; finally we examine the efficacies of these control schemes in the presence of conduction inhomogeneities. The concluding Section “Discussion” contains a summary of the significance of our results. The Supplementary Material S1 contains equations for the ionic models we study and additional figures that give more details about our results.

## Methods

The Panfilov model [12], [34] comprises two coupled partial differential equations (PDEs) for the transmembrane potential V (denoted by e in Refs. [12], [34]) and the slow, recovery variable g, via which this model accounts, approximately, for the effects of the different ion channels in cardiac tissue; both V and g depend on the spatial coordinate **x** and time t:
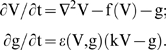
(1)The fast initiation of the action potential of this model is encoded in the following piecewise linear form of f(V): f(V) = C_1_V, for V<V_1_, f(V) = −C_2_V+a, for V_1_≤V≤V_2_, and f(V) = C_3_(V−1), for V>V_2_. The physically appropriate parameters are [12], [34] V_1_ = 0.0026, V_2_ = 0.837, C_1_ = 20, C_2_ = 3, C_3_ = 15, a = 0.06 and k = 3. The time scales of the recovery variable are determined by the function ε(V,g) that is ε_1_ for V<V_2_, ε_2_ for V>V_2_, and ε_3_ for V<V_1_ and g<g_1_; g_1_ = 1.8, ε_1_ = 0.01, ε_2_ = 1.0, and ε_3_ = 0.3; here we deal with dimensionless quantities. To obtain dimensioned quantities [12], [34] we define dimensioned time to be 5 ms times dimensionless time, 1 spatial unit to be 1 mm, and the dimensioned value of the conductivity constant D to be 2 cm^2^/s. In some of our studies we vary ε_1_ to mimic the effects of ionic inhomogeneties. Voltages in this model are scaled such that the resting potential is zero.

Even though the Panfilov model is very simple when compared to the LRI, RPB, and TNNP models, which retain details of ion channels, it captures several essential properties of the spatiotemporal evolution of V [12], [34]–[36]. In particular, the action potential of the Panfilov model contains both the *absolute* and *relative refractory* periods seen in more advanced models. Furthermore, the appearance, propagation, and break up of spiral-wave patterns and the ways in which they can be controlled are similar in these models as we will illustrate here.

The LRI model uses the Hodgkin-Huxley formalism for ion channels in a given cell. It accounts for 6 ionic currents (e.g., Na^+^, K^+^, and Ca^2+^) that flow through voltage-gated ion channels; 9 gate variables regulate the transport of ions across the cell membrane [13]; in the quiescent state the concentration differences of the ions, inside and outside the cell, is such that a potential difference ≃−84 mV is induced across this membrane. Electrical stimuli, which raise the potential across the cell membrane above ≃−60 mV, change the conductances of the ion channels and thus yield an action potential with a typical duration of ≃200 ms. After the initiation of the action potential, there is a refractory period during which a stimulus of the same strength cannot lead to further excitation of that cell. This excitation moves from one cell to another in the LRI model by virtue of a diffusive coupling; thus the transmembrane potential V obeys a reaction-diffusion-type PDE that includes ionic currents (see Section 2 of the Supplementary Material S1 for details); the time evolution and V dependence of these currents are given by 7 coupled ordinary differential equations (ODEs) [13], [28].

We also study the Reduced-Priebe-Beuckelmann (RPB) model [14] that is a simplified version of the Priebe-Beuckelmann (PB) model [37]. The original PB model is a second-generation model based on the phase-2 Luo-Rudy model [38] of a guinea-pig ventricular myocyte with currents scaled to fit human-cell data. In the PB model ion concentrations in a cell can vary in time; and it accounts for some ion pumps. Such second-generation models reproduce ionic currents and concentrations in a single cell during electrical activity; however, the large number of variables in these models pose a significant computational challenge, especially in the simulation of arrhythmias in three-dimensional (3D) and even in two-dimensional (2D) domains. This has motivated the development of the reduced PB (RPB) model, in which the variables are reduced from 15 to 6 by a reformulation of some currents and by approximating all intracellular ionic concentrations by suitable constants. Nevertheless, the RPB model retains important properties of human ventricular tissue such as restitution properties, the shape of the action potential (AP), and the change of this shape as a function of major ionic currents. As in the LRI model, cells in the RPB model have a diffusive coupling with each other; the equations for the RPB model are given in Section 2 of the Supplementary Material S1. In particular, the equilibrium voltage across the cardiac cell membrane is −91 mV in the RPB model.

The most realistic model we study is the one introduced recently [15] by ten Tusscher, Noble, Noble, and Panfilov (TNNP). It is based on experimental data obtained from human ventricular cells. The TNNP model allows for variations of intracellular ion concentration, as in other second-generation models, contains 12 ionic currents, 12 gating variables, one ion pump, and an ion exchanger. All major ionic currents are included in the TNNP model, e.g., the fast inward Na^+^ current I_Na_, the L-type Ca^2+^ current I_CaL_, the transient outward potassium current I_to_, the slow, potassium, delayed, rectifier current I_Ks_, the rapid, potassium, delayed, rectifier current I_Kr_, and the inward, rectifier K^+^ current I_K1_. These and other currents and the details of the dynamics of calcium ions are given in Section 2 of the Supplementary Material S1. As in the LRI and RPB models, cells in the TNNP model are coupled diffusively.

Since this model has many variables, numerical simulations of spiral-wave dynamics in it are considerably harder than in the simpler LRI and RPB models. In both the RPB and TNNP models, we can study human epicardial, endocardial, and M cells by a suitable choice of parameters; for the RPB model we use the parameters for M cells and for the TNNP model we use parameters for epicardial cells (the equilibrium voltage across the cardiac cell in the latter is −86.2 mV).

To integrate the Panfilov model PDEs in d spatial dimensions we use the forward-Euler method in time t, with a time step δt = 0.022, and a finite-difference method in space, with step size δx = 0.5, and five-point and seven-point stencils, respectively, for the Laplacian in d = 2 and d = 3 for spatial grids on square or simple-cubic simulation domains with side L mm, i.e., (2L)^d^ grid points. We use a similar forward-Euler method for the LRI PDEs, with δt = 0.01 ms, and a finite-difference method in space, with δx = 0.0225 cm, and a square simulation domain with side L = 90 mm, i.e., 400×400 grid points. We have checked in representative simulations for the LRI model that a Crank Nicholson scheme yields results in agreement with the numerical scheme described above. The simulation schemes that we use for the RPB and TNNP models are similar to the one we use for the LRI model; for the RPB model we use δt = 0.01 ms, δx = 0.0225 cm, and a 512×512 square simulation domain; for the TNNP case we use δt = 0.02 ms, δx = 0.0225 cm, and a 600×600 square simulation domain (i.e., L = 135 mm).

For all the models that we study, we use Neumann (no-flux) boundary conditions. The initial conditions we use will be specified below. For numerical efficiency, these simulations have been carried out on parallel computers with MPI codes that we have developed for all these models.

As suggested in Ref. [39], it is useful to test the accuracy of the numerical scheme used by varying both the time and space steps of integration. We illustrate this for the TNNP model by measuring the conduction velocity (CV) of a plane wave, which is injected into the medium by stimulating the left boundary of our simulation domain. We find that, with δx = 0.0225 cm CV increses by 1.3% as we decrease δt from 0.02 to 0.01 ms; if we use δt = 0.02 ms and decrease δx from 0.0225 to 0.015 cm then CV increases by 3.3%; such changes are comparable to those found in earlier studies [15], [39].

We introduce conduction inhomogeneities, which we also refer to as obstacles, in the simulation domains of the models described above by making the conductivity constant D = 0 in the region of the obstacle. In most of our studies we use square and square-prism obstacles in two and three dimensions, respectively. When we set D = 0 we decouple the cells inside the obstacle from those outside it. Furthermore, we use Neumann (i.e., no-flux) boundary conditions on the boundaries of the obstacle; we have checked in representative cases that, even if we do not impose Neumann boundary conditions on the obstacle boundaries, our results are not changed qualitatively.

We insert ionic inhomogeneities into our simulation domains by changing the values of the maximal conductances of Ca^2+^ channels, in the region of the inhomogeneity, for the LRI, RPB, and TNNP models. Since the Panfilov model does not account explicitly for Ca^2+^ ion channels, we mimic ionic inhomogeneities here by altering the value of the parameter ε_1_ in the region of the ionic inhomogeneity. In most of our studies we use square ionic inhomogeneities in two dimensions.

## Results

All the models described above can support spiral waves in a homogeneous simulation domain if we use suitable initial conditions. We begin the description of our results with an overview of such homogeneous simulations for the TNNP model; our work here complements that of Ref. [20]. Spiral waves in homogeneous simulation domains in Panfilov, LRI and RPB models are discussed in Section 3 of the Supplementary Material S1. We then extend our study to simulations with either (a) conduction inhomogeneities or (b) ionic inhomogeneities. This is followed by a discussion of our results on some schemes for the suppression of spiral-wave turbulence in these models; these suppression schemes are the numerical analogs of defibrillation. We consider the efficacy of a few defibrillation schemes in both homogeneous domains and in the presence of the conduction and ionic inhomogeneities described above.

### Spiral waves in homogeneous domains

Given the diffusive coupling between cardiac cells, an action potential, generated in one cell, can excite neighboring cells and thus spread out as an expanding wave. However, since the initial excitation is followed by a refractory period, a second wave cannot follow the first one immediately. Each such wave leaves in its wake a nonexcitable region, so the next wave can follow it only at a distance determined by the product of the refractory period and the wave-conduction velocity [40], [41]. When two plane waves collide in such a medium, they cannot pass through each other because they have non-excitable wakes. Hence a collision leads to the annihilation of these colliding waves.

Furthermore, the conduction velocities of these waves depend on the curvature of the wavefront [42]: A concave wave moves faster than a rectilinear wave, which in turn travels faster than a convex wave. Any deviations from a planar wave front are, therefore, amplified or attenuated depending on the curvature of the deviation; and they eventually lead to the formation of rotating spiral waves. Above a critical curvature of the wavefront, the current flux from the wavefront is not sufficient to excite the medium around it. This failure of wave conduction then leads to a break up of the wave. These wave fragments move around in the domain, regenerate themselves by using excitable regions, avoid regions that are still in a refractory state, and the parts of these fragments that collide annihilate one another. In a sufficiently large excitable medium this activity of wave fragments can lead to complex spatiotemporal dynamics. The resulting spiral-turbulence state, an instance of spatiotemporal chaos, is characterised by many positive Lyapunov exponents [32].

We show below how such spiral waves can be generated, and how they break up subsequently, in representative, two-dimensional simulations for the TNNP model in homogeneous domains. (Similar simulations for Panfilov, LRI and RPB models are given in Section 3 of the Supplementary Material S1.) Initial conditions have to be chosen carefully to obtain spiral waves; we describe these below. And we use these initial conditions in subsequent Sections that are devoted to our studies of the interactions of such spiral waves with inhomogeneities.

#### Spiral waves in the TNNP model

**Figure 1:**
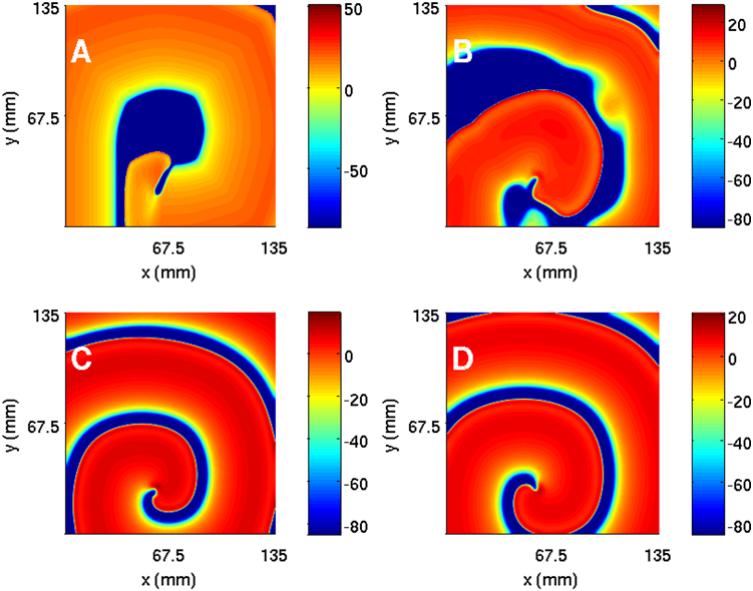
(Color online) The TNNP model in a square simulation domain of side L = 135 mm. Pseudocolor plots of the transmembrane potential V at (A) t = 0 s; (B) t = 0.8 s; (C) t = 3.2 s; and (D) t = 4.8 s.

**Figure 2:**
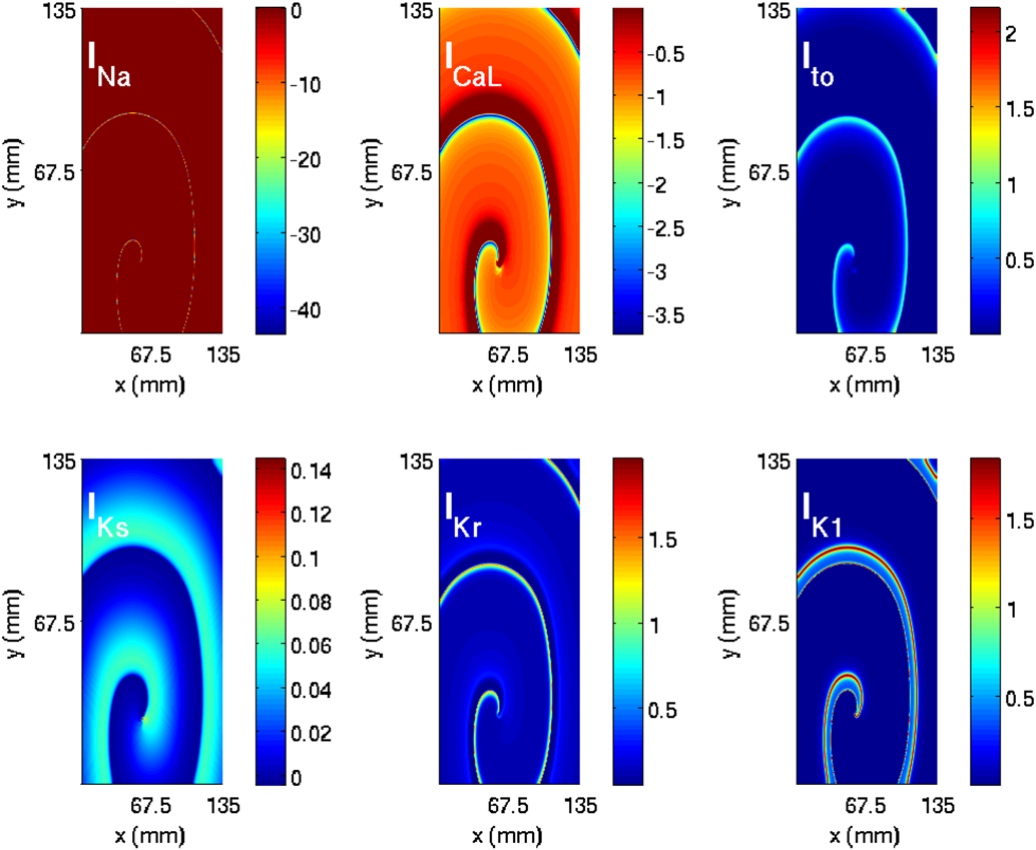
(Color online) A comparison of pseudocolor plots of ionic currents in the TNNP model to the transmembrane potential V at t = 4.8 s (cf. the pseudocolor plot of Fig. 1D) in a square simulation domain of side L = 135 mm. (A) fast Na^+^ current (I_Na_); (B) L-type Ca^2+^ current (I_CaL_); (C) transient outward current (I_to_); (D) slow delayed rectifier current (I_Ks_); (E) rapid delayed rectifier current (I_Kr_); (F) inward rectifier current (I_K1_).

We obtain spiral waves in the TNNP model by injecting a plane wave into the domain via a stimulation current of 150 µA/cm^2^ for 2 ms at the left boundary. As this plane wave moves towards the right boundary and 270 ms after the first stimulus, we apply a second stimulus of 450 µA/cm^2^ along a line behind this wave but parallel to it [14], [15] (x = 290, 1≤y≤250) for 10 ms. As the first wave moves further towards the right, the free end of the new stimulus is able to move into the area behind the first wave; a hook-like proto spiral appears at this free end. We now change the conductivity D from 0.00154 to 0.000385 cm^2^/ms between 304 ms to 524 ms; this yields the fully developed spiral wave. We then reset the conductivity to its original value after 524 ms. At the moment the first plane wave is initiated the currents and gating variables are initialised as follows: the gating variables are given in Supplementary Material S1 and the currents are calculated for these values of the gating variables and the resting value of V which is −86.2 mV. We show the initiation of a spiral wave in the TNNP model in Section 3D of the Supplementary Material S1. The procedure described above results in the spiral wave that is shown via the sequence of pseudocolor plots for the transmembrane potential V (Fig. 1) and the currents I_Na_, I_CaL_, I_to_, I_Ks_, I_Kr_, I_K1_, I_NaCa_, I_NaK_, I_pCa_, I_pK_, I_bNa_, and I_bCa_ (Fig. 2); the states shown in these figures are used as initial conditions for our subsequent simulations of the TNNP model with and without inhomogeneities. In the absence of inhomogeneities such an initial condition leads to a spiral wave as shown in the illustrative pseudocolor plot of V in Fig. 1.

#### A comparison of spiral waves in different models

**Figure 3:**
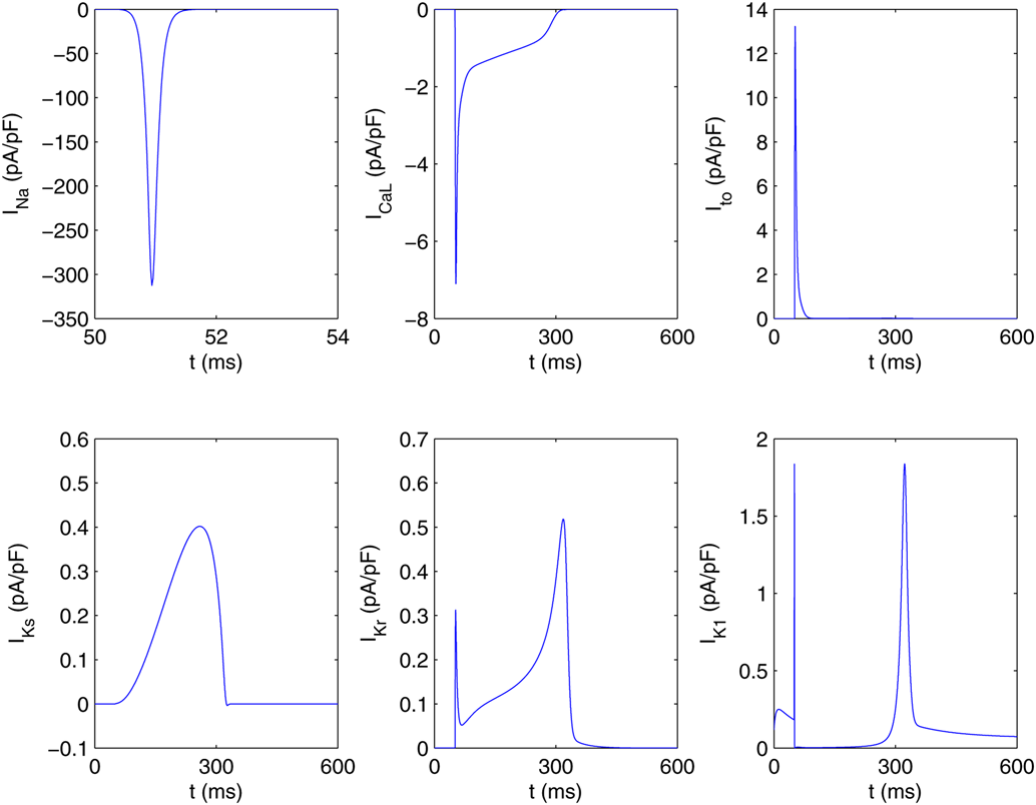
Plots showing the dependence of the currents I_Na_, I_CaL_, I_to_, I_Ks_, I_Kr_, and I_K1_ on time t during the course of the action potential from our single-cell simulation of the TNNP model. Negative currents move into the cell and positive currents move out of the cell.

From our studies of spiral waves in the four models described above, we see that many qualitative features of spiral-wave dynamics are the same in the Panfilov, LRI, RPB, and TNNP models. However, there are important differences, some qualitative and the others quantitative. For example, the Panfilov model cannot address directly any questions regarding currents in ion channels since it does not follow their evolution but only considers one slow recovery variable g. The LRI, RPB, and TNNP models do give spatiotemporal information for several ion-channel currents as shown, for a representative case, in Fig. 2. At any given time, the qualitative form of the spatial organization of these currents can be surmised from the spatial distribution of the transmembrane potential V and the dependence of these currents on V at the level of a single cell. To illustrate this we show for the TNNP model, in Fig. 3, the temporal evolution of the currents from a single-cell simulation (i.e., without the diffusion term in the TNNP equations). For instance, at the single-cell level, the sodium current I_Na_ is substantial only at the beginning of the action potential (Fig. 3); the most prominent parts of the spiral waves in pseudocolor plots of V appear in regions of the simulation domain where, locally, V assumes a value close to the sharp peak in the single-cell action potential; thus pseudocolor plots of the sodium current I_Na_ (Fig. 2) show significant structure only in narrow strips that follow closely the prominent parts of the spiral waves in pseudocolor plots of V (Fig. 2). By contrast, the calcium current I_CaL_ is significant in the plateau regime of the action potential (Fig. 3); thus pseudocolor plots of I_CaL_ (Fig. 2) show structure in most parts of the simulation domain, but the underlying spiral wave in V is still discernible. The potassium current I_K1_ is substantial in the repolarization regime of the action potential (Fig. 3), so we should expect pseudocolor plots of I_K1_ to have significant structure along the back of this wave, where repolarization occurs; this expectation is borne out as can be seen from Fig. 2. Similar considerations can be used to rationalize, qualitatively, the remaining pseudocolor plots for other currents in the LRI, RPB, and TNNP models; representative plots are given in the Supplementary Material S1.

**Figure 4:**
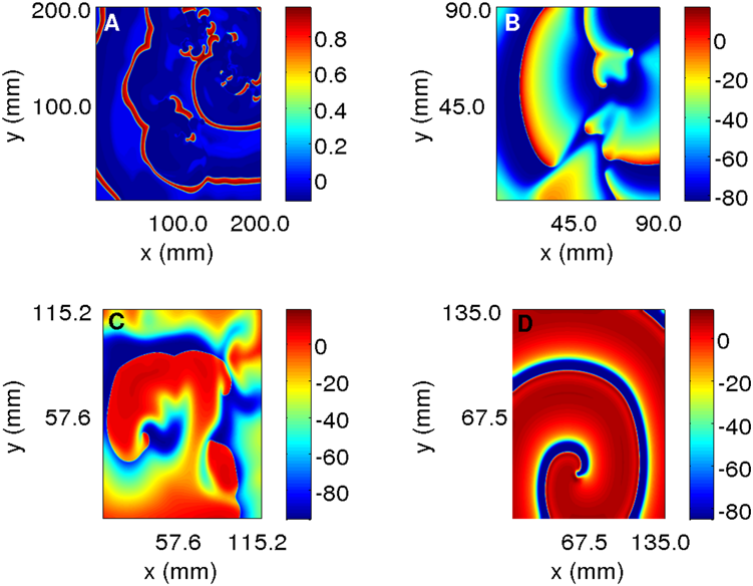
Pseudocolor plots of transmembrane potential V from two-dimensional simulations of (A) Panfilov, (B) LRI, (C) RPB, and (D) TNNP models.

**Figure 5:**
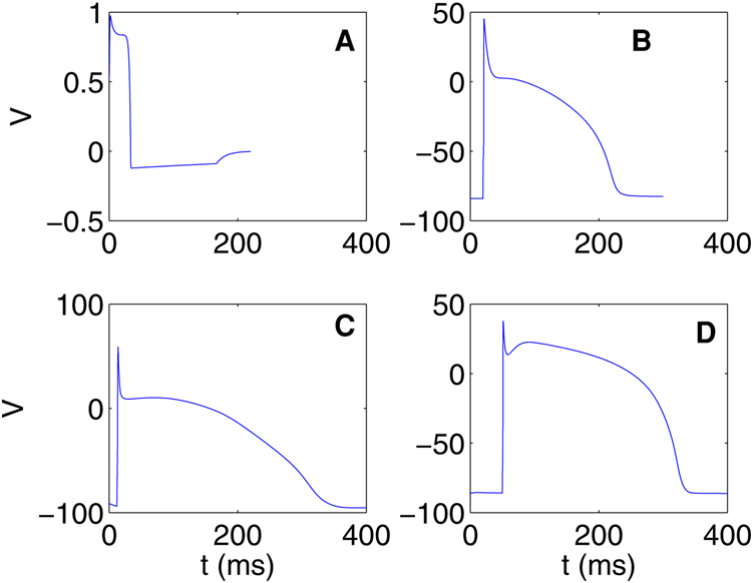
Action potentials from single-cell simulations of the (A) Panfilov, (B) LRI, (C) RPB, (D) TNNP models. Note that the action potentials from the Panfilov and TNNP models fall off more sharply in the repolarization phase than those for the LRI and RPB models.

It is useful to contrast pseudocolor plots of V for the Panfilov, LRI, RPB, and TNNP models. The qualitative features of these plots are the same but they differ in detail [43]. Such differences arises from the differences in the single-cell dynamics of these models, e.g., the action-potential duration, the refractory period, the shape of the repolarization part of the action potential, etc. From the representatives pseudocolor plots of the four models (Fig. 4), we see that spiral waves in the Panfilov and TNNP models appear more sharp in these plots than their counterparts in the LRI and RPB models. This can be understood qualitatively by comparing the single-cell action potentials for these four models. Figure 5 gives such a comparison, which shows clearly that the repolarization in the Panfilov and TNNP models is sharper and more rapid than in the LRI and RPB models. The sharpness of the spiral waves in the former two models and their relatively diffuse character in the latter two models is related to these differences in repolarization.

### Conduction inhomogeneities

We have elucidated the effects of conduction inhomogeneities in the Panfilov and LRI models in Ref. [28]. We begin with a brief recapitulation of these results and then present new ones for the RPB and TNNP models with obstacles. Extensions to the case of two conduction inhomogeneities and their interactions with spiral waves are discussed elsewhere [44], [45].

We first examine the dependence of spiral-wave dynamics on the size of an obstacle by fixing its position and changing its size (cf., Ikeda *et al.* [22] for similar experiments): Specifically we place a square obstacle of side l in the Panfilov model in a square simulation domain with side L = 200 mm and use parameter values that yield spiral turbulence (ST) in the absence of the obstacle. With the bottom-left corner of the obstacle at (50 mm, 100 mm) ST persists if l≤(40−Δ) mm; it gives way to a quiescent state (Q) with no spirals if l = 40 mm, and then to a state with a single rotating spiral (RS) anchored at the obstacle if l>(40+Δ) mm; here we vary l from 2 mm to 80 mm in steps of Δ = 1 mm. Thus, as l increases, we see a clear transition from ST to RS, with these two states separated by a state Q with no spirals. Henceforth, when we specify the position (x,y) of a square inhomogeneity, we will mean that the bottom-left corner of this inhomogeneity is placed at the point (x,y).

Furthermore, we find that the final state of the system depends on where the obstacle is placed with respect to the tip of the initial spiral wavefront. Even a small obstacle placed near this tip [e.g., an obstacle with l = 10 mm at (100 mm, 100 mm)] can prevent the spiral from breaking up; but a bigger obstacle, placed far away from the tip [e.g., an obstacle with l = 75 mm at (125 mm, 50 mm)], does not affect the spiral. In Ref. [28] we have explored in detail, for the Panfilov and LRI models, how the final state of the system depends sensitively on the position of the obstacle. We have found, in particular, that, if the spiral wave breaks up and yields a spatiotemporally chaotic state in the absence of any obstacles in the medium, then the introduction of an obstacle can lead to one of the following three outcomes: (a) spiral turbulence (ST) can persist; (b) ST can be replaced by a single rotating spiral wave (RS) anchored to the obstacle; (c) ST can give way to a quiescent state (Q) that occurs when all spiral waves move towards and are absorbed by the boundaries.

We show here that this sensitive dependence of spiral-wave dynamics on the position of an obstacle also occurs in the TNNP models. (Similar results for the RPB model are given in Section 4 of the Supplementary Material S1.)

**Figure 6:**
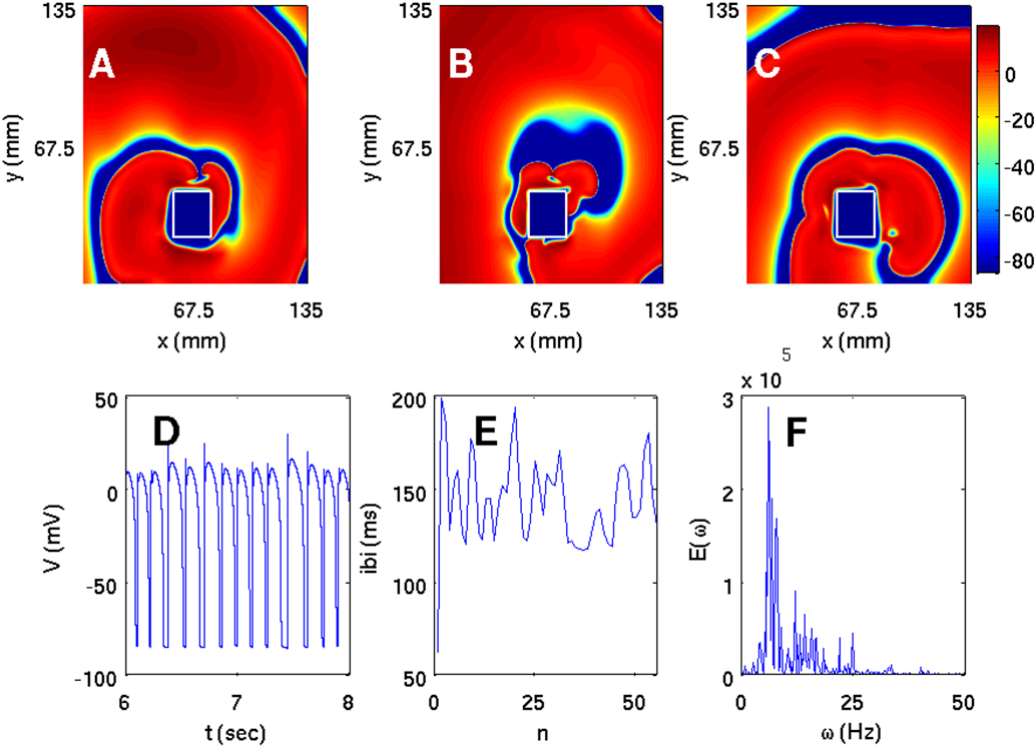
(Color online) The spiral-turbulence (ST) state in the TNNP model with a square obstacle of side l = 22.5 mm at (54 mm, 22.5 mm) in a square simulation domain with L = 135 mm. We start with the initial condition of Fig. 1(A); pseudocolor plots of V are shown in (A), (B), and (C) at 0.8, 2.4, and 4.8 s, respectively. (D) The local time series for V from a sample of 100000 iterations taken from the representative point (90 mm, 90 mm) after the removal of initial transients (the first 300000 iterations); and plots of (E) the inter-beat interval (IBI) versus the beat number n from a sample time series of 400000 iterations, and (F) the power spectrum of V obtained from a time series of length 200000 iterations (after the removal of initial transients in the first 200000 iterations); the non-periodic behavior of the IBI and the broad-band nature of the power spectrum are characteristic of the spiral-turbulence state.

**Figure 7:**
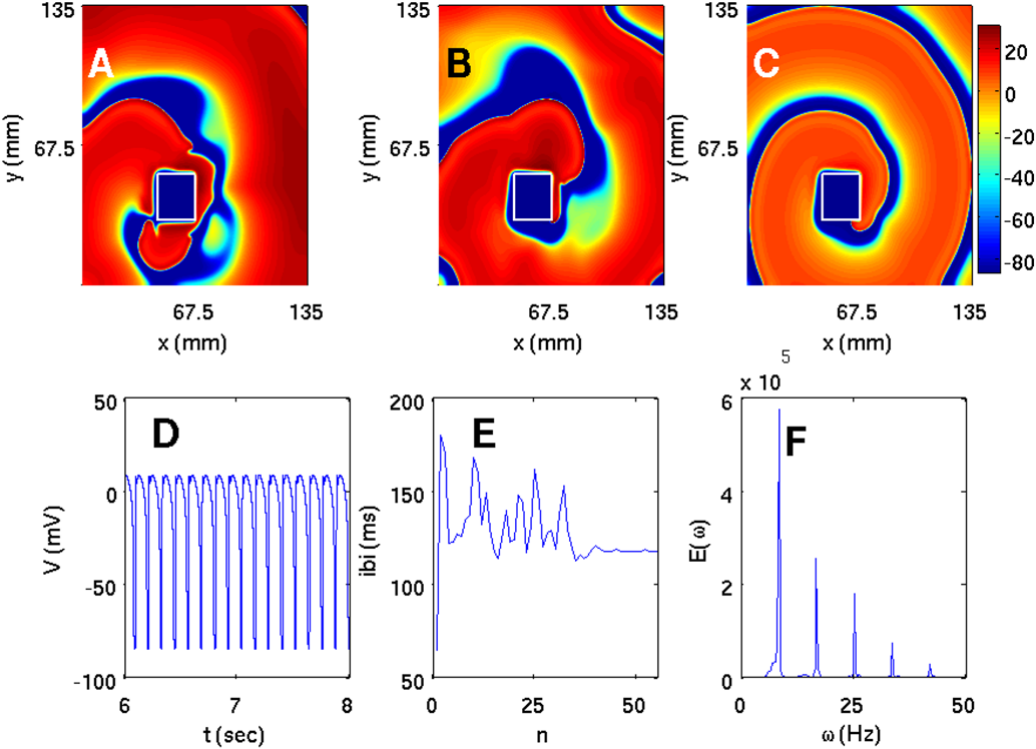
(Color online) Pseudocolor plots of V for the TNNP model showing a spiral wave attached to a square obstacle of side 22.5 mm placed at (45 mm, 31.5 mm) and (A) t = 1.6 s, (B) t = 4 s, and (C) t = 6.4 s. This wave leads to periodic temporal evolution as can be seen from plots of (D) the time series of V from a sample of 100000 iterations (after the removal of the first 300000 iterations) taken from the representative point (90 mm, 90 mm) in the square simulation domain of side L = 135 mm, (E) the IBI versus the beat number n (a sample of 400000 iterations) that settles, eventually, to a constant value of ≃118 ms, and (F) the power spectrum of V (from a time series of 200000 iterations after removal of the initial 200000 iterations) that has discrete peaks at the fundamental frequency ω_f_≃8.5 Hz and its harmonics.

**Figure 8:**
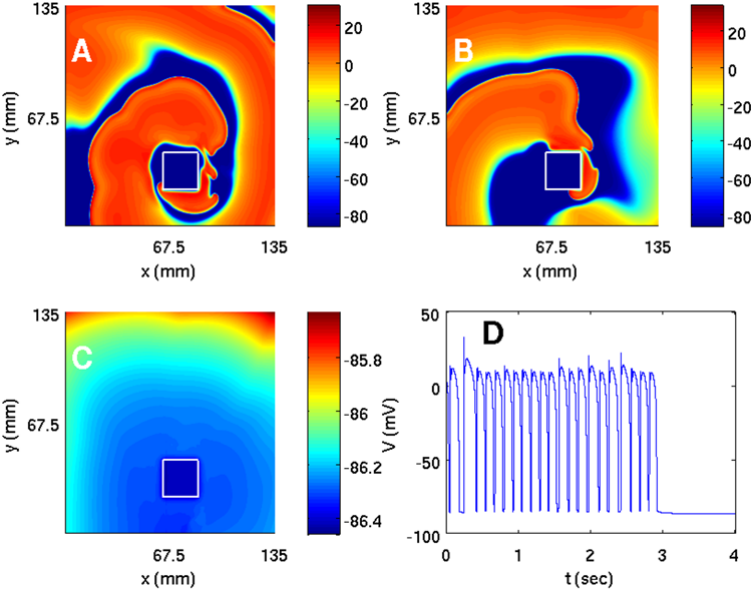
(Color online) The spiral wave moves away from the square simulation domain of side L = 135 mm for the TNNP model if a square obstacle of side l = 22.5 mm is placed at (63 mm, 22.5 mm) as illustrated in (A), (B), and (C) via pseudocolor plots of V at t = 0.8 s, t = 2 s, and t = 3.2 s, respectively. (D) The time series of V from a sample of 200000 iterations recorded from the representative point (90 mm, 90 mm); after t = 3.2 s the system is quiescent and V is −86.2 mV.

We have carried out a systematic study of spiral-wave dynamics in the TNNP model in the presence of an obstacle with the initial conditions specified above, namely, one spiral wave [Figs. 1(A)–(D)] that would continue rotating if the obstacle were not present. In the presence of an obstacle this rotating-spiral (RS) state can be replaced by one of the following possibilities: (a) the spiral wave can continue to rotate, without being anchored to the obstacle, and eventually break down to yield the state ST with spiral turbulence as shown, e.g., in Fig. 6; (b) the tip of the spiral wave can get anchored to the obstacle to give the state RS in which the anchored spiral rotates around the obstacle as shown, e.g., in Fig. 7; (c) all spiral waves can be absorbed by the boundaries so that the system evolves into the quiescent state Q as shown, e.g., in Fig. 8.

Specifically, if we use a square obstacle of side l = 22.5 mm at (54 mm, 22.5 mm), the system is in the state ST as shown in Figs. 6(A)–(C). In this state the time series of the transmembrane potential V (x,y,t), taken from the representative point (90 mm, 90 mm) and shown in Fig. 6(D), clearly displays nonperiodic, chaotic behavior. The time between successive spikes in such a time series, i.e., the interbeat interval (IBI), is plotted versus the integers n, which label successive spikes, in Fig. 6(E); this also shows the chaotic nature of the state ST. Figure 6(F) shows the power spectrum E(ω) of the time series depicted in Fig. 6(D); the broad-band nature of this power spectrum provides additional evidence for the chaotic character of ST. The chaotic nature of such time series and the inhomogeneous pattern of waves in pseudocolor plots of V in the state ST indicate that we have spatiotemporal chaos; however, since the TNNP model is far more complicated than the Panfilov model, it is not very easy to calculate the spectrum of Lyapunov exponents and thence the Kaplan-Yorke dimension for ionic models like the TNNP model.

If we change the position of the obstacle and place it at (45 mm, 31.5 mm), the spiral gets attached to it. For this case the analogs of Figs. 6(A)–(C) are shown in Figs. 7(A)–(C), respectively. From the time series of Fig. 7(D) we see that the transmembrane potential displays some initial transients but then settles into periodic behavior. This is also manifested in the plot of the IBI versus n in Fig. 7(E) in which the IBI approaches a constant value [118 ms in Fig. 7(E)]. The corresponding power spectrum in Fig. 7(F) consists of discrete peaks at frequencies ω = mω_f_, where m is a positive integer and ω_f_ is the fundamental frequency of spiral rotation (ω_f_≃8.5 Hz here).

We now place the obstacle at (63 mm, 22.5 mm); in this case the spirals get eliminated completely and we get the quiescent state shown via pseudocolor plots of V in Figs. 8(A)-4(C). The time series of V (x,y,t), taken from (x = 90 mm, y = 90 mm) and depicted in Fig. 8(D), clearly shows that we obtain a quiescent state Q with no spirals. Plots of the IBI and the power spectrum are not shown since V just goes to zero after an initial period of transients. The durations for which the transients last, say in Fig. 8(D), vary greatly depending on the position of the obstacle relative to the spiral tip. The sensitive dependence of spiral-wave dynamics on the position of an inhomogeneity is also obtained if we use obstacles that do not have a square shape. We show this explicitly for a circular obstacle in the Supplementary Material S1 and for obstacles of other shapes in Ref. [45].

This sensitive dependence of the dynamics of spiral waves on the position of an obstacle has been investigated systematically, especially for the Panfilov model, in our earlier work [28]. We have, in particular, obtained a stability diagram for this model as follows: We divide the simulation domain into small squares of side l_p_ = 10 mm. We then carry out a sequence of simulations. In each one of these simulations the square obstacle (slightly larger than the squares into which the simulation domain has been divided) is placed so that its bottom-left corner coincides with the bottom-left corner of one of the small squares; we now study the dynamics of spiral waves, with the initial condition described above, and determine whether the system reaches the ST, RS, or Q state. Our stability diagram, given in Ref. [28], depicts the simulation domain covered with the small squares mentioned above. The color of each small square indicates the final state of the system when the position of the bottom-left corner of the obstacle coincides with that of the small square: red indicates spiral turbulence (ST), blue a rotating spiral (RS) anchored at the obstacle, and green a quiescent state (Q) with no spirals. This stability diagram shows that RS occurs typically when the obstacle lies near the middle of the simulation domain whereas ST occurs when the obstacle lies near the boundary of the simulation domain; regions of Q occur in a few places along the boundary between regions of ST and RS. We have found that this boundary is very complicated; by zooming in on it, we have provided good numerical evidence that suggests that this boundary has a fractal-type character, which leads to the sensitive dependence of the final state of the system on the position of the obstacle. Even if we change the position of the obstacle slightly (say by ≃0.5 mm), the final state of the system can change from ST to RS or Q. We have suggested [28] that this fractal-type boundary between the ST and RS regions in our stability diagram is a manifestation of an underlying fractal basin boundary between the domains of attraction of the ST, RS, and Q states in the phase space of the infinite-dimensional dynamical system, i.e., the Panfilov-model partial differential equations; such a basin boundary is not easy to determine for an infinite-dimensional system but its signatures can be found in the sort of sensitive dependence on parameters, such as the position of the inhomogeneity, that we have elucidated above.

**Figure 9:**
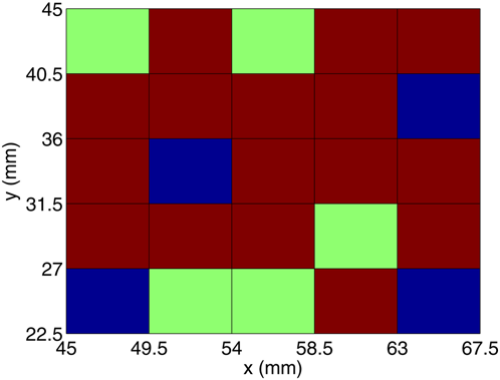
(Color online) Detail of the stability diagram for the TNNP model for G_CaL_ = 0.000044 and all other parameters as in 2 C of the Supplementary Material S1 and with the initial condition described in the text: A square obstacle of side 27 mm is placed at different positions in a square simulation domain with side L = 135 mm. For each one of these positions of the obstacle we determine the final state of the system; the colors of the small squares, of side l_p_ = 4.5 mm, indicate the final state of the system when the position of the bottom-left corner of the obstacle coincides with that of the small square (red, blue, and green denote ST, RS, and Q, respectively).

Similar stability diagrams can be obtained, in principle, for LRI, RPB, and TNNP models; however, as the complexity of the models increases so does the difficulty of obtaining a stability diagram. Though we have not found complete stability diagrams for these detailed ionic models, the representative studies that we have carried out indicate that their stability diagrams are qualitatively similar to that of the Panfilov model, which we have given in Ref. [28]. Here we restrict ourselves to parts of such stability diagrams for the TNNP model for the initial conditions described above. (Similar diagrams for the LRI and RPB models are given in Section 4 of the Supplementary Material S1.) For the TNNP model we present a partial stability diagram for G_CaL_ = 0.000044 in Fig. 9, with a square obstacle of side 27 mm, and all other parameters as specified in the figure captions and in the Supplementary Material S1. (Another partial stability diagram for the TNNP model with G_pCa_ = 3.825 is given in Section 4 of the Supplementary Material S1). These partial stability diagrams suggest that the boundaries between ST, RS, and Q states in the TNNP, LRI, and RPB models are as complicated as in the simple Panfilov model. Thus, as we have stated earlier, spiral-wave dynamics in all these models depends very sensitively on the position of a conduction inhomogeneity.

#### Ionic Inhomogeneities

Apart from obstacles that arise from inhomogeneities in the inter-cellular coupling, cardiac tissue can contain other types of inhomogeneities that originate from changes in single-cell properties, caused say by changes in the chemical environment or metabolic modifications [21], [46]. We refer to a collection of such cells, with slightly modified properties like conductances of ion channels, as ionic inhomogeneities; they are different from the obstacles discussed so far in as much as the spiral waves can enter the region spanned by an ionic inhomogeneity. However, such inhomogeneities do affect the dynamics of spiral waves through cardiac tissue [47], [48]. In this subsection we investigate spiral-wave dynamics in the presence of ionic inhomogeneities in the four models we have discussed above. For the simple Panfilov model, which does not account for ion channels explicitly, we mimic ionic inhomogeneities via modifications of the inverse time ε_1_. We insert such inhomogeneities in LRI, RPB, and TNNP models by considering spatial variations in conductances of calcium ion channels.

In the Panfilov model (1) 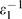 is one of the recovery time constants [12]. As ε_1_ increases the absolute refractory period of the action potential decreases. In turn this decreases the pitch of the spiral wave (cf. Fig. 3 in Ref. [35]). Thus by introducing inhomogeneities in ε_1_ we can investigate spiral-wave dynamics in the presence of time-scale inhomogeneities. If the square simulation domain, of linear size L = 200 mm, is homogeneous, then with ε_1_>0.03 we obtain a single, periodically rotating spiral wave but, as it decreases, for instance, if ε_1_≲0.02, the tip of this rotating spiral starts meandering so that the temporal evolution of the system is quasiperiodic. At even lower values, say at ε_1_ = 0.01 that we have used above, we see spatiotemporal chaos. These behaviors are shown in the illustrative pseudocolor plots of V in Section 5 of the Supplementary Material S1.

We now introduce a square inhomogeneity in ε_1_ in the Panfilov model (all other parameters are uniform over the simulation domain): ε_1_ is assigned the value 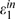 inside a square region; and outside this square it has the value 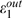. Different choices of 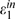 and 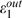 lead to interesting spiral-wave dynamics. For example, with a square patch of side 40 mm, 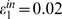 and 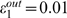, we obtain spatiotemporal chaos for most positions of this inhomogeneity; but for certain critical positions of this inhomogeneity all spiral waves are completely eliminated; e.g., when the inhomogeneity is at (x = 130 mm, y = 80 mm), spiral waves move towards the boundaries of the simulation domain where they are eventually absorbed. For yet other positions spatiotemporal chaos is obtained outside the inhomogeneity but inside it the spiral wave shows a quasiperiodic temporal evolution. Representative plots are given in Section 5 of the Supplementary Material S1.

**Figure 10:**
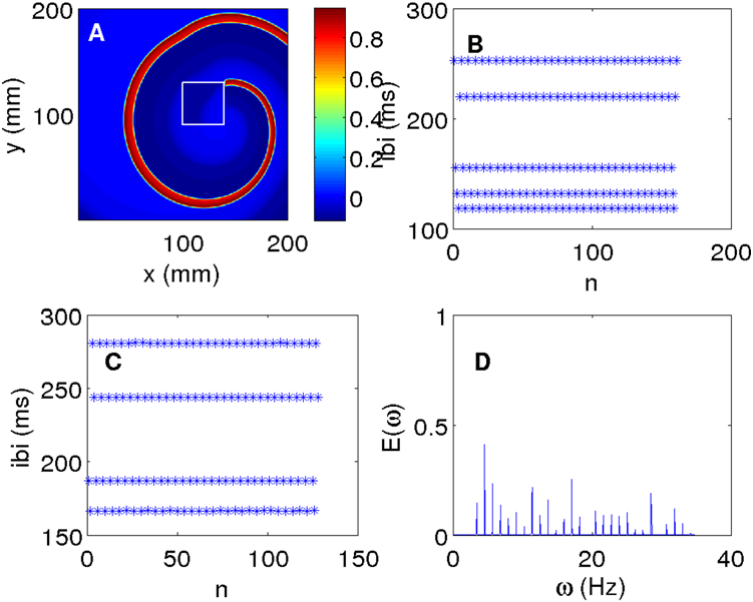
(Color online) Inhomogeneities in the parameter ε_1_ in the Panfilov model can result in a spiral wave anchoring to the inhomogeneity: With 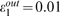 and 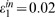 (see text) and a square inhomogeneity of side 40 mm, we see a spiral wave anchored to the inhomogeneity if it is placed at (100 mm, 90 mm). The pseudocolor plot of V in (A) is at t = 2200 ms. The plots of the IBI associated with time series taken from a point outside the inhomogeneity and that from a point inside the inhomogeneity versus the beat number n are given in (B) and (C) respectively; these show period-5 and period-4 behaviors; the power spectrum associated with (C) is given in (D).

If, instead, 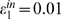 and 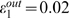 or 0.03, spiral-wave break up occurs inside the inhomogeneity but it coexists with unbroken periodically rotating spiral waves outside it (see Section 5 of the Supplementary Material S1) as noted previously by Xie, *et al.* [31]. However, even in this case, in certain positions such an inhomogeneity anchors a single rotating spiral wave (Fig. 10) as we have seen above, and in Ref. [28], with conduction inhomogeneities; the temporal evolution of V, at a representative point in the simulation domain, is richer than it is with a conduction inhomogeneity: the time series for V can show period-m behavior (we have found cases with 4≤m≤10) as shown in Figs. 10 A–D for periods m = 5 and m = 4. For example, the plot of the interbeat interval (IBI) versus the beat number n in Fig. 10 D jumps periodically between 167, 280, 244 ms, and 187 ms, i.e., the time series for V displays a period-4 cycle. Such period-m behavior has been reported earlier in experiments [49]; in these experiments it is attributed to the interplay of an anchored spiral wave around very small, but nearby, conduction inhomogeneities.

It is natural to ask whether the rich spatiotemporal dynamics of spiral waves in the Panfilov model with inhomogeneities in ε_1_, which lead to inhomogeneities in local times scales since 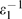 has the dimensions of time, have any analogs in the realistic LRI, RPB, and TNNP models. In cardiac tissue similar changes in time scales can occur because of inhomogeneities in ionic conductances. For example, by changing the conductances G_si_ and G_k_ in the LRI model we can modify the action potential and the time scales associated with it, such as its duration and the extent of the plateau region, which in turn affect spiral-wave dynamics in much the same way as alterations of ε_1_ do in the Panfilov model. In particular, we can effect transitions from periodic to quasiperiodic rotating spiral waves or from quasiperiodic rotating spiral waves to broken waves with spatiotemporal chaos by changing these conductances; moreover, inhomogeneities in these conductances lead to spatiotemporal patterns in LRI, RPB, and TNNP models that are reminiscent of those we have described above for the Panfilov model with inhomogeneities in ε_1_. We show this in detail below by investigating the effects of changes of the maximal calcium and potassium conductances, G_si_ and GK, respectively, in the LRI model, of Gsi in the RPB model, and of the maximal conductance GCaL for the L-type calcium current in the TNNP model. Illustrative simulations for the LRI and RPB models with ionic inhomogeneities are given in Section 5 of the Supplementary Material S1. Here we present our results for ionic inhomogeneities in the TNNP model.

**Figure 11:**
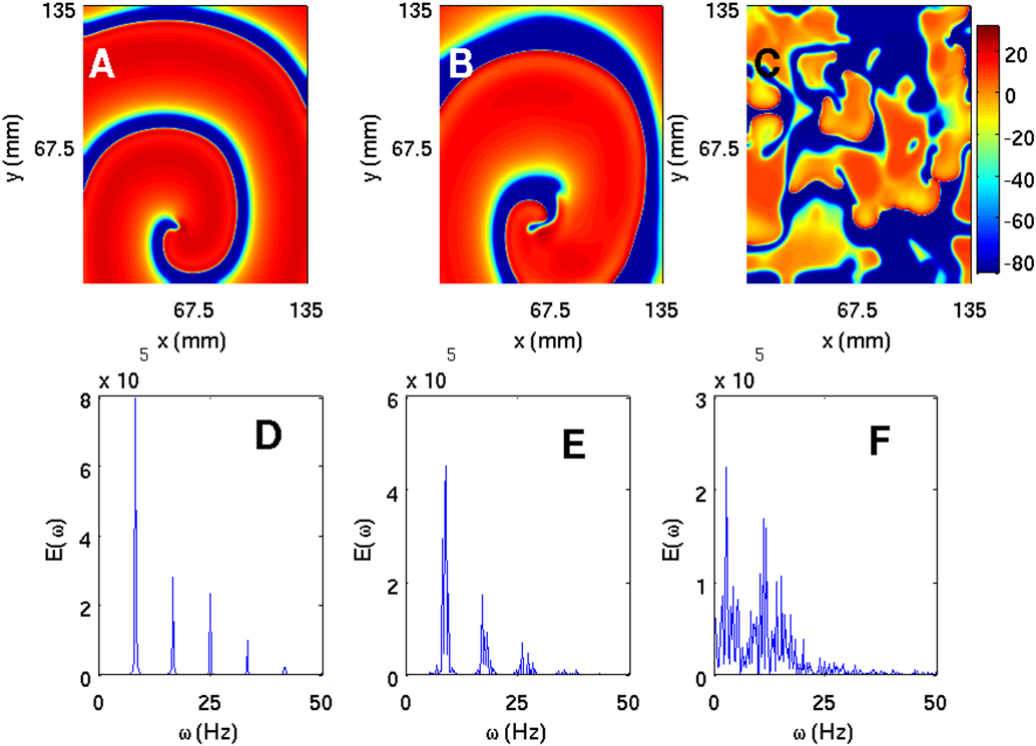
(Color online) The effect of G_CaL_ on spiral waves in the TNNP model shown via pseudocolor plots of V at time t = 3.2 s and (A) G_CaL_ = 0.000175, (B) G_CaL_ = 0.00011, and (C) G_CaL_ = 0.00005. Panels (D), (E), and (F) show the corresponding power spectra of V (from a time series of 200000 iterations after the removal of the initial 80000 iterations) from the representative point (90 mm, 90 mm) in the simulation domain. These plots indicate that, as G_CaL_ decreases, the system goes from a state with a single rotating spiral wave to the spiral-turbulence state.

To investigate ionic inhomogeneities in the TNNP model we consider the calcium conductance G_CaL_ that governs the ionic current I_CaL_ (in the Supplementary Material S1 see Section 2 C and Fig. 30 that shows how the action potential (AP) is modified at the single-cell level as we lower G_CaL_ from 0.000175, the maximal channel conductance, to 0.00011, then to 0.00005, and finally to 0 [i.e., I_CaL_ channel block]). We now study the TNNP model in a square simulation domain of side 13.5 cm with the initial condition of Fig. 1 (A). As we decrease G_CaL_ the spiral wave breaks up because the slope of the APD restitution curve steepens and eventually exceeds 1: This break up is shown in the pseudocolor plots of V, at t = 3.2 s, of Figs. 11 A, B, and C for G_CaL_ = 0.000175, 0.00011, and 0.0005, respectively. In Figs. 11 D, E, and F we show power spectra that have been obtained from time series of V recorded from the representative point (90 mm, 90 mm) during spiral-wave activity for G_CaL_ = 0.000175, 0.00011, and 0.00005, respectively. The discrete lines in the power spectrum of Figs. 11 D can be indexed by one fundamental frequency ≃8.25 Hz and integer multiples thereof; this is a signature of the periodic rotation of a single rotating spiral wave. The multiple strong peaks and the broad-band background in the power spectra of Figs. 11 E and F are indicative of quasiperiodic (with three fundamental frequencies ω_1_≃8.25 Hz, ω_2_≃9 Hz, and ω_3_≃9.5 Hz) and chaotic states, the latter associated with the break up of spiral waves. We get similar results if we increase the plateau Ca^2+^ conductance G_pCa_ instead of changing the L-type Ca^2+^ conductance.

**Figure 12:**
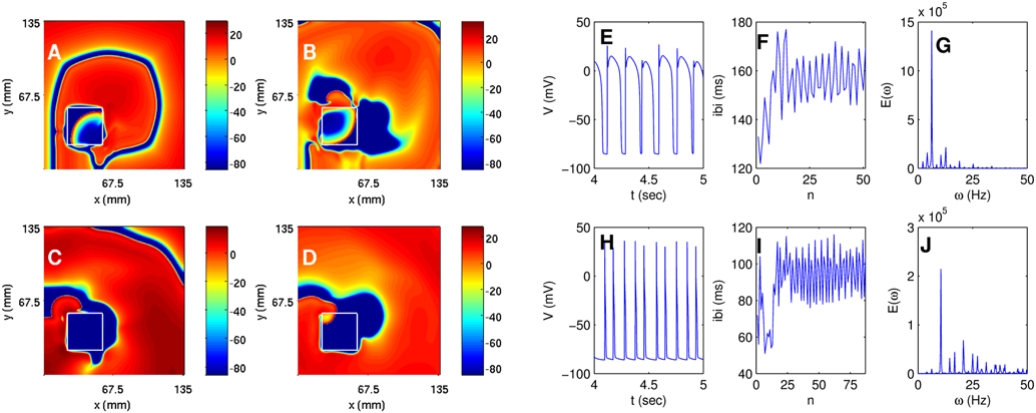
(Color online) The effect of a square GCaL inhomogeneity, of side 33.75 mm in a square simulation domain of side 135 mm, with 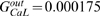 (maximal value) and 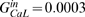, and placed at (22.5 mm, 22.5 mm), on spiral-wave dynamics in the TNNP model: Pseudocolor plots of V at (A) 0.08 s, (B) 0.32 s, (C) 1.2 s, and (D) 2 s; (E) the time series of V (from a sample of 50000 iterations after the removal of the initial 200000 iterations) taken from the point (11.25 mm, 11.25 mm) that lies outside the inhomogeneity. Associated plots of (F) the IBI versus the beat number n (a sample of 400000 iterations) and (G) the power spectrum of V (from a sample of 200000 iterations after the removal of the initial 200000 iterations) indicating quasiperiodic temporal evolution [the peaks in the power spectrum can be indexed (see text) in terms of three incommensurate frequencies (2.25, 4.25, and 6.25 Hz)]. Figures (H), (I), and (J) are the analogs of (E), (F), and (I), respectively, when data for *V* are recorded from the point (33.75 mm, 33.75 mm) that lies inside the inhomogeneity; here too we have quasiperiodic temporal evolution with three underlying incommensurate frequencies (4, 6.25, and 10.5 Hz).

We now insert a square G_CaL_ inhomogeneity of side 33.75 mm in a square simulation domain of side 135 mm and 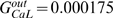 (maximal value) and 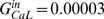, which is approximately one-sixth of its maximal value. When this inhomogeneity is placed at (22.5 mm, 22.5 mm), we observe quasiperiodic behaviors both inside and outside it: Time series for V are recorded from representative points inside and outside of the inhomogeniety, namely, (33.75 mm, 33.75 mm) and (11.25 mm, 11.25 mm), respectively. From these time series we obtain the plots of the IBI and power spectra shown in Fig. 12; we find, in particular, that the main peaks can be indexed as n_1_ω_1_+n_2_ω_2_+n_3_ω_3_ with n_1_, n_2_, and n_3_ integers and ω_1_≃4 Hz, ω_2_≃6.25 Hz, and ω_3_≃10.5 Hz (inside the inhomogeneity), and ω_1_≃2.25 Hz, ω_2_≃4.25 Hz, and ω_3_≃6.25 Hz (outside the inhomogeneity). Since these frequencies are not related to each other by simple rational numbers we conclude that the spiral wave rotates quasiperiodically both inside and outside the inhomogeneity. In some cases we observe that the inhomogeneity does not have a significant qualitative effect on the dynamics of spiral waves; e.g., when the obstacle is at (45 mm, 45 mm), the position of the spiral tip shifts towards the bottom-left corner of the simulation domain but we still have a state with a single rotating spiral wave whose arms pass through the inhomogeneity. Like conduction inhomogeneities, ionic inhomogeneity can also remove spirals from the medium to leave the system in a quiescent state, e.g., when our G_CaL_ ionic inhomogeneity is at (45 mm, 22.5 mm). (See Fig. 33 in the Supplementary Material S1.)

### Elimination of Spiral Turbulence

As we have mentioned above, there is growing consensus that the breakup of spiral waves of electrical activation in ventricular tissue leads to ventricular fibrillation (VF). In the usual clinical treatment of VF electrical stimuli are applied to the affected heart. This is believed to reset all irregular waves in the ventricular tissue leaving it ready to receive the regular sinus rhythm [50]; thus, if the electrical stimulus is strong enough, it can arrest VF and restore the sinus rhythm. Initially 60 Hz AC was used clinically to defibrillate transthrorasically [51] but this was later discontinued because of several reasons including the high energy requirement, the possible induction of atrial fibrillation, the prolonged muscle contraction, the risk of an electrical shock to the operator, and the size of the device [50]. Clinically available defibrillation techniques still apply massive electrical shocks to the heart; this can damage the heart muscle. The success rate of such techniques is not quite satisfactory [52]. Furthermore, scar tissues can be created during the process of such defibrillation; these can make the patient vulnerable to further arrhythmias and also act as conduction inhomogeneities that we have investigated via numerical simulations in the Section on “Results”. Hence there is a great need for developing low-amplitude defibrillation schemes; this must be based on an understanding of the spatiotemporal behavior of activation waves during VF. We begin with a brief overview of some techniques that have been proposed for the elimination of spiral-wave turbulence in models for cardiac tissue. We first examine their efficacy in the homogeneous case; in the next Section we study the relative merits of two low-amplitude defibrillation schemes in the presence of inhomogeneities in our simulation domains. In the context of our numerical simulations of the Panfilov, LRI, RPB, and TNNP models we will use the term defibrillation to mean the elimination of spiral waves or broken spiral waves from the simulation domain.

Early attempts at controlling spiral-wave turbulence in models for cardiac tissue focused on applying well-known techniques of controlling low-dimensional chaos, which are based on the principle that, if a system's trajectory in state space comes very close to an unstable fixed point, it stays in its vicinity for a small duration of time. Different methods have been proposed to drive chaotic trajectories towards such an unstable fixed point, so that the system can stay in the neighborhood of the fixed point for the duration of the control stimulus [53]. But, as we have seen above, the mathematical analog of VF is a state with spiral-wave turbulence. This state displays spatiotemporally chaos, and is, therefore, intrinsically associated with a high-dimensional attractor, so it cannot be controlled by algorithms that have been designed to control chaos in low-dimensional systems.

#### Review of Previous Work

Biktashev and Holden [54] have proposed a method for controlling spiral-wave turbulence by producing a directed movement of a rigidly rotating spiral wave away from the medium by using resonant stimulation. They find that small-amplitude, spatially uniform, repeated stimuli can be used to produce a directed movement of the spiral wave, if the period of stimulation is equal to the period of its rotation. This directed movement eventually pushes this wave out of the simulation domain [54]. However, this method can only be used before the onset of spiral-wave turbulence.

Osipov and Collins [55] have suggested another scheme that is based on the observation that the dynamics of excitable media can be modelled by fast and slow variables, e.g, V and g in the Panfilov model. They control the slow variable by applying a weak impulse on the whole medium. This eventually changes the velocities of the front and back of the wave. The propagation of the wave front and wave back with different velocities leads to a shrinkage or expansion of the pulse width. If the amplitude and duration of the impulse are sufficiently large, then the propagating pulse collapses and disappears. Unfortunately such control of the slow variable over the whole medium can be achieved only by pharmaceutical means and not by the application of electrical pulses.

Rappel, Fenton, and Karma [56] have proposed another method based on the application of a small control current at a finite number of equally spaced “controlled cells” in a tissue, by using a coarse lattice of electrodes with a lattice spacing of about 1 cm. This method has been demonstrated to prevent one spiral from breaking up. Unfortunately this method fails in the fully developed spiral-wave turbulence state with broken spirals [32].

To suppress a spatiotemporally chaotic state with broken spiral waves, Sinha, Pande, and Pandit [32] have proposed a scheme based on the observation that spiral turbulence does not persist in the hearts of small mammals, if it can at all be initiated [57]. We will use this scheme below, so we describe it in some detail. They have shown that spiral-wave turbulence is a long-lived transient [32], [35] whose lifetime 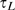 increases rapidly with the linear size L of the simulation domain, e.g., from ≃850 ms for L = 100 mm to ≃3200 ms for L = 128 mm in the two-dimensional Panfilov model; for large systems (e.g., L>128 mm in the Panfilov case), 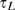 is sufficiently long so that we obtain a nonequilibrium statistical steady state with spatiotemporal chaos [35]. This might suggest that a global control scheme, such as that of Osipov and Collins [55], is essential. It turns out, however, that a judicious choice of control points on a mesh leads to an efficient scheme for the control of spiral-wave turbulence in such models [32]. We first illustrate the principle of this method for a two-dimensional square domain with side L: This is divided into K^2^ smaller blocks by a mesh of line electrodes, and the mesh size is chosen to be small enough that spirals cannot persist for long inside the block of side 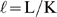. A voltage or current pulse is applied at all points along the mesh boundaries for a time. This makes the mesh region refractory and so effectively simulates Neumann boundary conditions for any block bounded by the mesh. Thus spiral waves formed inside the block are absorbed at the mesh bounding the block. For example, in the Panfilov model in dimension d = 2, L = 128, and K = 2, a time 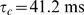 suffices to suppress spiral turbulence; when L = 512, and K = 8, a time 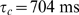 is required; electrical pulses of amplitude ≃60 µA/cm^2^ are used on the control mesh; this is much less than in conventional electrical defibrillation which uses pulses of amplitude 1 A/cm^2^. This control algorithm has been extended to suppress spiral turbulence in the two-dimensional Beeler-Reuter and LRI models [35]. A naϊve extension of this control algorithm to three-dimensions requires a cubic array of sheets, which cannot be implanted easily in ventricular tissue. However, in Ref. [32] it was shown that, even if the control mesh is present only on one of the L×L faces of an L×L×L_z_ domain, the above scheme works with a slight modification: Instead of applying a pulse for a time 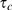, we have to apply a sequence of n pulses, separated by 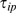; the duration of each pulse is 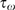. A steady pulse does not control scroll-wave turbulence in three dimensions for L_z_>2 mm: its propagation in the z direction is impeded once the interior of the simulation domain becomes refractory. However, if we use a sequence of short pulses and if 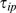 is long enough for the medium to recover its excitability, the control-pulse waves can propagate in the z direction and lead to successful control. We refer the reader to Refs. [32], [35] for further details; we give representative three-dimensional simulations after our discussion of two-dimensional studies.

Recently Zhang, *et al.* [33], have proposed another attractive scheme for the control of spiral turbulence in excitable media. In their method spiral waves are driven away by periodic forcing of 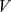 at a small number of n×n points in the center of the simulation domain. This generates target waves that eventually drive out the spiral waves if the amplitude 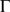 and the frequency ω_f_ of the forcing are chosen carefully: For example, for the Panfilov model with d = 2 it is shown in Ref. [33] that spiral turbulence in a square 500×500 simulation domain can be suppressed within 410,000 iterations when one chooses n = 6, ω_f_ = 0.82, and 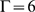. This control scheme is attractive because it employs *local* forcing, compared to the control scheme of Ref. [32] that uses a *spatially extended* control mesh. However, as we show below, the local control scheme of Ref. [33] inadvertently generates spiral-wave break up if there are obstacles in the medium.

#### Summary of Our Results

**Figure 13:**
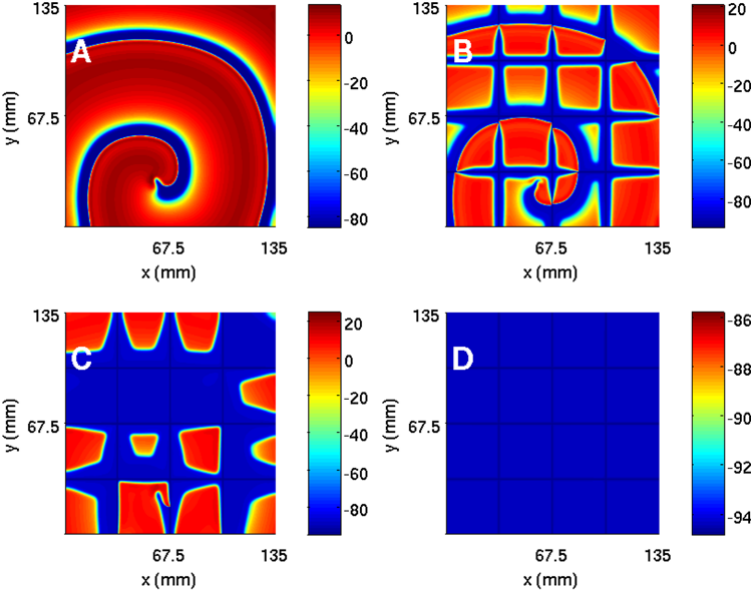
(Color online) Defibrillation by our control scheme in the TNNP model for a homogeneous simulation domain of side L = 135 mm. We apply a control pulse of amplitude 27.75 µA/cm^2^ for t = 20 ms over a square mesh with each block of size L/K = 33.75 mm, i.e., the simulation domain is divided into 4^2^ square blocks. Pseudocolor plots of V at (A) 0 ms, (B) 24 ms, (C) 80 ms, and (D) 280 ms, after the initiation of the control pulse, show how spiral turbulence is suppressed.

The control scheme of Ref. [32] is also successful in eliminating spiral turbulence in the realistic LRI, RPB, and TNNP models that account for ion channels. Illustrative simulations for the LRI and RPB models are given in Section 6 of the Supplementary Material S1. Figure 13 presents results from our simulations of the two-dimensional TNNP model; here a control current of 27.75 µA/cm^2^, applied for 20 ms on a mesh that divides our square simulation domain of side 13.5 cm into 16 square cells of side 3.375 cm each, suffices to control spiral turbulence. The pseudocolor plots of V in Fig. 13 give (A) the initial spiral-turbulent state and its subsequent evolution after the initiation of the control pulse at (B) 24 ms, (C) 80 ms, and (D) 280 ms, after which all spiral turbulence disappears.

As we mentioned above, the control scheme of Ref. [32] can be extended to three-dimensions. For a practical implementation of this scheme the control pulse must be applied only on one face of the simulation domain. This suffices when the thickness in the third dimension is small. For example, in the three-dimensional Panfilov model, even if we apply a control pulse on a mesh on one of the L×L faces of the L×L×L_z_ simulation domain, we can suppress broken scroll waves (the three-dimensional analogs of broken spiral waves) in the entire medium, provided that L_z_<2 mm. Illustrative simulations are given in Section 6 of the Supplementary Material S1.

**Figure 14:**
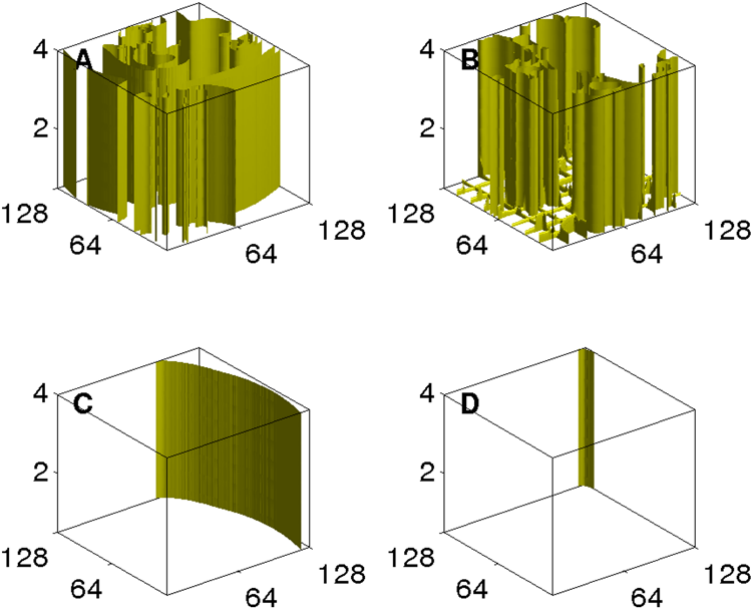
(Color online) The control of scroll-wave turbulence in our simulation of the 3D Panfilov for L_z_ = 4 mm in a domain of size 128×128×4 mm^3^: An iso-surface plot of V shows the initial state in (A). A series of 32 pulses, each lasting for 0.22 ms, and separated by 220 ms is applied on the bottom of the domain on a mesh; the subsequent evolution of of the scroll waves is shown at 440 ms (B), 1760 ms (C), and 1870 ms (D) by which time we see the last wave moving out of the simulation domain.

For L_z_>2 mm this control scheme for a three-dimensional domain must be modified as follows: We use a control mesh on one face, but, instead of using a single long pulse, we use a series of short pulses, each of duration 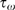 and separated by an interval 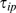; for suitable choices of 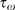 and 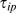, we can effectively suppress spiral turbulence. (A similar scheme, with short pulses separated from each other, also works in two dimensions.) We illustrate this control scheme for the three-dimensional Panfilov model in a 128×128×4 mm^3^ simulation domain, a control mesh of square cells, each of side 16 mm, on the bottom face of the domain, 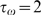 iterations (i.e., 0.22 ms), 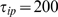 iterations (22 ms), and a pulse strength of 0.48; we use a series of 32 pulses. Figure 14 shows isosurface plots of V with the initial condition (A) and its evolution after the initiation of the control at (B) 440 ms, (C) 1760 ms, and (D) 1870 ms, the time by which we see the last part of a scroll wave moving out of the simulation domain.

### The Control of Spiral-Wave Turbulence in the Presence of Inhomogeneities

In the last Section we have given a short overview of some control schemes that have been used to suppress spiral-wave break up in two- and three-dimensional simulations in some mathematical models for cardiac tissue. Cardiac tissue can have inhomogeneities, such as scar tissue. It is important, therefore, to study whether these control schemes are effective in controlling spiral-wave turbulence in the presence of such inhomogeneities. To the best of our knowledge this has not been investigated systematically so far. We present such an investigation here (for simplicity we restrict ourselves to conduction inhomogeneities). In particular, it is important to ensure that a control scheme does not lead inadvertently to spiral break up in the presence of inhomogeneities.

**Figure 15:**
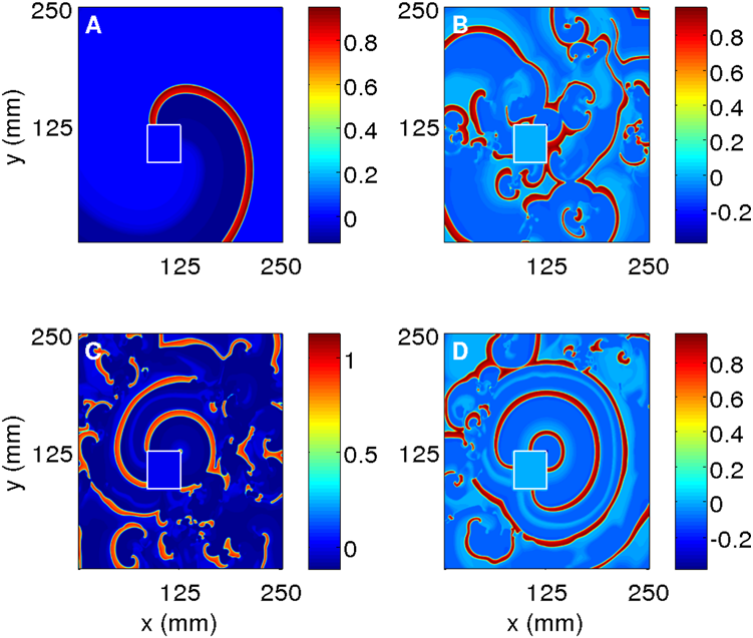
Pseudocolor plots of the transmembrane potential V in the two-dimensional Panfilov model illustrating the application of the local-forcing control scheme of Ref. [33] on a spiral wave anchored to the obstacle as shown in the initial condition (A). The subsequent evolution of the system in the presence of the forcing is shown in (B), (C), and (D). The control stimulus, of the form 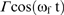 with 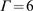 and ω_f_ = 0.82, is applied on a small square region of side 0.3 cm in the center of the simulation domain of side L = 25 cm; the square conduction inhomogeneity of side 4 cm is placed at (8.5 cm, 8.5 cm). The target waves break up and contribute to spiral turbulence in the medium because of the obstacle as shown in (B) immediately after the control is applied and in (C) and (D) after 200,000 and 400,000 iterations, respectively; given our time discretization 1 iteration is 0.11 ms.

We begin by studying the control scheme of Zhang *et al.* [33] that we have outlined above. This scheme drives away broken spiral waves from the simulation domain by using the target waves that are created by the local periodic forcing. What happens to such target waves when they encounter an obstacle? We examine this for the two-dimensional Panfilov model. In Fig. 15 we present our results with such periodic forcing [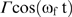 with 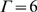 and ω_f_ = 0.82]; we use parameters such that, in the absence of this forcing, a single spiral wave would have been attached to the conduction inhomogeneity (Fig. 15 A that is similar, e.g., to Fig. 1B in Ref. [28]). The forcing we use generates target waves in the center of the simulation domain, of side L = 25 cm and with a square obstacle of side l = 4 cm placed at (8.5 cm, 8.5 cm). It turns out that these target waves drive away the spiral wave that was anchored to the obstacle in the absence of the forcing. The time evolution of the system with the forcing (Figs. 15 B–D) shows the break up of these target waves as they collide with the obstacle and thus contribute to spiral turbulence in the medium [58]. Had there been no obstacle, this control scheme would have driven away all the broken spiral waves from the domain. However, this does not happen in the presence of an obstacle as shown in Fig. 15; hence this control scheme is unsuitable for controlling spiral-wave turbulence if inhomogeneities are present.

**Figure 16:**
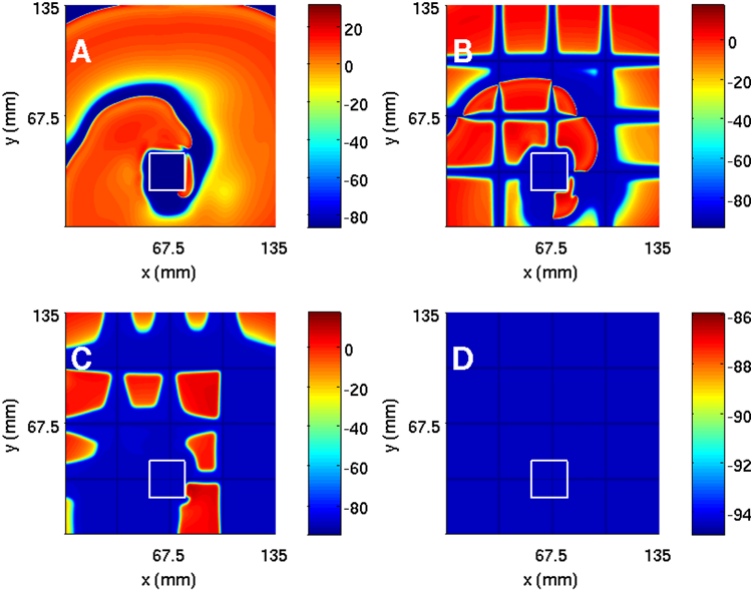
(Color online) Suppressing spiral turbulence in the 2D TNNP model in the presence of an inhomogeneity: A control pulse of amplitude 27.75 µA/cm^2^ is applied for t = 20 ms on a mesh as in Fig. 13. The square obstacle is at (54 mm, 22.5 mm) and has side l = 22.5 mm. Without control the spiral turbulence persists as in Fig. 6. The pseudocolor plots of V in (A) 0 ms, (B) 24 ms, (C) 80 ms, and (D) 280 ms, after the initiation of the control, show the suppression of the spiral turbulence.

By contrast, the control scheme proposed in Ref. [32] works even in the presence of an inhomogeneity. Illustrative simulations for the Panfilov, LRI, and RPB models are given in Section 6 of the Supplementary Material S1. Here we show how the control scheme of Ref. [32] is also successful in eliminating spiral turbulence in the TNNP model even in the presence of conduction inhomogeneities. In Fig. 16 a control current of 27.75 µA/cm^2^ is applied for 20 ms on a mesh that divides our square simulation domain of side 13.5 cm into 16 square cells of side 3.375 cm each. This suffices to control spiral turbulence in the TNNP model, even though there is a square obstacle of side 2.25 cm placed in the simulation domain. (If this obstacle is at (45 mm, 31.5 mm), in the absence of the control pulse, a single rotating spiral wave would have been anchored to this obstacle; if instead the obstacle is at (54 mm, 22.5 mm), as in Fig. 6, spirals would have broken up in the absence of the control.) The pseudocolor plots of V in Fig. 16 give (A) the initial state and its subsequent evolution after the initiation of the control pulse at (B) 24 ms, (C) 80 ms, and (D) 280 ms, after which all spiral turbulence disappears. A similar simulation for the elimination of spiral turbulence in the TNNP model is given in Section 6 of the Supplementary Material S1.

**Figure 17:**
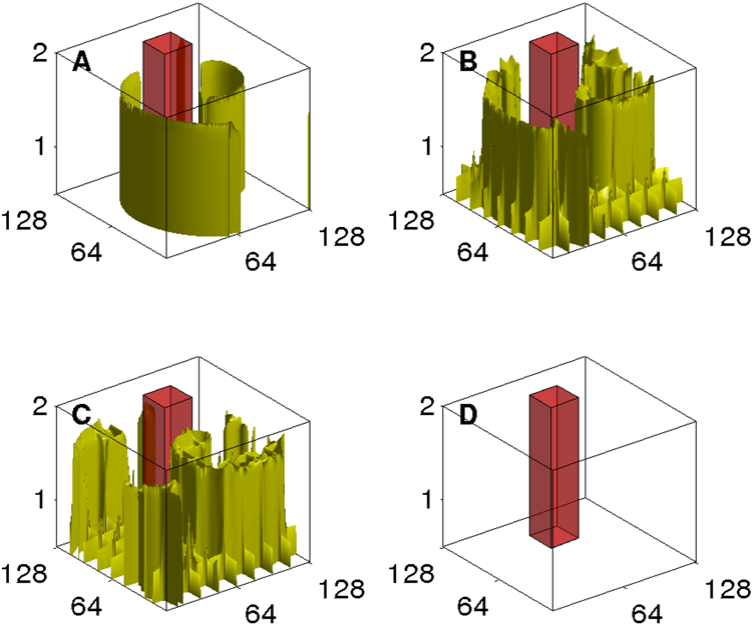
(Color online) Controlling scroll-wave turbulence in our 3D Panfilov-model simulation in a 128×128×2 mm^3^ simulation domain in the presence of an obstacle of size 25×25×2 mm^3^ placed at (80 mm, 60 mm): The scroll wave breaks up in this case as shown by the V iso-surface in (A). We now apply a control pulse of strength 0.48 on a mesh with square cells, each of side 16 mm, on the bottom face of the simulation domain for 748 ms; the evolution of the V iso-surfaces, after the initiation of the control, is depicted at (B) 220 ms, (C) 440 ms, and (D) 880 ms, by which time scroll waves have left the simulation domain and it is completely quiescent.

We have shown above how the control scheme of Ref. [32] can be extended to three-dimensions in an L×L×L_z_ simulation domain, if L_z_<2 mm. This scheme works even in the presence of an obstacle as is illustrated for representative cases in Fig. 17 with an obstacle of size 25×25×2 mm^3^ placed at (80 mm, 60 mm). In the absence of control pulses, scroll-wave turbulence is obtained. We now apply a control pulse of strength 0.48 on a mesh with square cells, each of side 16 mm, on the bottom face of the simulation domain for 748 ms; the evolution of the states of the system after the initiation of the control are depicted at (B) 220 ms, (C) 440 ms, and (D) 880 ms in Fig. 17. By 880 ms the scroll waves have left the simulation domain and it is completely quiescent.

When L_z_>2 mm, the control scheme described in the previous paragraph fails just as its counterpart did in the absence of an inhomogeneity. A representative simulation for this case is given in Section 6 of the Supplementary Material S1.

## Discussion

We have presented the most extensive numerical study carried out so far of the effects of inhomogeneities on spiral-wave dynamics in mathematical models for cardiac tissue. In particular, we have investigated such dynamics in the Panfilov, LRI, RPB, and TNNP models for homogeneous simulation domains and also in the presence of conduction and ionic inhomogeneities. Furthermore, we have considered two low-amplitude control schemes in detail; these have been designed to eliminate spiral-wave turbulence in these models but have not been tested systematically in the presence of inhomogeneities; we carry out such tests here.

One of the principal results of our studies is the confirmation that spiral-wave and scroll-wave dynamics in mathematical models of cardiac tissue depend very sensitively on the positions of conduction or ionic inhomogeneities in the simulation domain. Our results here extend significantly those presented in Ref. [28] for the Panfilov and LRI models. In particular, we have shown that this sensitive dependence on inhomogeneities also holds in realistic ionic models, which account for ion pumps and ion exchangers and also the details of the dynamics of calcium ions; furthermore, these results also hold in three-dimensional simulation domains, as illustrated by our calculations for the three-dimensional Panfilov model; and the nature of the inhomogeneity also affects the spatiotemporal dynamics of spiral waves as can be seen by comparing our simulations of conduction inhomogeneities with those for ionic inhomogeneities. As we have seen, in the latter case the transmembrane potential V displays rich and different temporal behaviors inside and outside the ionic inhomogeneity. We believe this sensitive dependence of spiral waves on inhomogeneities in the medium is a reflection of a fractal basin boundary between the domains of attraction of spiral-turbulence (ST), rotating-spiral (RS), and quiescent (Q) states. In a low-dimensional dynamical system it is possible to obtain such a basin boundary by changing initial conditions; in a high-dimensional dynamical system (the partial-differential-equation models for cardiac tissue are infinite dimensional) it is not practical to find such a boundary numerically. We have shown instead, that, by changing parameters in these cardiac-tissue models, such as the positions or natures of inhomogeneities, we can affect the spatiotemporal evolution of spiral waves drastically.

Our studies have practical implications for experimental investigations of spiral-wave dynamics in cardiac tissue. In particular, the studies of Refs.[22]–[24], [49] have provided a rich variety of results including complicated temporal patterns in inter-beat intervals [49] for V and the partial or complete elimination of spiral-wave turbulence by conduction inhomogeneities [2]. We have described these briefly in the introduction. Here we would like to note that our *in silico* simulations of spiral-wave dynamics in the Panfilov, LRI, RPB, and TNNP models have allowed us to carry out a much more systematic study of inhomogeneities in these models than is possible *in vitro* and *in vivo*. We hope our work will stimulate experiments in this field. It is worth noting that our study yields all the types of rich spatiotemporal behaviors (e.g., for V) that have been observed in a variety of experiments on spiral-wave dynamics in cardiac tissue or cell cultures, if we keep in mind that the states ST, RS, and Q in our simulations are the analogs of VF, VT, and quiescence in such experiments.

Our results, especially those on the elimination of spiral-wave turbulence in the presence of inhomogeneities, should also have important implications for the development of low-amplitude electrical defibrillation schemes, which is a major challenge that lies at the interfaces between nonlinear science, biophysics, and biomedical engineering. One of the lessons of our numerical studies, namely, the sensitive dependence of spiral-wave dynamics on inhomogeneities, implies that low-amplitude defibrillation schemes might well have to be tuned suitably to account for inhomogeneities in cardiac tissue. Furthermore, it would be very interesting to develop the mesh-based control scheme that we have described in the previous section and to see how it might be realised experimentally.

While this paper was being prepared for publication two new suggestions appeared for the elimination of spiral turbulence in models such as the LRI model [59], [60]. The first of these [59] uses a square lattice of control points through which a control voltage is swept. Since this uses a spatially extended set of control points, it is successful in the elimination of spiral turbulence at least in a two-dimensional domain. The study of Ref. [60] examines a scheme that requires the use of a bidomain model since relatively large control voltages are used. We have concentrated instead on low-amplitude defibrillation schemes that should not require bidomain models; indeed such schemes have been used earlier by several groups [32], [33], [56], [61], [62] without bidomain models.

As we have emphasized throughout this paper, one of the principal goals of our study is a qualitative one, namely, the elucidation of the sensitive dependence of spiral-wave dynamics on inhomogeneities in mathematical models of cardiac tissue. We have, therefore, carried out extensive simulations of such dynamics in the Panfilov, LRI, RPB, and TNNP models; but we have not, so far, extended our study to bidomain models [63] and models in which mechanics [64] is also included. We expect our principal qualitative results about inhomogeneities will go through even when such models are considered; this will have to be checked explicitly by subsequent studies.

In our earlier 3D simulations [28] we have studied the effect of inhomogeneities on scroll waves; in this case too we find that scroll-wave dynamics depends sensitively on the position of an inhomogeneity. Here too we discuss some 3D simulations. However, we have concentrated on 2D simulations for two reasons: (a) it is important to have a good understanding of spiral-wave dynamics in the presence of inhomogeneities before embarking on similar, detailed, 3D simulations; (b) in 3D studies it is important to include tissue anisotropy (but again we believe this can be done systematically only after we have a good understanding of the 2D simulations we have presented here). In the same spirit of studying the simplest models first, we have not considered models which include mechanics too; indeed this purely electrical approach has been adopted by several other studies (see, e.g., Refs. [11], [12], [14], [25], [30]–[33]) with the understanding that the mechanical system basically follows the electrical activation at the level of a first approximation (see, e.g., Ref. [60]).

## Supporting Information

Supplementary Material S1 Spiral-Wave Turbulence and its Control in the Presence of Inhomogeneities in Four Mathematical Models of Cardiac Tissue
(3.99 MB PDF)
