# Supplementary material for: Spiral-Wave Turbulence and Its Control in the Presence of Inhomogeneities in Four Mathematical Models of Cardiac Tissue
Source: PLoS One. 2009 Mar 9;4(3):e4738. doi: 10.1371/journal.pone.0004738 (PMC2650787; doi:10.1371/journal.pone.0004738)
Supplement: Supplementary Material S1 — Spiral-Wave Turbulence and its Control in the Presence of Inhomogeneities in Four Mathematical Models of Cardiac Tissue (3.99 MB PDF) [file pone.0004738.s001.pdf]

Supplementary Material S1- Spiral-Wave Turbulence and its Control in the Presence of Inhomogeneities in Four Mathematical Models of Cardiac Tissue

T.K. Shajahan[1,2], Alok Ranjan Nayak[1], and Rahul Pandit[1,*]

[1]Centre for Condensed Matter Theory, Department of Physics,
Indian Institute of Science, Bangalore 560012, INDIA

[2]Indian Institute of Science Eduation and Research (IISER), Thiruvananthapuram,
CET Campus, Thiruvananthapuram 695016, Kerala, INDIA

1 Introduction

This supplementary material for the manuscript "Spiral-Wave Turbulence and its Control in the Presence of Inhomogeneities in Four Mathematical Models of Cardiac Tissue" is organized as follows. In Section 2 we give the complete partial differential equations for the Luo-Rudy Phase 1 (LRI), reduced-Priebe-Beuckelmann (RPB), and ten Tusscher, Noble, Noble, and Panfilov (TNNP) models. Section 3 contains details of the results of our numerical simulations for Panfilov, LRI, and RPB models in homogeneous simulation domains; similar results for the TNNP model are contained in the main paper (Sec. on "Results"). Section 4 contains details of the results of our numerical simulations for Panfilov, LRI, and RPB models with conduction inhomogeneities in the simulation domain; similar results for the TNNP model are contained in the main paper (Sec. on "Results"). Section 5 describes the results of our numerical simulations for Panfilov, LRI, and RPB models with ionic inhomogeneities in the simulation domain; similar results for the TNNP model are contained in the main paper (Sec. on "Results"). Section 6 is devoted to our numerical studies of schemes for the control of spiral turbulence in Panfilov, LRI, and RPB models in homogeneous domains or in the presence of inhomogeneities in the simulation domain; similar results for the TNNP model and for three-dimensional domains are contained in the main paper (Sec. on "Results").

2 Models

A The Luo-Rudy Model

In the Luo-Rudy I (LR I) model there are six components of the ionic current, which are formulated mathematically in terms of Hodgkin-Huxley-type equations[1]. The partial differential equation for the transmembrane potential V is

$$\frac{\partial V}{\partial t} + \frac{I_{LR}}{C} = D\nabla^2 V. \tag{1}$$

Here I_{LR} is the instantaneous, total ionic-current density. The subscript LR denotes that we use the formulation of the total ionic current described by the Luo-Rudy Phase I (LR1) model [2], where $I_{LR} = I_{Na} + I_{si} + I_K + I_{K1} + I_{Kp} + I_b$, with the current densities I_{Na} (fast inward Na^+), I_{si} (slow inward), I_K

[*]Also at Jawaharlal Nehru Centre for Advanced Scientific Research, Bangalore 560064, INDIA

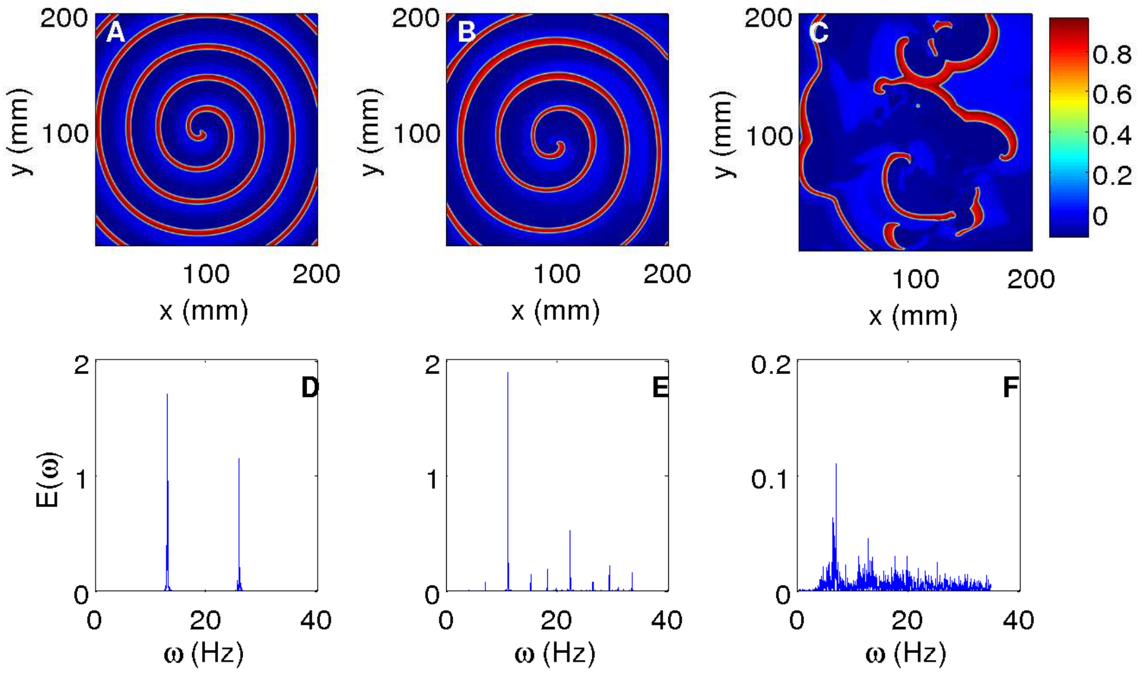

Figure 1: (Color online) The effects of changing ϵ_1 in the Panfilov model shown via pseudocolor plots (A)-(C) of V after 3300 ms and the associated power spectra (D)-(F), obtained from time series of V from a representative point in the square simulation domain of side $L = 200$ mm: (D) When $\epsilon_1 = 0.03$, the power spectrum shows discrete lines that can be indexed in terms of one fundamental frequency ($\simeq 13.04$ Hz); (E) if $\epsilon_1 = 0.02$, the power-spectrum peaks can be indexed as $n_1\omega_1 + n_2\omega_2$, with the two frequencies $\omega_1 \simeq 7.09$ Hz and $\omega_2 \simeq 11.19$ Hz not rationally related to each other, i.e., the system evolves quasiperiodically in time; (F) if ϵ_1=0.01, the spiral waves break up and the power spectrum shows a broad-band background, a clear indication of chaotic behavior.

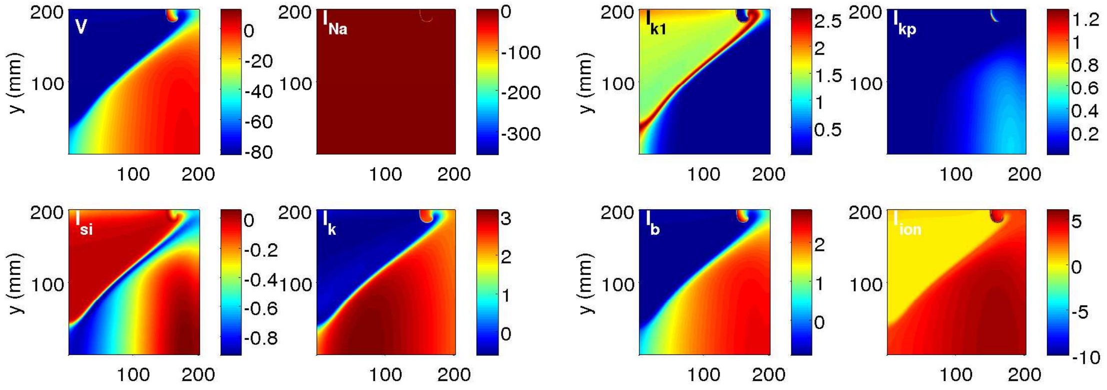

Figure 2: Pseudocolor plots of initial spatial distributions of the transmembrane voltages V and the currents I_{Na}, I_{si}, I_k, I_{k1}, I_{kp}, I_b, and I_{ion} in our numerical simulation of the LRI model in a square domain with $L = 90$ mm.

(slow outward *time-dependent* K^+), I_{K_1} (*time-independent* K^+), I_{Kp} (plateau K^+), I_b (total background), given by:

$$\begin{aligned}
I_{Na} &= G_{Na} m^3 hj (V - E_{Na}); & (2)\\
I_{si} &= G_{si} df (V - E_{si}); & (3)\\
I_K &= G_K x x_i (V - E_K); & (4)\\
I_{K_1} &= G_{K_1} K_{1\infty} (V - E_{K_1}); & (5)\\
I_{Kp} &= G_{Kp} K_p (V - E_{Kp}); & (6)\\
I_b &= 0.03921 (V + 59.87); & (7)
\end{aligned}$$

and $K_{1\infty}$ is the steady-state value of the gating variable K_1. All current densities are in units of μA/cm^2, voltages are in mV, and G_ξ and E_ξ are, respectively, the ion-channel conductance and reversal potential for the channel ξ. The ionic currents are determined by the time-dependent ion-channel gating variables h, j, m, d, f, x, x_i, K_p and K_1 generically denoted by ξ, which follow ordinary differential equations of the type

$$\frac{d\xi}{dt} = \frac{\xi_\infty - \xi}{\tau_\xi}, \qquad (8)$$

where $\xi_\infty = \alpha_\xi/(\alpha_\xi + \beta_\xi)$ is the steady-state value of ξ and $\tau_\xi = \frac{1}{\alpha_\xi + \beta_\xi}$ is its time constant. The voltage-dependent rate constants, α_ξ and β_ξ, are given by the following empirical equations:

$$\begin{aligned}
\alpha_h &= 0, \text{ if } V \geq -40 \text{ mV}, & (9)\\
&= 0.135 \ \exp[-0.147\ (V + 80)], \text{ otherwise;} &\\
\beta_h &= \frac{1}{0.13\ (1 + \exp[-0.09 (V + 10.66)])}, \text{ if } V \geq -40 \text{ mV}, & (10)\\
&= 3.56 \ \exp[0.079\ V] + 3.1 \times 10^5 \ \exp[0.35\ V], \text{ otherwise;} &
\end{aligned}$$

$$\begin{aligned}
\alpha_j &= 0, \text{ if } V \geq -40 \text{ mV}, & (11)\\
&= [\frac{(\exp[0.2444\ V] + 2.732 \times 10^{-10} \ \exp[-0.04391\ V])}{-7.865 \times 10^{-6}\{1 + \exp[0.311\ (V + 79.23)]\}}] &\\
&\quad \times (V + 37.78), \text{ otherwise;} &\\
\beta_j &= \frac{0.3 \ \exp[-2.535 \times 10^{-7}\ V]}{1 + \exp[-0.1\ (V + 32)]}, \text{if } V \geq -40 \text{ mV}, & (12)\\
&= \frac{0.1212 \ \exp[-0.01052\ V]}{1 + \exp[-0.1378\ (V + 40.14)]}, \text{ otherwise;} &
\end{aligned}$$

$$\begin{aligned}
\alpha_m &= \frac{0.32\ (V + 47.13)}{1 - \exp[-0.1\ (V + 47.13)]}; & (13)\\
\beta_m &= 0.08 \ \exp[-0.0909\ V]; & (14)
\end{aligned}$$

$$\begin{aligned}
\alpha_d &= \frac{0.095 \ \exp[-0.01\ (V - 5)]}{1 + \exp[-0.072\ (V - 5)]}; & (15)\\
\beta_d &= \frac{0.07 \ \exp[-0.017\ (V + 44)]}{1 + \exp[0.05\ (V + 44)]}; & (16)
\end{aligned}$$

$$\alpha_f = \frac{0.012 \ \exp[-0.008 \ (V+28)]}{1+\exp[0.15 \ (V+28)]}; \tag{17}$$
$$\beta_f = \frac{0.0065 \ \exp[-0.02 \ (V+30)]}{1+\exp[-0.2 \ (V+30)]}; \tag{18}$$

$$\alpha_x = \frac{0.0005 \ \exp[0.083 \ (V+50)]}{1+\exp[0.057 \ (V+50)]}; \tag{19}$$
$$\beta_x = \frac{0.0013 \ \exp[-0.06 \ (V+20)]}{1+\exp[-0.04 \ (V+20)]}; \tag{20}$$

$$\alpha_{K1} = \frac{1.02}{1+\exp[0.2385 \ (V-E_{K1}-59.215)]}; \tag{21}$$
$$\beta_{K1} = \frac{[0.49124 \ \exp[0.08032 \ (V-E_{K1}+5.476)]}{1+\exp[-0.5143 \ (V-E_{K1}+4.753)]} + \exp[0.06175 \ (V-E_{K1}-594.31]]. \tag{22}$$

The gating variables x_i and K_p are given by

$$x_i = \frac{2.837 \ \exp 0.04(V+77)-1}{(V+77)\exp 0.04 \ (V+35)}, \text{if } V > -100\text{mV},$$
$$= 1, \text{ otherwise}; \tag{23}$$
$$K_p = \frac{1}{1+\exp[0.1672 \ (7.488-V)]}. \tag{24}$$

The values of the channel conductances G_{Na}, G_{si}, G_K, G_{K_1}, and G_{Kp} are 23, 0.07, 0.705, 0.6047 and 0.0183 mS/cm^2, respectively[3]. The reversal potentials are $E_{Na} = 54.4$ mV, $E_K = -77$ mV, $E_{K1} = E_{Kp} = -87.26$ mV, $E_b = -59.87$ mV, and $E_{si} = 7.7 - 13.0287 \ln Ca$, where Ca is the calcium ionic concentration satisfying

$$\frac{dCa}{dt} = -10^{-4} I_{si} + 0.07(10^{-4} - Ca). \tag{25}$$

The times t and τ_ξ are in ms; the rate constants α_ξ and β_ξ are in ms^{-1}.

B The Reduced Priebe-Beuckelmann Model

The reduced Priebe Beuckelmann (RPB) Model is described in detail in Ref. [4]. In this model the partial differential equation for the transmembrane potential V is

$$\frac{\partial V}{\partial t} + \frac{I_{RPB}}{C} = D\nabla^2 V. \tag{26}$$

Here I_{RPB} is the instantaneous, total ionic-current density. The subscript RPB denotes that we use the formulation of the total ionic current described by the RPB model, where $I_{RPB} = I_{Na} + I_{Ca} + I_{to} + I_k + I_{k1} + I_{Na,Ca} + I_{Na,K} + I_{Na,b} + I_{Ca,b}$, with the current densities I_{Na} (fast inward Na^+ current), I_{Ca} (slow inward Ca^{2+} current), I_{to} (transient outward current), I_k (delayed rectifier K^+ current), I_{k1} (inward rectifier K^+ current), $I_{Na,K}$ (Na^+-K^+ pump current), $I_{Na,Ca}$ (Na^+/Ca^{2+} exchanger current, $I_{Na,b}$ (Na^+ background current), and $I_{Ca,b}$ (Ca^{2+} background current). They are given by:

$$I_{Na} = G_{Na} m^3 h^2 (V - E_{Na}); \tag{27}$$

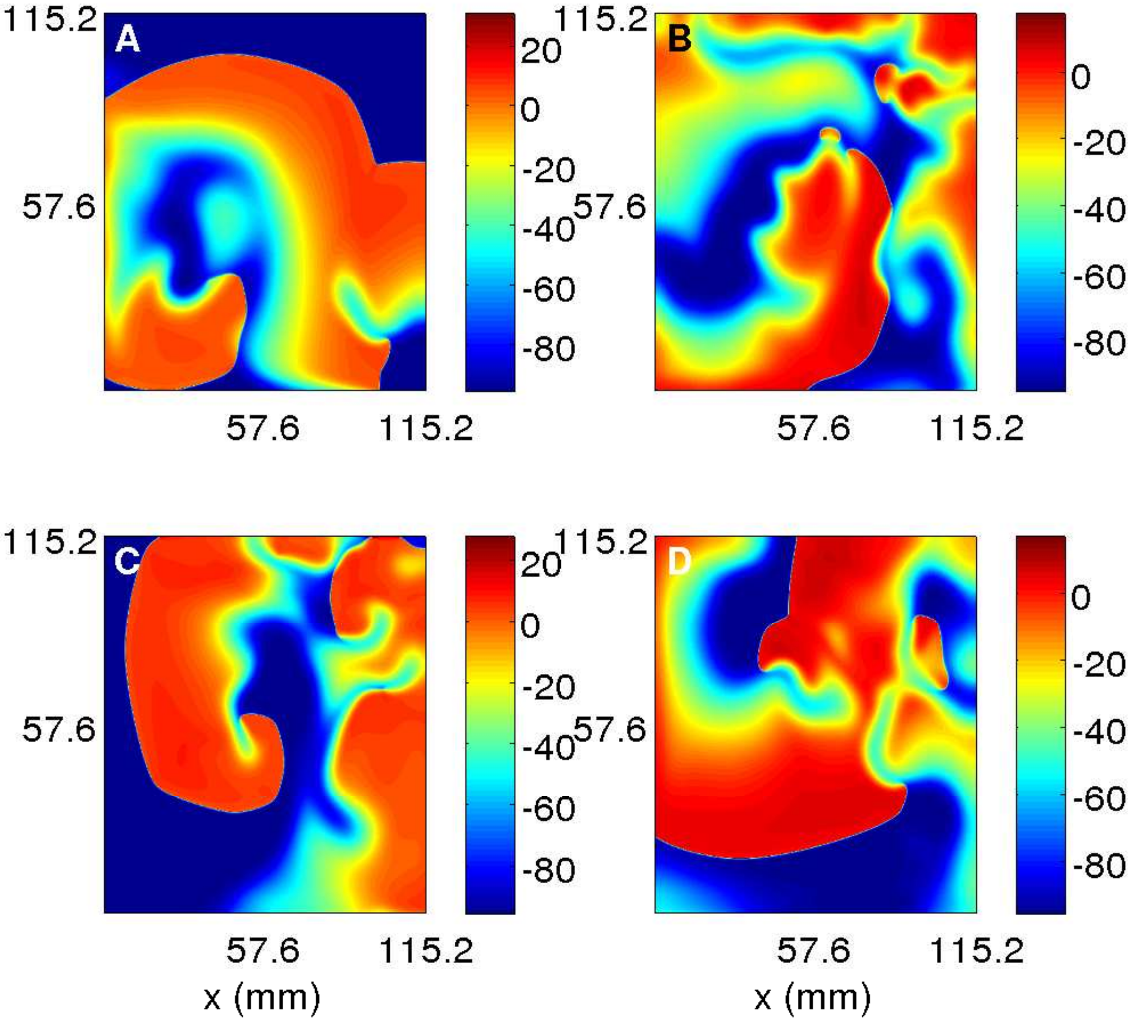

Figure 3: (Color online) Spiral turbulence in the RPB model from our simulation in a square domain with $L = 115.2$ mm: Pseudocolor plots of the transmembrane potential V showing (A) the initial condition, set up as described in the text, and its subsequent evolution after (B) 1200 ms, (C) 1400 ms, and (D) 1800 ms.

$$
\begin{aligned}
I_{Ca} &= G_{Ca} d_\infty f_{Ca} f (V - E_{Ca}); && (28)\\
I_{to} &= G_{to} t r_\infty (V - E_{to}); && (29)\\
I_k &= G_k x^2 (V - E_k); && (30)\\
Ik1 &= Gk1 K1_\infty (V - Ek); && (31)\\
I_{Na,K} &= G_{Na,K} f_{Na,K} f'_{Na,K}; && (32)\\
I_{Ca,b} &= G_{Ca,b} (V - E_{Ca}); && (33)\\
I_{Na,b} &= G_{Na,b} (V - E_{Na}); && (34)
\end{aligned}
$$

Here G_{Na}, G_{Ca}, G_{to}, G_k, G_{k1}, $G_{Na,K}$, $G_{Ca,b}$, $G_{Na,b}$ are maximal conductances of the respective ionic currents.

The current through each ion channel is controlled by a set of gates; the fraction of gates open is denoted by the gating variables. The gate f_{Ca} inactivates the Ca^{2+} channel depending on the concentration of Ca^{2+} ions; the fraction (of these gates) that are open is given by

$$
f_{Ca} = \left(1 + \frac{[Ca^{2+}]_i}{0.0006}\right)^{-1}. \qquad (35)
$$

Most other gates are voltage-dependent and have two gating variables; one gets activated during the action potential and the other one gets inactivated: m and h are voltage-dependent activation and inactivation gates for I_{Na}, d and f for I_{Ca}, and r and t for I_{to}; x is the activation gate for I_k and K_1 the inactivation gate of I_{k1}. The rates at which these gates open or close are determined by the rate constants, denoted generically by α_ξ and β_ξ. The gating variables, generically denoted by ξ, follow ordinary differential equations of the type

$$
\frac{d\xi}{dt} = \alpha_\xi (1 - \xi) - \beta_\xi \xi, \qquad (36)
$$

which can be rewritten as

$$
\frac{d\xi}{dt} = \frac{\xi_\infty - \xi}{\tau_\xi}, \qquad (37)
$$

where $\xi_\infty = \alpha_\xi/(\alpha_\xi + \beta_\xi)$ is the steady-state value of ξ and $\tau_\xi = \frac{1}{\alpha_\xi + \beta_\xi}$ is its time constant, and the rate constants depend on the transmembrane potential as follows:

$$
\begin{aligned}
\alpha_m &= 0.32(V + 47.13)/(1.0 - \exp(-0.1(V + 47.13))); && (38)\\
\beta_m &= 0.08 \exp(-V/11.0); && (39)\\
h_\infty &= 0.5(1 - \tanh(7.74 + 0.12V)); && (40)\\
h_\tau &= 0.25 + 2.24 \frac{(1 - \tanh(7.74 + 0.12V))}{(1 - \tanh(0.07(V + 92.4)))}; && (41)
\end{aligned}
$$

$$
\begin{aligned}
\alpha_d &= 14.98 \frac{\exp(-0.5[(V - 22.36)/16.68]^2)}{(16.68\sqrt{(}2\pi))}; && (42)\\
\beta_d &= 0.1471 - 5.3 \frac{\exp(-0.5[(V - 6.27)/14.93]^2)}{(14.93\sqrt{(}2\pi))}; && (43)\\
\alpha_f &= 0.00687/(1.0 + \exp(-(6.1546 - V)/6.12)); && (44)
\end{aligned}
$$

$$\beta_f = \frac{(0.069\exp(-0.11(V+9.825))+0.011)}{(1+\exp(-0.278(V+9.825)))} + 5.75.10^{-4}; \quad (45)$$

$$\alpha_r = 0.5266\frac{\exp(-0.0166(V-42.2912))}{(1+\exp(-0.0943(V-42.2912)))}; \quad (46)$$

$$\beta_r = 0.5149\frac{\exp(-0.1344(V-5.0027))+5.186.10^{-5}V}{(1+\exp(-0.1348(V-5.18610^{-5})))}; \quad (47)$$

$$\alpha_t = \frac{(0.0721\exp(-0.173(V-V_{shift}+34.2531))+5.61210^{-5}(V-V_{shift}))}{(1+\exp(-0.1732(V-V_{shift}+34.2531)))}; \quad (48)$$

$$\beta_t = \frac{(0.0767\exp(-1.6610^{-9}(V-V_{shift}+34.0235))+1.21510^{-4}(V-V_{shift}))}{(1+\exp(-0.1604(V-V_{shift}+34.0235)))}; \quad (49)$$

$$t_\tau = p_{inv}/(\alpha_t+\beta_t); \quad (50)$$

$$x_\infty = 0.972/(1+\exp(-2.036-0.0834V)); \quad (51)$$

here p_{inv} is the scale factor for the inactivation time constant for the transient outward potassium current (I_t). Its value is 1.7 for myocardial cells. V_{shift} is the shift in the half-inactivation voltage of I_t and is equal to -4 mV in myocardial cells.

$$x_\tau = 380\exp(-(25.5+V)(25.5+V)/156)+166(1+\tanh(0.558+0.0169V)); \quad (52)$$

$$\alpha_{k1} = 0.1/(1+\exp(0.06(V-Ek-200))); \quad (53)$$

$$\beta_{k1} = \frac{(3.0\exp(0.0002(V-Ek+100))+\exp(0.1(V-Ek-10)))}{(1+\exp(-0.5(V-Ek)))}; \quad (54)$$

the voltage dependence parameter f_{Nak} in the pump current I_{Nak} is given by;

$$f_{Nak} = 1/(1+0.1245\exp(-0.0037V)+0.0365\exp(-0.037V)); \quad (55)$$

σ, the Na_o^+ dependence factor of I_{Nak}, is given by

$$\sigma = 0.1428(\exp(Na_o/67.3)-1); \quad (56)$$

f'_{Nak} depends on the concentration of Na^+ and K^+ ions:

$$f'_{Nak} = (1/(1+Na_i)^{1.5}))((K_o/K_i)+1.5); \quad (57)$$

$$I_{Naca} = G_{Naca}f_{Naca}; \quad (58)$$

The voltage-dependence parameter f_{Naca} in the $Na-Ca$ exchanger current I_{Naca} is given by the equation:

$$f_{Naca} = \frac{(Na_{ic}Ca_o\exp(0.013V)-Na_{ic}Ca_i\exp(-0.024V))}{((669921.875+Na_{oc})(1.38+Ca_o)(1+0.1\exp(-0.024V)))}; \quad (59)$$

equilibrium potentials in this model are:

$$
\begin{aligned}
E_{Na} &= (RT/F)\ln([Na^+]_o/[Na^+]_i); & (60)\\
E_{Ca} &= (RT/2F)\ln([Ca^{2+}]_o/[Ca^{2+}]_i); & (61)\\
E_{to} &= (RT/F)\ln(\frac{0.043[Na^+]_o + [K^+]_o}{(0.043[Na^+]_i + [K^+]_i)}); & (62)\\
E_K &= (RT/F)\ln(\frac{[K^+]_o}{[K^+]_i}); & (63)
\end{aligned}
$$

here R is the universal gas constant, T is the absolute temperature, F is the Faraday constant, and $[Na^+]_o, [Ca^{2+}]_o$, and $[K^+]_o$ are the extracellular concentrations of Na^+, Ca^{2+} and K^+ concentrations, respectively.

C The TNNP model

The reaction-diffusion equation for the transmembrane potential V in the TNNP model is

$$
\frac{\partial V}{\partial t} + \frac{I_{\text{TNNP}}}{C_m} = D\nabla^2 V, \quad (64)
$$

where C_m is the membrane capacitance density. The subscript TNNP denotes that we use the formulation of the total ionic currents described in the Ten Tusscher-Noble-Noble-Panfilov model.

$$
\begin{aligned}
I_{ion} = \; & I_{Na} + I_{CaL} + I_{to} + I_{Ks} + I_{Kr} + I_{K1} \quad (65)\\
& + I_{NaCa} + I_{NaK} + I_{pCa} + I_{pK} + I_{bNa} + I_{bCa},
\end{aligned}
$$

where I_{Na} is the fast inward Na^+ current, I_{CaL} the L-type slow inward Ca^{2+} current, I_{to} the transient outward current, I_{Ks} the slow delayed rectifier current, I_{Kr} the rapid delayed rectifier current, I_{K1} the inward rectifier K^+ current, I_{NaCa} the Na^+/Ca^{2+} exchanger current, I_{NaK} the Na^+/K^+ pump current, I_{pCa} and I_{pK} the plateau Ca^{2+} and K^+ currents, and I_{bNa} and I_{bCa} the background Na^+ and Ca^{2+} currents.

Physical units used in the model are as follows: time (t) is in milliseconds, the voltage (V) in millivolts, the current densities (I_X) in picoamperes per picofarad, the conductances (G_X) in nanosiemens per picofarad, and the intracellular and extracellular ionic concentrations (X_i, X_o) in millimoles per liter. Area and capacitance are related since the specific capacitance of cardiac tissue is of the order of $1\mu\text{A/cm}^2$. The equations for the reversal potentials, currents, and gating variables in the TNNP model are given below. The 12 gating variables are m, h, j, d, f, f_{Ca}, r, s, x_s, x_{r1}, x_{r2}, g and the associated rate constants are α_m, β_m, etc.

Reversal Potentials

The reversal potentials obey the Nernst equation:

$$
\begin{aligned}
E_X &= \frac{RT}{zF} log \frac{X_o}{X_i} \quad \text{for } X = Na^+,\ K^+,\ Ca^{2+} & (66)\\
E_{Ks} &= \frac{RT}{F} log \frac{K_o + p_{KNa}Na_o}{K_i + p_{KNa}Na_i}, & (67)
\end{aligned}
$$

where R is the gas constant, T the temperature, z the valence of the ion, F the Faraday constant, X_o and X_i the extra- and intra-cellular ionic concentrations, p_{KNa} the relative I_{Ks} permeability to Na^+.

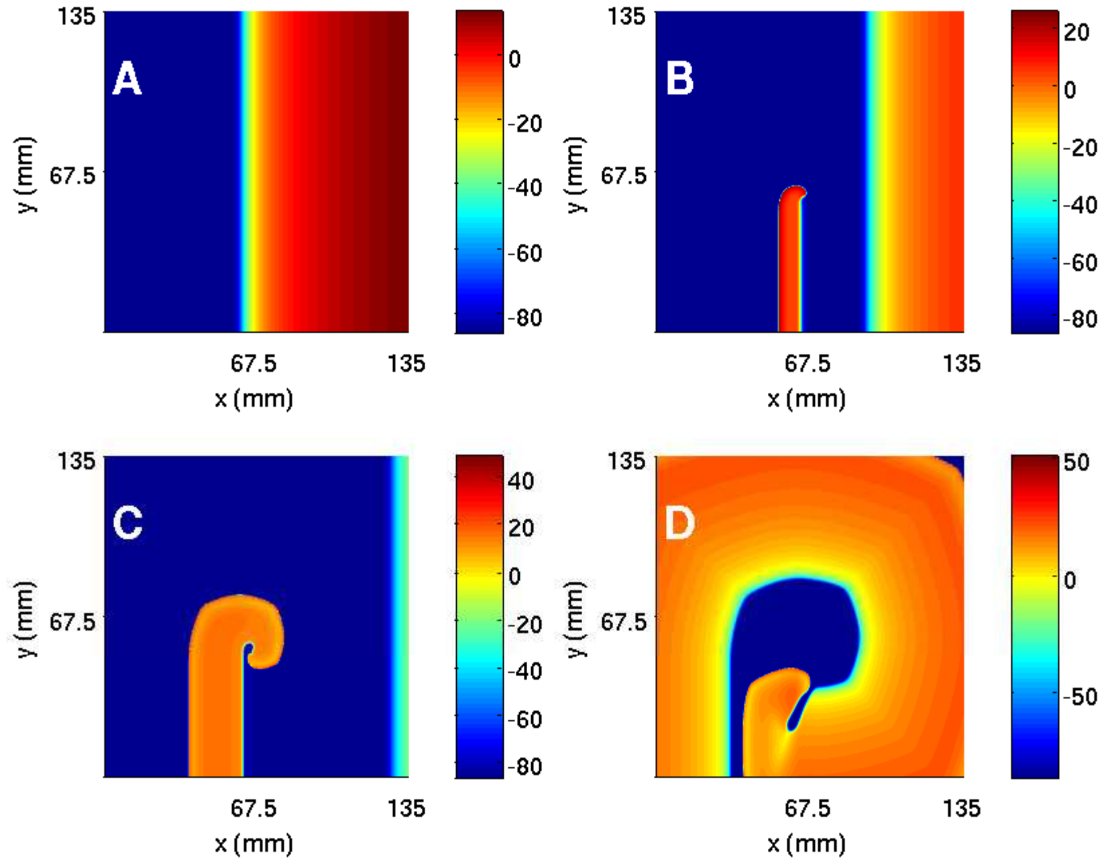

Figure 4: (Color online) Initiation of spiral waves in the TNNP model in a square simulation domain of side $L = 135$ mm (see text) illustrated via pseudocolor plots of the transmembrane potential V at (A) $t = 264$ ms, (B) $t = 300$ ms, (C) $t = 336$ ms, and (D) $t = 524$ ms. We use the configuration shown in (D) as our initial condition to study spiral-waves dynamics in this model.

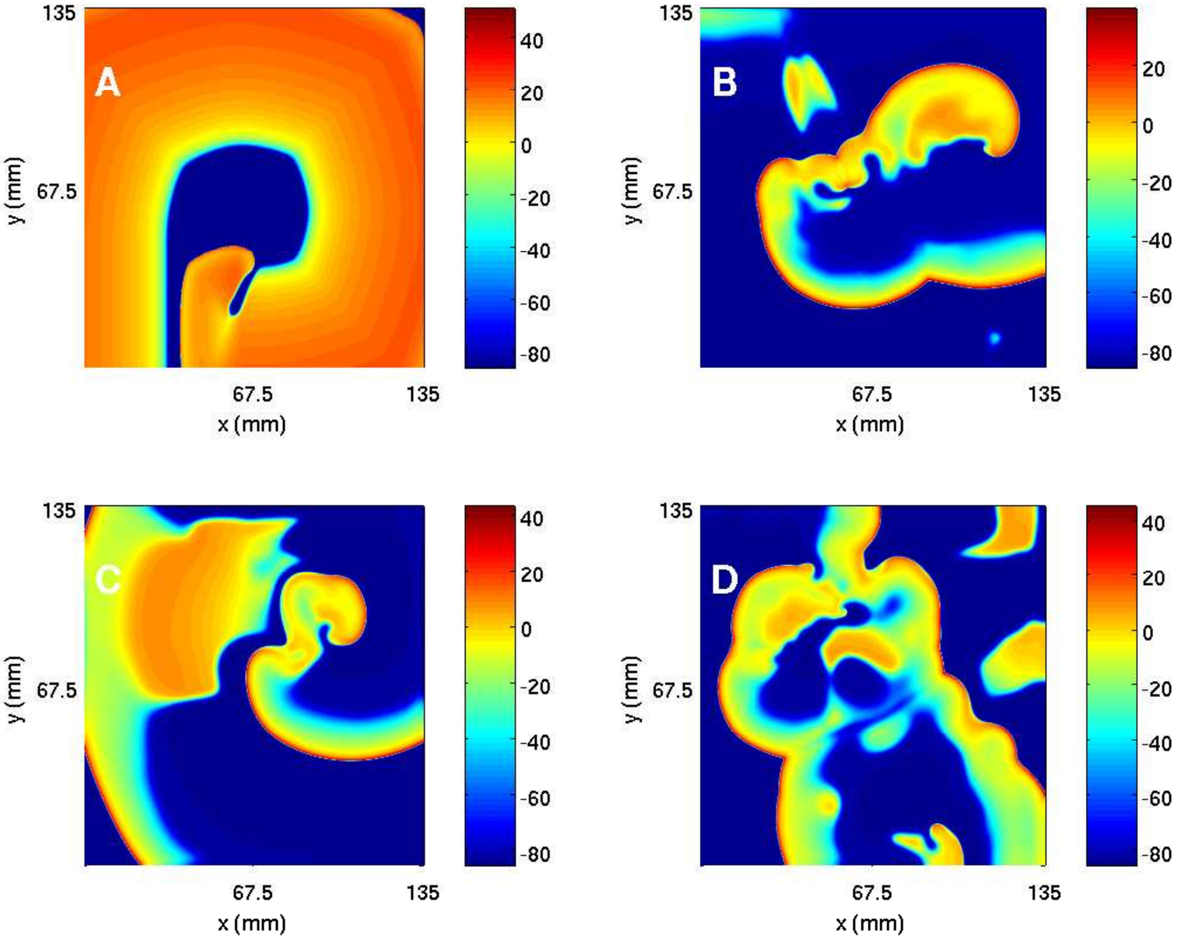

Figure 5: Spiral turbulence in the TNNP model in a square simulation domain, with side $L = 135$ mm and $G_{CaL} = 0.000044$ (all other parameters as in Sec. 2C), illustrated via pseudocolor plots of the transmembrane potential V at (A) $t = 0$ s, (B) $t = 0.8$ s, (C) $t = 1.6$ s, and (D) $t = 2.4$ s.

Fast Na^+ Current

$$I_{Na} = G_{Na}m^3hj(V - E_{Na}) \quad (68)$$

$$m_\infty = \frac{1}{\left[1+\exp(\frac{-56.86-V}{9.03})\right]^2} \quad (69)$$

$$\alpha_m = \frac{1}{1+\exp(\frac{-60-V}{5})} \quad (70)$$

$$\beta_m = \frac{0.1}{1+\exp(\frac{V+35}{5})} + \frac{0.1}{1+\exp(\frac{V-50}{200})} \quad (71)$$

$$\tau_m = \alpha_m\beta_m \quad (72)$$

$$h_\infty = \frac{1}{\left[1+\exp(\frac{V+71.55}{7.43})\right]^2} \quad (73)$$

$$\alpha_h = 0 \quad \text{if} \quad V \geq -40 \quad (74)$$

$$= 0.057\text{exp}\left(\frac{-(V+80)}{6.8}\right) \quad \text{(otherwise)}$$

$$\beta_h = \frac{0.77}{0.13\left[1+\exp\left(\frac{-(V+10.66)}{11.1}\right)\right]} \quad \text{if} \quad V \geq -40 \quad (75)$$

$$= 2.7\text{exp}(0.079V) + 3.1\times10^5\text{exp}(0.3485V) \quad \text{(otherwise)}$$

$$\tau_h = \frac{1}{\alpha_h+\beta_h} \quad (76)$$

$$j_\infty = \frac{1}{\left[1+\exp(\frac{V+71.55}{7.43})\right]^2} \quad (77)$$

$$\alpha_j = 0 \quad \text{if} \quad V \geq -40 \quad (78)$$

$$= \frac{\left(-2.5428\times10^4\exp(0.2444V) - 6.948\times10^{-6}\exp(-0.04391V)\right)(V+37.38)}{1+\exp\left(0.311(V+79.23)\right)} \quad \text{(otherwise)}$$

$$\beta_j = \frac{0.6\exp(0.057V)}{1+\exp\left(-0.1(V+32)\right)} \quad \text{if} \quad V \geq -40 \quad (79)$$

$$= \frac{0.02424\text{exp}(-0.01052V)}{1+\exp\left(-0.1378(V+40.14)\right)} \quad \text{(otherwise)}$$

$$\tau_j = \frac{1}{\alpha_j+\beta_j} \quad (80)$$

L-type Ca^{2+} Current

$$I_{CaL} = G_{CaL}dff_{Ca}4\frac{VF^2}{RT}\frac{Ca_i\text{exp}(\frac{2VF}{RT}) - 0.0341Ca_o}{\text{exp}(\frac{2VF}{RT})-1} \quad (81)$$

$$d_\infty = \frac{1}{1+\exp(\frac{-5-V}{7.5})} \quad (82)$$

$$\alpha_d = \frac{1.4}{1+\exp(\frac{-35-V}{13})} + 0.25 \quad (83)$$

$$\beta_d = \frac{1.4}{1+\exp(\frac{V+5}{5})} \quad (84)$$

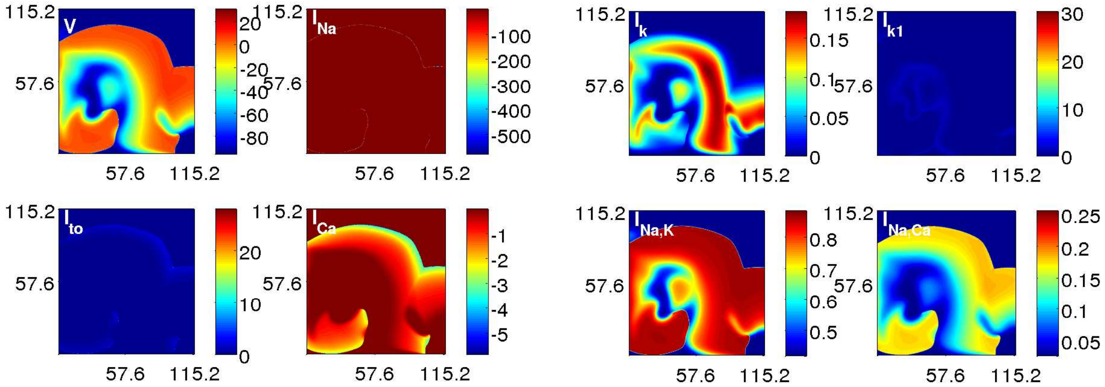

Figure 6: (Color online) Pseudocolor plots of the initial spatial distribution of the transmembrane potential V and the currents I_{Na}, I_{to}, I_{si}, $I_k, I_{k1}, I_{Na,K}$, and $I_{Na,Ca}$ in our simulation of the RPB model in a square domain with side $L = 115.2$ mm.

$$\gamma_d = \frac{1}{1+\exp(\frac{50-V}{20})} \tag{85}$$

$$\tau_d = \alpha_d\beta_d + \gamma_d \tag{86}$$

$$f_\infty = \frac{1}{1+\exp(\frac{V+20}{7})} \tag{87}$$

$$\tau_f = 1125\text{exp}\left(\frac{-(V+27)^2}{240}\right) + \frac{165}{1+\exp(\frac{25-V}{10})} + 80 \tag{88}$$

$$\alpha_{fCa} = \frac{1}{1+\left(\frac{Ca_i}{0.000325}\right)^8} \tag{89}$$

$$\beta_{fCa} = \frac{0.1}{1+\exp(\frac{Ca_i-0.0005}{0.0001})} \tag{90}$$

$$\gamma_{fCa} = \frac{0.2}{1+\exp(\frac{Ca_i-0.00075}{0.0008})} \tag{91}$$

$$f_{Ca\infty} = \frac{\alpha_{fCa}+\beta_{fCa}+\gamma_{fCa}+0.23}{1.46} \tag{92}$$

$$\frac{df_{Ca}}{dt} = k\frac{f_{Ca\infty}-f_{Ca}}{\tau_{fCa}} \tag{93}$$

$$k = 0 \quad \text{if} \quad f_{Ca\infty} > f_{Ca} \quad \text{and} \quad V > -60\ mV$$

$$= 1 \quad \text{(otherwise)}$$

$$\tau_{fCa} = 2\ ms \tag{94}$$

Transient Outward Current

$$I_{to} = G_{to}rs(V-E_K) \tag{95}$$

$$r_\infty = \frac{1}{1+\exp(\frac{20-V}{6})} \tag{96}$$

$$\tau_r = 9.5\exp\left(\frac{-(V+40)^2}{1800}\right) + 0.8 \quad (97)$$

$$s_\infty = \frac{1}{1+\exp(\frac{V+28}{5})} \quad \text{for endocardial cell types} \quad (98)$$

$$= \frac{1}{1+\exp(\frac{V+20}{5})} \quad \text{for epicardial and M}-\text{cell types} \quad (99)$$

$$\tau_s = 1000\exp\left(\frac{-(V+67)^2}{1000}\right) + 8 \quad \text{for endocardial cell types} \quad (100)$$

$$= 85\exp\left(\frac{-(V+45)^2}{320}\right) + \frac{5}{1+\exp(\frac{V-20}{5})} + 3 \quad \text{for epicardial and M}-\text{cell types} \quad (101)$$

Slow Delayed Rectifier Current

$$I_{Ks} = G_{Ks}{x_s}^2(V - E_{Ks}) \quad (102)$$

$$x_{s\infty} = \frac{1}{1+\exp(\frac{-5-V}{14})} \quad (103)$$

$$\alpha_{xs} = \frac{1100}{\sqrt{1+\exp(\frac{-10-V}{6})}} \quad (104)$$

$$\beta_{xs} = \frac{1}{1+\exp(\frac{V-60}{20})} \quad (105)$$

$$\tau_{xs} = \alpha_{xs}\beta_{xs} \quad (106)$$

Rapid Delayed Rectifier Current

$$I_{Kr} = G_{Kr}\sqrt{\frac{K_o}{5.4}}x_{r1}x_{r2}(V - E_K) \quad (107)$$

$$x_{r1\infty} = \frac{1}{1+\exp(\frac{-26-V}{7})} \quad (108)$$

$$\alpha_{xr1} = \frac{450}{1+\exp(\frac{-45-V}{10})} \quad (109)$$

$$\beta_{xr1} = \frac{6}{1+\exp(\frac{V+30}{11.5})} \quad (110)$$

$$\tau_{xr1} = \alpha_{xr1}\beta_{xr1} \quad (111)$$

$$x_{r2\infty} = \frac{1}{1+\exp(\frac{V+88}{24})} \quad (112)$$

$$\alpha_{xr2} = \frac{3}{1+\exp(\frac{-60-V}{20})} \quad (113)$$

$$\beta_{xr2} = \frac{1.12}{1+\exp(\frac{V-60}{20})} \quad (114)$$

$$\tau_{xr2} = \alpha_{xr2}\beta_{xr2} \quad (115)$$

Inward Rectifier K^+ Current

$$I_{K1} = G_{K1}\sqrt{\frac{K_o}{5.4}}x_{K1\infty}(V - E_K) \quad (116)$$

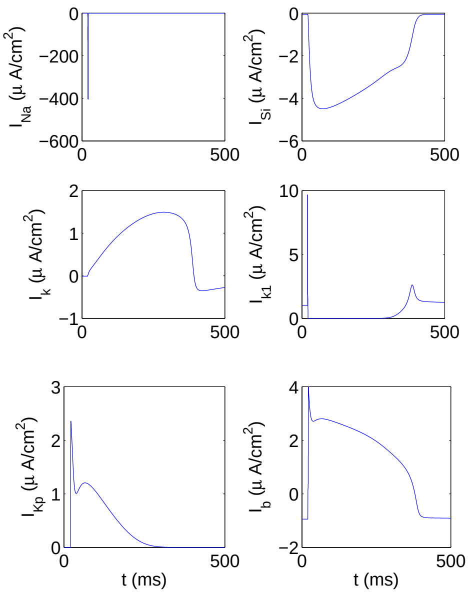

Figure 7: Plots showing the dependence of the currents I_{Na}, I_{si}, I_k I_{k1}, I_{kp} and I_b on time t during the course of the action potential from our single-cell simulation of the LRI model. Negative currents move into the cell and positive currents move out of the cell.

$$\alpha_{K1} = \frac{0.1}{1+\exp\left(0.06(V-E_K-200)\right)} \tag{117}$$

$$\beta_{K1} = \frac{3\exp\left(0.0002(V-E_K+100)\right)+\exp\left(0.1(V-E_K-10)\right)}{1+\exp(-0.5(V-E_K))} \tag{118}$$

$$X_{K1\infty} = \frac{\alpha_{K1}}{\alpha_{K1}+\beta_{K1}} \tag{119}$$

Na^+/Ca^{2+} Exchanger Current

$$I_{NaCa} = k_{NaCa}\frac{\exp\left(\frac{\gamma VF}{RT}\right){Na_i}^3 Ca_o - \exp\left(\frac{(\gamma-1)VF}{RT}\right){Na_o}^3 Ca_i \alpha}{({K_{mNai}}^3+{Na_o}^3)(K_{mCa}+Ca_o)\left(1+k_{sat}\exp\left(\frac{(\gamma-1)VF}{RT}\right)\right)} \tag{120}$$

Na^+/K^+ Pump Current

$$I_{NaK} = P_{NaK}\frac{K_o Na_i}{(K_o+K_{mK})(Na_i+K_{mNa})\left(1+0.1245\exp\left(\frac{-0.1VF}{RT}\right)+0.0353\exp\left(\frac{-VF}{RT}\right)\right)} \tag{121}$$

Plateau Currents

$$I_{pCa} = G_{pCa}\frac{Ca_i}{K_{pCa}+Ca_i} \tag{122}$$

$$I_{pK} = G_{pK}\frac{V-E_K}{1+\exp(\frac{25-V}{5.98})} \tag{123}$$

Background Currents

$$I_{bNa} = G_{bNa}(V - E_{Na}) \quad (124)$$
$$I_{bCa} = G_{bCa}(V - E_{Ca}) \quad (125)$$

Calcium Dynamics

$$I_{leak} = V_{leak}(Ca_{sr} - Ca_i) \quad (126)$$
$$I_{up} = \frac{V_{maxup}}{1 + \left(\frac{K_{up}}{Ca_i}\right)^2} \quad (127)$$
$$I_{rel} = \left(a_{rel}\frac{{Ca_{sr}}^2}{{b_{rel}}^2 + {Ca_{sr}}^2} + c_{rel}\right)dg \quad (128)$$
$$g_\infty = \frac{1}{1 + \left(\frac{Ca_i}{0.00035}\right)^6} \quad \text{if} \quad Ca_i \leq 0.00035 \quad (129)$$
$$= \frac{1}{1 + \left(\frac{Ca_i}{0.00035}\right)^{16}} \quad \text{(otherwise)}$$
$$\tau_g = 2\ ms \quad (130)$$
$$\frac{dg}{dt} = k\frac{g_\infty - g}{\tau_g} \quad (131)$$
$$k = 0 \quad \text{if} \quad g_\infty > g \quad \text{and} \quad V > -60\ mV$$
$$= 1 \quad \text{(otherwise)}$$
$$Ca_{ibufc} = \frac{Ca_i \times Buf_c}{Ca_i + K_{bufc}} \quad (132)$$

$$\frac{dCa_{itotal}}{dt} = -\frac{I_{CaL} + I_{bCa} + I_{pCa} - 2I_{NaCa}}{2V_C F} + I_{leak} - I_{up} + I_{rel} \quad (133)$$
$$Ca_{srbufsr} = \frac{Ca_{sr} \times Buf_{sr}}{Ca_{sr} + K_{bufsr}} \quad (134)$$
$$\frac{dCa_{srtotal}}{dt} = \frac{V_C}{V_{SR}}(-I_{leak} + I_{up} - I_{rel}) \quad (135)$$

Sodium Dynamics

$$\frac{dNa_i}{dt} = -\frac{I_{Na} + I_{bNa} + 3I_{NaK} + 3I_{NaCa}}{V_C F} \quad (136)$$

Potassium Dynamics

$$\frac{dK_i}{dt} = -\frac{I_{K1} + I_{to} + I_{Kr} + I_{Ks} - 2I_{NaK} + I_{pK} + I_{stim} - I_{ax}}{V_C F} \quad (137)$$

Model Parameters

A. Channel conductances

G_{Na}: Maximal fast Na^+ current (I_{Na}) conductance =14.838 nS/pF
G_{CaL}: Maximal L-type Ca^{2+} current (I_{CaL}) conductance =1.75 × 10^{-4} cm^2 · μF^{-1}·s^{-1}

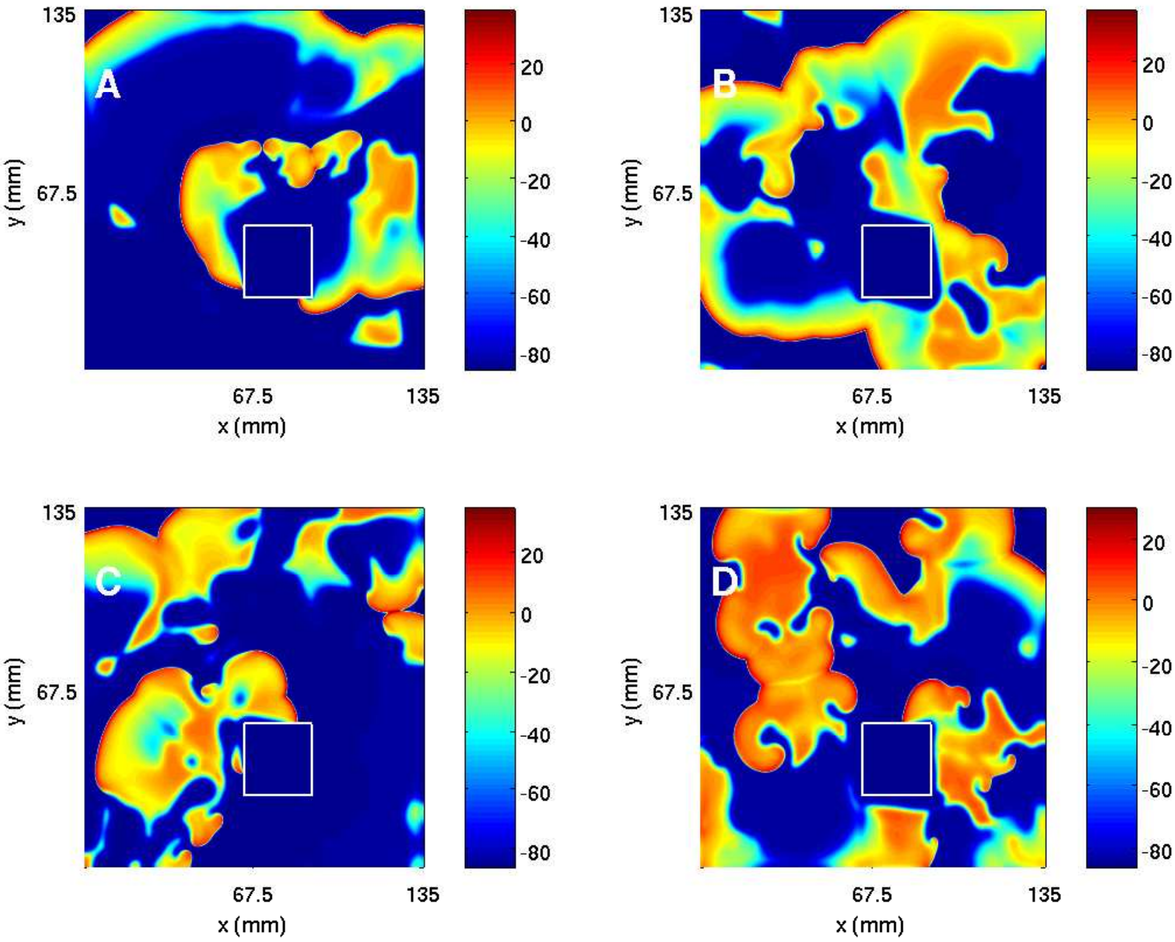

Figure 8: (Color online) The spiral-turbulence (ST) state in the TNNP model, with a square obstacle of side $l = 27$ mm at (63 mm, 27 mm) in a square simulation domain with $L = 135$ mm for $G_{CaL} = 0.000044$ (and all other parameters as in 2 C), illustrated via pseudocolor plots of V shown in (A), (B), (C), and (D) at $t = 0.8$, 1.6, 2.4, and 3.2 s, respectively.

G_{to}: Maximal transient outward current (I_{to}) conductance =0.294 nS/pF (for epicardial and M-cell), 0.073 nS/pF (for endocardial cell)
G_{Ks}: Maximal slow delayed rectifier current (I_{Ks}) conductance =0.245 nS/pF (for epicardial and endocardial), 0.062 nS/pF (for M-cell)
G_{Kr}: Maximal rapid delayed rectifier current (I_{Kr}) conductance =0.096 nS/pF
G_{K1}: Maximal inward rectifier K^+ current (I_{K1}) conductance =5.405 nS/pF
G_{pCa}: Maximal plateau Ca^{2+} current (I_{pCa}) conductance =0.825 nS/pF
G_{pK}: Maximal plateau K^+ current (I_{pK}) conductance =0.0146 nS/pF
G_{bNa}: Maximal background Na^+ current (I_{bNa}) conductance =0.00029 nS/pF
G_{bCa}: Maximal background Ca^{2+} current (I_{bCa}) conductance =0.000592 nS/pF

B. Ion concentration

Na_o: Extracellular Na^+ concentration =140 mM
Ca_o: Extracellular Ca^+ concentration =5.4 mM
K_o: Extracellular K^+ concentration =2 mM

C. Cell geometry (Volume)

V_C: Cytoplasmic volume =16404 μm^3
V_{SR}: Sarcoplasmic recticulum volume =1094 μm^3

D. Other parameters

p_{KNa}: Relative I_{Ks} permeability to Na^+ =0.03

k_{NaCa}: Maximal Na^+/Ca^{2+} exchanger current (I_{NaCa}) =1000 pA/pF
k_{mNai}: Na_i half-saturation constant for I_{NaCa} =87.5 mM
k_{mCa}: Ca_i half-saturation constant for I_{NaCa} =1.38 mM
k_{sat}: Saturation factor for I_{NaCa} =0.1
α: Factor enhancing outward nature of I_{NaCa} =2.5
γ: Voltage dependence parameter of I_{NaCa} =0.35

P_{NaK}: Maximal I_{NaK} =1.362 pA/pF
K_{mK}: K_o half-saturation constant of I_{NaK} =1 mM
K_{mNa}: Na_i half-saturation constant of I_{NaK} =40 mM

K_{pCa}: Ca_i half-saturation constant of I_{pCa} =0.0005 mM

V_{leak}: Maximal I_{leak} =0.00008 ms^{-1}

V_{maxup}: Maximal I_{up} =0.000425 mM/ms
K_{up}: Half-saturation constant of I_{up} = 0.00025 mM

a_{rel}: Maximal Ca_{SR}-dependent I_{rel} =0.016464 mM/ms
b_{rel}: Ca_{SR} half-saturation constant of I_{rel} =0.25 mM
c_{rel}: Maximal Ca_{SR}-independent I_{rel} =0.008232 mM/ms

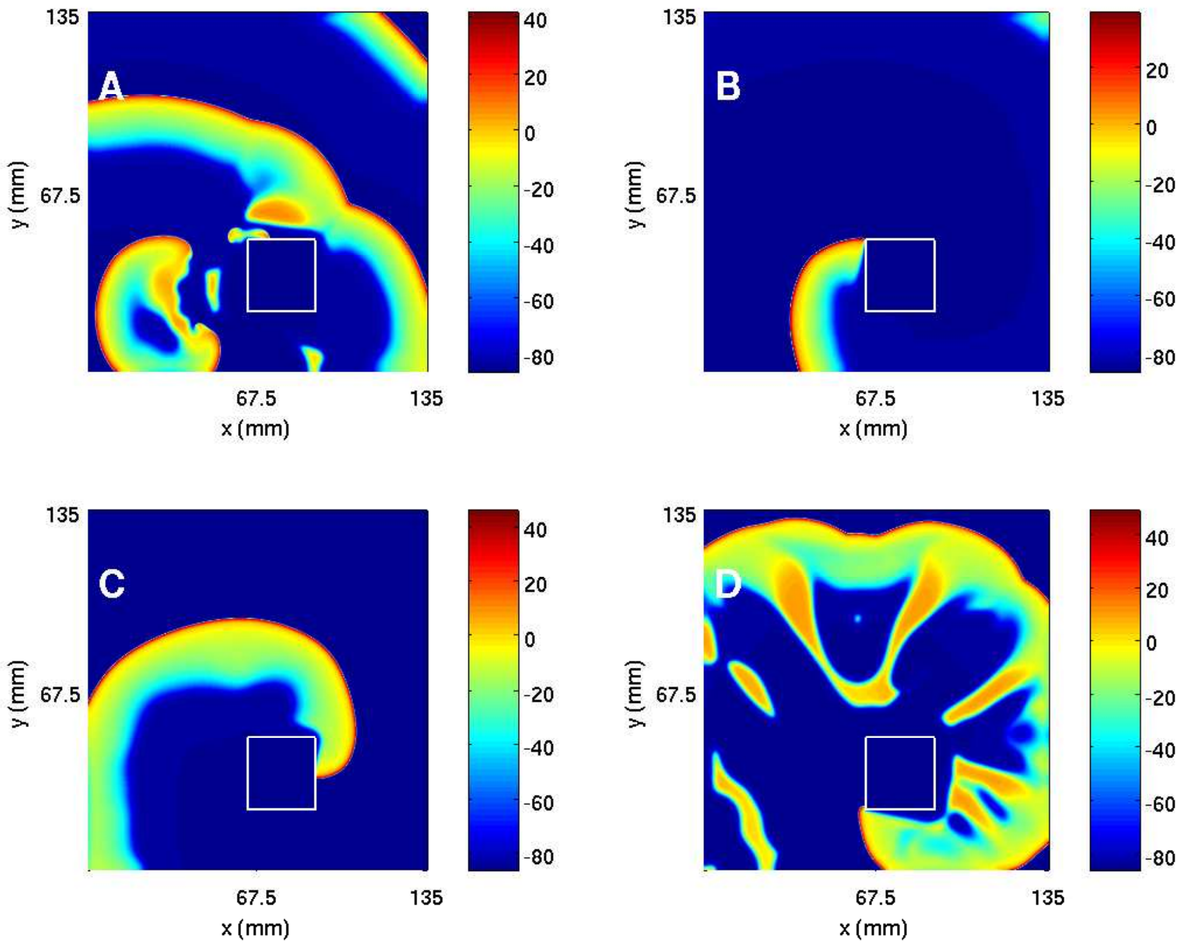

Figure 9: (Color online) Pseudocolor plots of V for the TNNP model showing a spiral wave attached to a square obstacle of side 27 mm placed at (63 mm, 22.5 mm), and other parameters as in Fig. 8, at (A) $t = 0.8$ s, (B) $t = 1.6$ s, (C) $t = 2.4$ s, and (D) $t = 3.2$ s.

Buf_c: Total cytoplasmic buffer concentration =0.15 mM
Buf_{sr}: Total sarcoplasmic recticulum buffer concentration =10 mM
K_{bufc}: Ca_i half-saturation constant for cytoplasmic buffer =0.001 mM
K_{bufsr}: Ca_{SR} half-saturation constant for sarcoplasmic recticulum buffer =0.3 mM

The initial gating variables and initial ion concentrations for this model are as follows:
m: Activation gate for $I_{Na} = 0$;
h: Fast inactivation gate for $I_{Na} = 0.75$;
j: Slow inactivation gate for $I_{Na} = 0.75$;

d: Activation gate for $I_{CaL} = 0$;
f: Inactivation gate for $I_{CaL} = 1$;
f_{Ca}: Intracellular calcium-dependent inactivation gate for $I_{CaL} = 1$;

r: Activation gate for $I_{to} = 0$;
s: Inactivation gate for $I_{to} = 1$;

x_s: Activation gate for $I_{Ks} = 0$;

x_{r1}: Activation gate for $I_{Kr} = 0$;
x_{r2}: Inactivation gate for $I_{Kr} = 1$;

g: Calcium-dependent inactivation gate for $I_{rel} = 1$;

Na_i: Intracellular Na^+ concentration =11.6 mM
Ca_i: Intracellular Ca^+ concentration =0.0002 mM
K_i: Intracellular K^+ concentration =138.3 mM
Ca_{sr}: Free Ca^{2+} concentration in the sarcoplasmic recticulum (SR) =0.2 mM

3 Spiral waves in homogeneous domains

A Spiral waves in the Panfilov model

To obtain a spiral wave in the Panfilov model we use [5] the following broken wave front at time $t = 0$: $g = 2$, for $0 \leq x \leq L$ and $0 \leq y \leq \frac{L}{2}$, and $g = 0$ elsewhere; and $V = 0$ everywhere except for $y = \frac{L}{2} + 1$ and $0 \leq x \leq \frac{L}{2}$, where $V = 0.09$. Spiral turbulence then sets in if $L \geq 128$ mm and we use the parameters given in Eq.(1), as shown, e.g., via pseudocolor plots of V and g in Ref. [5]. (In three dimensions, a similar broken wave yields scroll waves of the type that we will show below.) More pseudocolor plots of V are given in Figs. 1 A-C for different values of ϵ_1; at large values of ϵ_1 [e.g., $\epsilon_1 = 0.03$ in Figs. 1 A and D] we get a simple, periodically rotating spiral wave; at somewhat lower values [e.g., $\epsilon_1 = 0.02$ in Figs. 1 B and E] quasiperiodic behavior is obtained because the tip of the spiral meanders; and, at even lower values of ϵ_1, the end of the initial broken wave, which is not at the boundary, curls up and develops into a spiral wave that breaks up subsequently [Figs. 1 C and F]. This spiral-wave break up is associated with the onset of spatiotemporal chaos. Several positive Lyapunov exponents have been reported in this state. The number of positive exponents and the Kaplan-Yorke dimension D_{KY} increase with L, as shown in Ref. [6, 7] for this model. The time series for V, from a representative point in the simulation domain, also shows signatures of the underlying spral-wave states; in particular, the power spectra, Figs. 1 D-F, of such time series display, respectively, the characteristics of periodic, quasiperiodic, and chaotic behaviors; in particular, the last of these shows the broad-band background associated with chaos.

B Spiral waves in the LRI model

To initiate spiral waves in our simulations of the LRI model we begin by injecting a plane wave into the domain by applying a stimulation current of 150μA/cm^2 for 6 ms at the bottom boundary. As this plane wave moves towards the top boundary and 276 ms after the first stimulus, a second stimulus of 150μA/cm^2 is applied, for 1 ms, on the bottom boundary, till $x = 22.5$ mm, parallel to the first wave and in the region behind it. This is a broken wave in the wake of the initial plane wave. As the first wave moves further towards the top boundary, the second wave also moves upwards, and its free end curls up and eventually forms a spiral wave. We now change the conductivity to half its value (i.e., from 0.01 to 0.005) from 276 ms to 460 ms, so that the wave remains in the medium; we then bring the conductivity back to its original value. (Here and henceforth we do not specify the units for the conductivities, conductances, etc,; these are provided in Secs 2 A, B, and C for the LRI, RPB, and TNNP models, respectively.) The spiral wave now develops and starts breaking up by 500 ms.

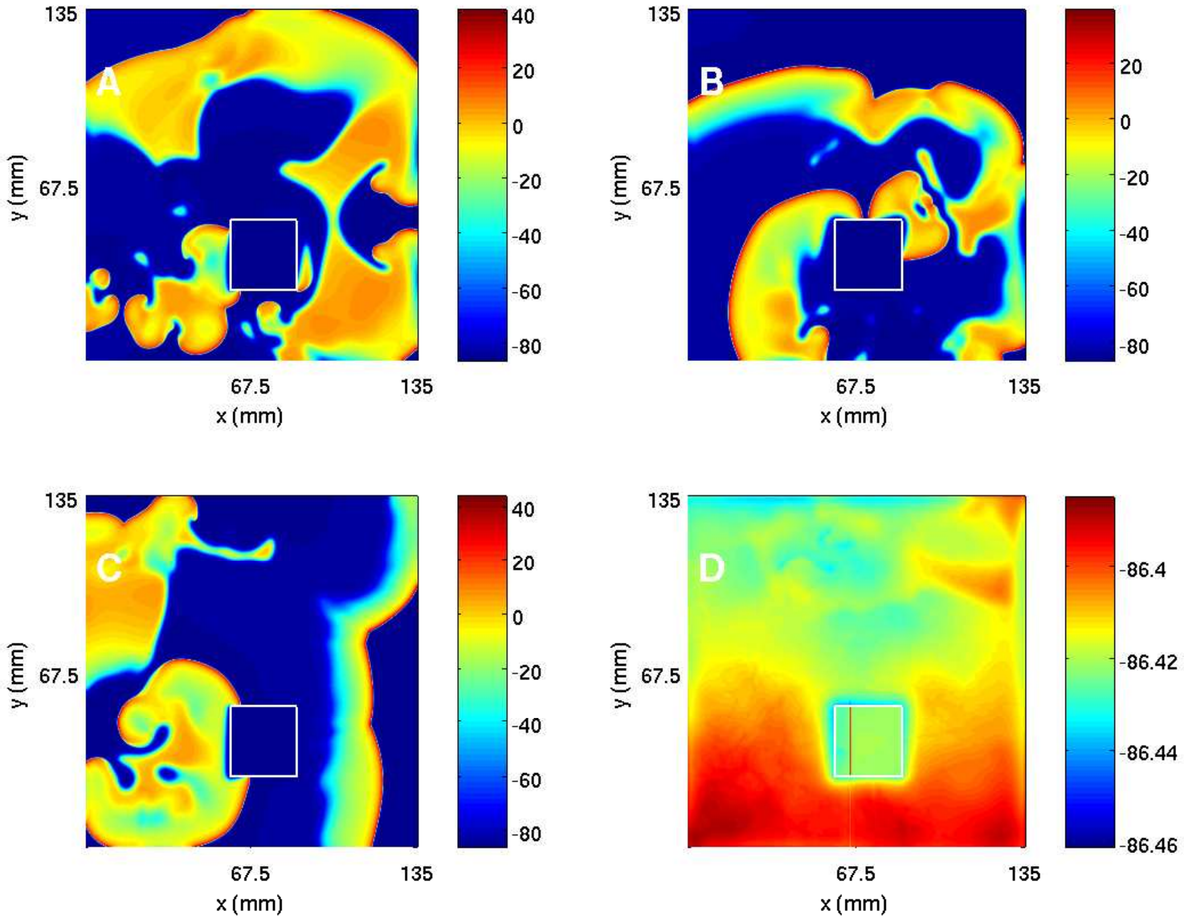

Figure 10: (Color online) The spiral wave moves away from the square simulation domain of side $L = 135$ mm for the TNNP model if a square obstacle of side $l = 27$ mm is placed at (58.5 mm, 27 mm), and other parameters as in Fig. 8, at (A) $t = 0.4$ s, (B) $t = 0.8$ s, (C) $t = 1.6$ s, and (D) $t = 2.4$ s.

A spiral wave prepared by such a procedure is shown in Fig. 2 via pseudocolor plots of V and the currents in the LRI model; this is the initial condition we use in our simulations of the LRI model both with and without inhomogeneities. At the moment the first plane wave is initiated the currents and gating variables are initialised as follows: the gating variables are set to their steady-state values, such as h_∞, and then the currents are calculated for these values of the gating variables

C Spiral waves in the RPB model

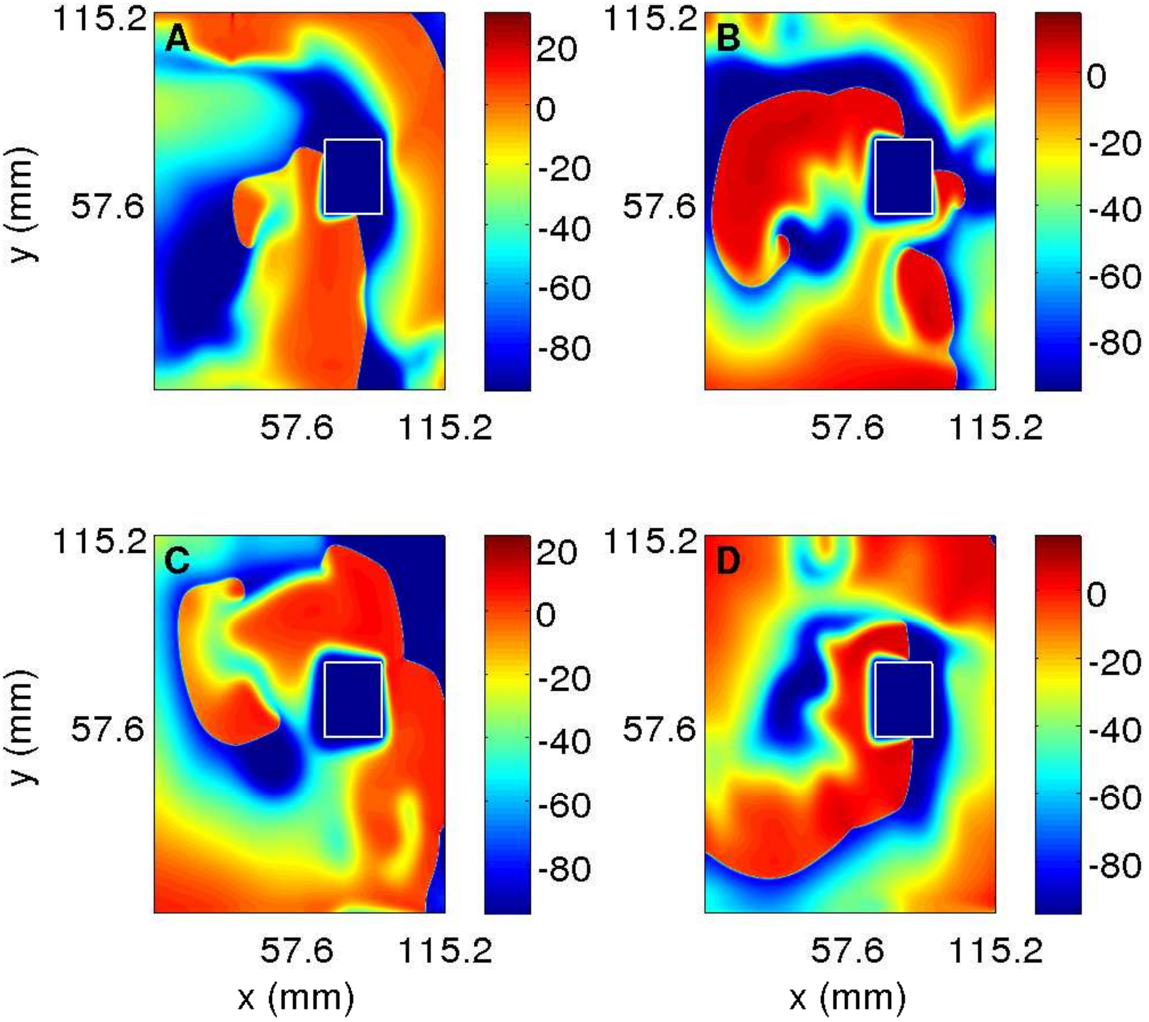

Figure 11: (Color online) Pseudocolor plots of V in the RPB model showing spiral break up in a simulation domain, with side $L = 11.52$ cm and a square obstacle of side 2.25 cm at (6.75 cm, 5.4 cm), at (A) $t = 0.4$ s, (B) $t = 1$ s, (C) $t = 1.6$ s, and (D) $t = 2$ s, respectively.

To initiate spiral waves in our simulations of the RPB model we begin by injecting a plane wave into the domain by applying a stimulation current of $100\mu\mathrm{A/cm}^2$ for 6 ms at the left boundary. As this plane wave moves towards the right boundary and 410 ms after the first stimulus, a second stimulus of $150\mu\mathrm{A/cm}^2$ is

applied, for 0.35 ms, on the bottom boundary, perpendicular to the first wave and in the region behind it. This leads to the formation of a narrow, linear region of electrical activation, behind the original plane wave, which evolves into a spiral wave by about 1230 ms. Given the conductivity constant we use, namely, 0.001 (see Sec. 2B), this spiral wave moves away from the simulation domain. To obtain a broken spiral wave that stays in the domain, we also have to change the conductivity to 0.0005 between 430 ms and 1230 ms; after this we reset the value of the conductivity to 0.001. At the moment the first plane wave is initiated the currents and gating variables are initialised as follows: the gating variables are set to their steady-state values, such as h_∞, and then the currents are calculated for these values of the gating variables and the resting value of V. The procedure outlined above yields rich spatiotemporal dynamics that is shown via the sequence of pseudocolor plots for the transmembrane potential V and the currents I_{Na}, I_t, at $t = 2000$ ms; the states shown in these figures are used as initial conditions for our subsequent simulations of the RPB model both with and without inhomogeneities. In the absence of inhomogeneities such an initial condition leads to broken spiral waves as shown in the illustrative pseudocolor plot of V in Fig. 3.

D Spiral waves in the TNNP model

We obtain spiral waves in the TNNP model by injecting a plane wave into the domain via a stimulation current of $150\mu\text{A/cm}^2$ for 2 ms at the left boundary. As this plane wave moves towards the right boundary (Fig. 4 A) and 270 ms after the first stimulus, we apply a second stimulus of $450\mu\text{A/cm}^2$ along a line behind this wave but parallel to it ($x = 290$, $1 \leq y \leq 250$) for 10 ms. As the first wave moves further towards the right, the free end of the new stimulus is able to move into the area behind the first wave; a hook-like proto spiral appears at this free end (Fig. 4 B). We now change the conductivity D from 0.00154 cm^2/ms to 0.000385 cm^2/ms between 304 ms to 524 ms; this yields the fully developed spiral wave of Fig. 4 D; we then reset the conductivity to its original value after 524 ms. At the moment the first plane wave is initiated the currents and gating variables are initialised as follows: the gating variables are given in Sec. 2 C and the currents are calculated for these values of the gating variables and the resting value of V, which is -86.2 mV.

Given the parameters that we have used so far for the TNNP model we get a single spiral wave by the procedure described in the main paper. We have been able to obtain spiral-wave breakup in this model only by changing some conductances. For instance, if we change G_{CaL} to one fourth of its maximal value (i.e., we set $G_{CaL} = 0.000044$), but hold all other parameters at the values specified in Sec 2C, we get the spiral turbulence shown via the pseudocolor plots of Fig. 5; we can also get similar spiral turbulence if, instead of changing G_{CaL}, we set $G_{pCa} = 3.825$.

E A comparison of spiral waves in different models

From our studies of spiral waves in the four models described above, we see that many qualitative features of spiral-wave dynamics are the same in the Panfilov, LRI, RPB, and TNNP models. However, there are important differences, some qualitative and the others quantitative. For example, the Panfilov model cannot address directly any questions regarding currents in ion channels since it does not follow their evolution but only considers one slow recovery variable g. The LRI, RPB, and TNNP models do give spatiotemporal information for several ion-channel currents as shown, for representative cases, in Figs. 2, and 6. At any given time, the qualitative form of the spatial organization of these currents can be surmised from the spatial distribution of the transmembrane potential V and the dependence of these currents on V at the level of a single cell. To illustrate this we show for the LRI model, in Fig. 7, the action potential and the temporal evolution of the currents from a single-cell simulation (i.e., without the diffusion term in the LRI equations [5]). For instance, at the single-cell level, the sodium current I_{Na} is substantial only at the beginning of the action potential (Fig. 7); the most prominent parts of the spiral waves in pseudocolor plots of V appear in regions of the simulation domain where, locally, V assumes a value close to the sharp peak in the single-cell action potential; thus pseudocolor plots of the sodium current I_{Na} (Fig. 2) show significant structure only in narrow strips that follow closely the prominent parts of the spiral waves in pseudocolor plots of V (Fig. 2). By contrast, the slow inward calcium current I_{si} is significant in the plateau regime of the action potential (Fig. 7); thus pseudocolor plots of I_{si} (Fig. 2) show structure in most parts of the

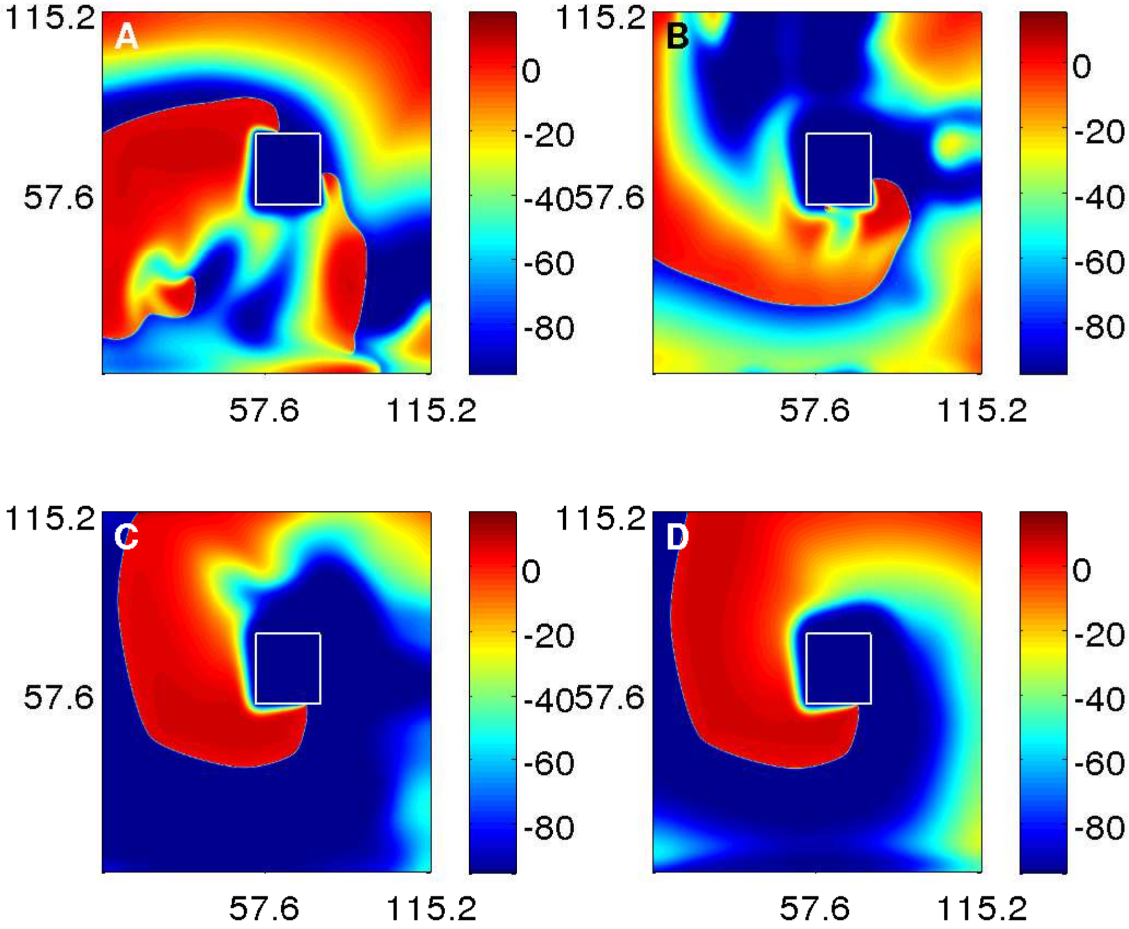

Figure 12: (Color online) Pseudocolor plots of V in the RPB model showing a spiral wave anchoring to the obstacle in a square simulation domain, with side $L = 11.52$ cm and a square obstacle of side 2.25 cm at (5.4 cm, 5.4 cm), at (A) $t = 0.4$ s, (B) $t = 1$ s, (C) $t = 1.6$ s, and (D) $t = 2$ s, respectively.

simulation domain, but the underlying spiral wave in V is still discernible. The potassium current I_{K1} is substantial in the plateau and repolarization regimes of the action potential (Fig. 7), so we should expect pseudocolor plots of I_{K1} to have significant structure not only along prominent parts of the spiral wave in V but also along the back of this wave, where repolarization occurs; this expectation is borne out as can be seen from Fig. 2. The leak current (I_b) is proportional to the voltage (Fig. 7), so the pseudocolor plot of I_b follows that of V (Fig. 2).

4 Conduction inhomogeneities

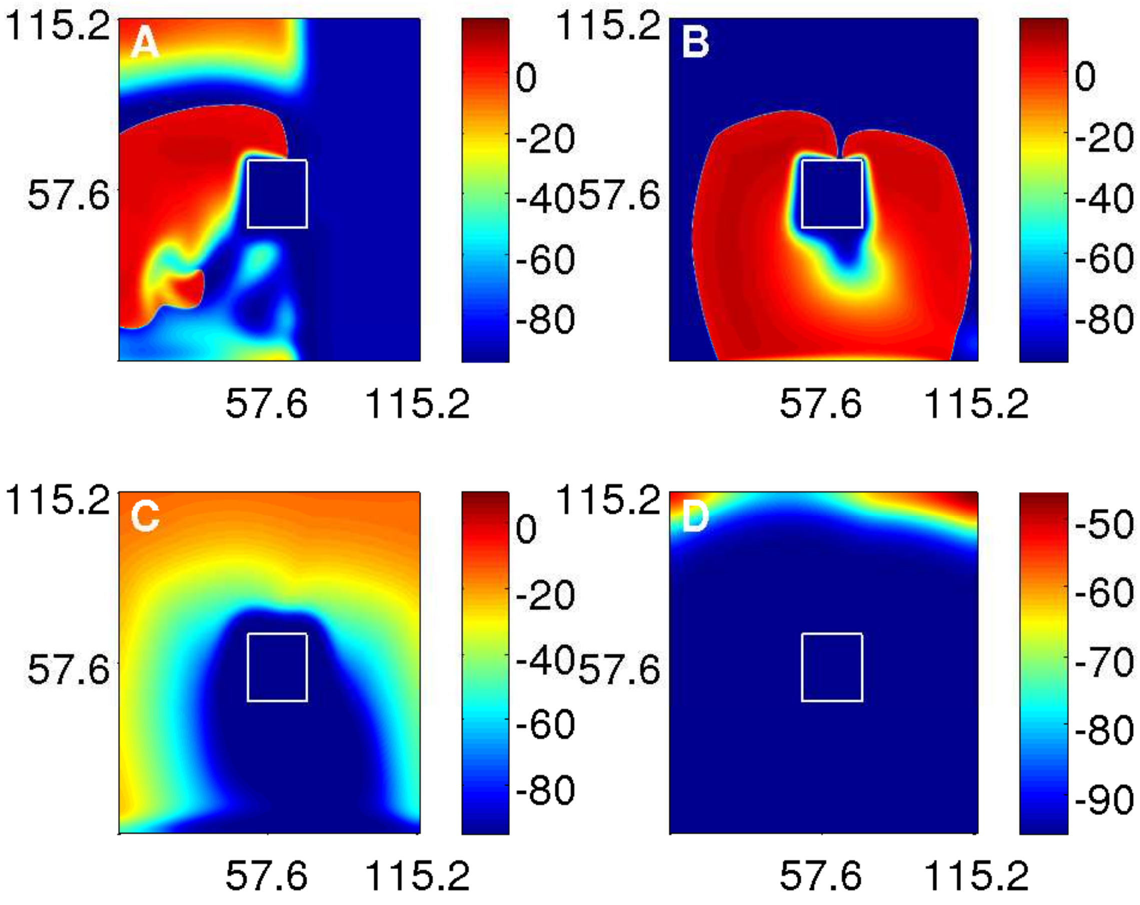

Figure 13: (Color online) Pseudocolor plots of V in the RPB model showing a spiral wave moving away from a square simulation domain, with side $L = 11.52$ cm and a square obstacle of side 2.25 cm at at (4.95 cm, 4.5 cm), at (A) $t = 0.4$ s, (B) $t = 1$ s, (C) $t = 1.6$ s, and (D) $t = 2$ s, respectively.

In the main text we have investigated the effect of an obstacle on a single spiral wave in the TNNP model. We now show that an obstacle affects a state with spiral turbulence (in the absence of the obstacle) in qualitatively the same way in which it influences a single spiral wave, i.e., the final state of the system depends sensitively on the position of the obstacle. Recall that, in the homogeneous TNNP model (Sec. 3D), we obtained spiral turbulence by setting $G_{CaL} = 0.000044$ (or by using $G_{pCa} = 3.825$). In our studies with

obstacles we use $G_{CaL} = 0.000044$ and all other parameters as in Sec. 2C. We now insert a square obstacle of side $l = 27$ mm into a square simulation domain of side $L = 135$ mm. When the obstacle is at (63 mm, 27 mm), the system remains in the ST state (Fig. 8); if it is at (63 mm, 22.5 mm), the system evolves from the ST to the RS state with one rotating spiral wave anchored at the obstacle (Fig. 9). and with the obstacle at (58.5 mm, 27 mm) spiral waves get eliminated completely and we get a quiescent state (Fig. 10).

We have also carried out systematic studies of spiral-wave dynamics in the presence of a circular obstacle in the TNNP model; in this case too the results are qualitatively similar to those that we obtain with a square obstacle. We use a circular obstacle of radius 16.875 mm in the 135 mm square simulation domain with conductivity constant $D = 0.00154$ cm^2/ms; we introduce a conduction inhomogeneity by reducing the conductivity constant inside the inhomogeneity to a very small value, namely, 10^{-6} cm^2/ms. (Since the conductivity constant is not identically zero inside the inhomogeneity, we do not need to impose Neumann boundary condition on the circular boundary.) We find that, when the center of this circular obstacle is at (67.5 mm, 33.75 mm), the system evolves into the state ST as shown in Fig. 14; if it is at (56.25 mm, 33.75 mm), a single rotating spiral (RS) gets attached to the obstacle (Fig. 15); and, with the obstacle at (67.5 mm, 22.5 mm), spiral waves get eliminated completely and we get a quiescent state Q (Fig. 16).

The sensitive dependence of spiral-wave dynamics on the position of an obstacle also occurs in the RPB model. For example, in Fig. 11 we show representative pseudocolor plots of the transmembrane potential V for the RPB model in a square domain of side $L = 115.2$ mm with a square obstacle of side $l = 22.5$ mm; in all cases we start with the initial condition described above. If this obstacle is placed with its left bottom corner at (6.75 mm, 5.4 mm) in the domain, then the spiral wave breaks up and the system remains in the state ST. But when the same obstacle is placed at (5.4 mm, 5.4 mm), the spiral wave gets anchored to the obstacle (state RS), as illustrated in Fig. 12. If we move the left bottom corner of the obstacle to (4.95 mm, 4.5 mm), as in Fig. 13, then all spiral waves move away from the medium and leave it in the quiescent state Q.

The stability diagrams (see the main paper) are shown, respectively, in Figs. 17 and 18 for LRI and RPB models with the simulation domains divided into small square regions of side $l_p = 4.5$ mm The color of each small square indicates the final state of the system when the position of the lower-left corner of the obstacle coincides with that of the small square: red, blue, and green indicate, respectively, ST, RS, and Q. In both these simulations we use square obstacles with sides 22.5 mm; the square simulation domains have sides $L = 90$ mm and $L = 115.2$ mm for the LRI and RPB models, respectively. For the TNNP model we present the partial stability diagram for $G_{pCa} = 3.825$ in Fig. 19, and square obstacle of sides 22.5 mm; all other parameters are as specified in the figure captions and in Sec. 2 C. These partial stability diagrams suggest that the boundaries between ST, RS, and Q states in the LRI and RPB models are as complicated as in the simple Panfilov model. Thus, as we have stated earlier, spiral-wave dynamics in all these models depends very sensitively on the position of a conduction inhomogeneity.

5 Ionic Inhomogeneities

To study ionic inhomogeneities (Sec. on "Results" of the main paper) We now introduce a square inhomogeneity in ϵ_1 in the Panfilov model (all other parameters are uniform over the simulation domain): ϵ_1 is assigned the value ϵ_1^{in} inside a square region; and outside this square it has the value ϵ_1^{out}. Different choices of ϵ_1^{in} and ϵ_1^{out} lead to interesting spiral-wave dynamics. For example, with a square patch of side 40 mm, $\epsilon_1^{in} = 0.02$ and $\epsilon_1^{out} = 0.01$, we obtain spatiotemporal chaos for most positions of this inhomogeneity; but for certain critical positions of this inhomogeneity all spiral waves are completely eliminated; e.g., when the inhomogeneity is at ($x = 130$ mm, $y = 80$ mm), spiral waves move towards the boundaries of the simulation domain where they are eventually absorbed (Fig. 20). For yet other positions spatiotemporal chaos is obtained outside the inhomogeneity but inside it the spiral wave shows a quasiperiodic temporal evolution as illustrated in Fig. 21.

If, instead, $\epsilon_1^{in} = 0.01$ and $\epsilon_1^{out} = 0.02$ or 0.03, spiral-wave break up occurs inside the inhomogeneity but it coexists with unbroken periodically rotating spiral waves outside it (Fig. 22), as noted previously by Xie *et al.* [8].

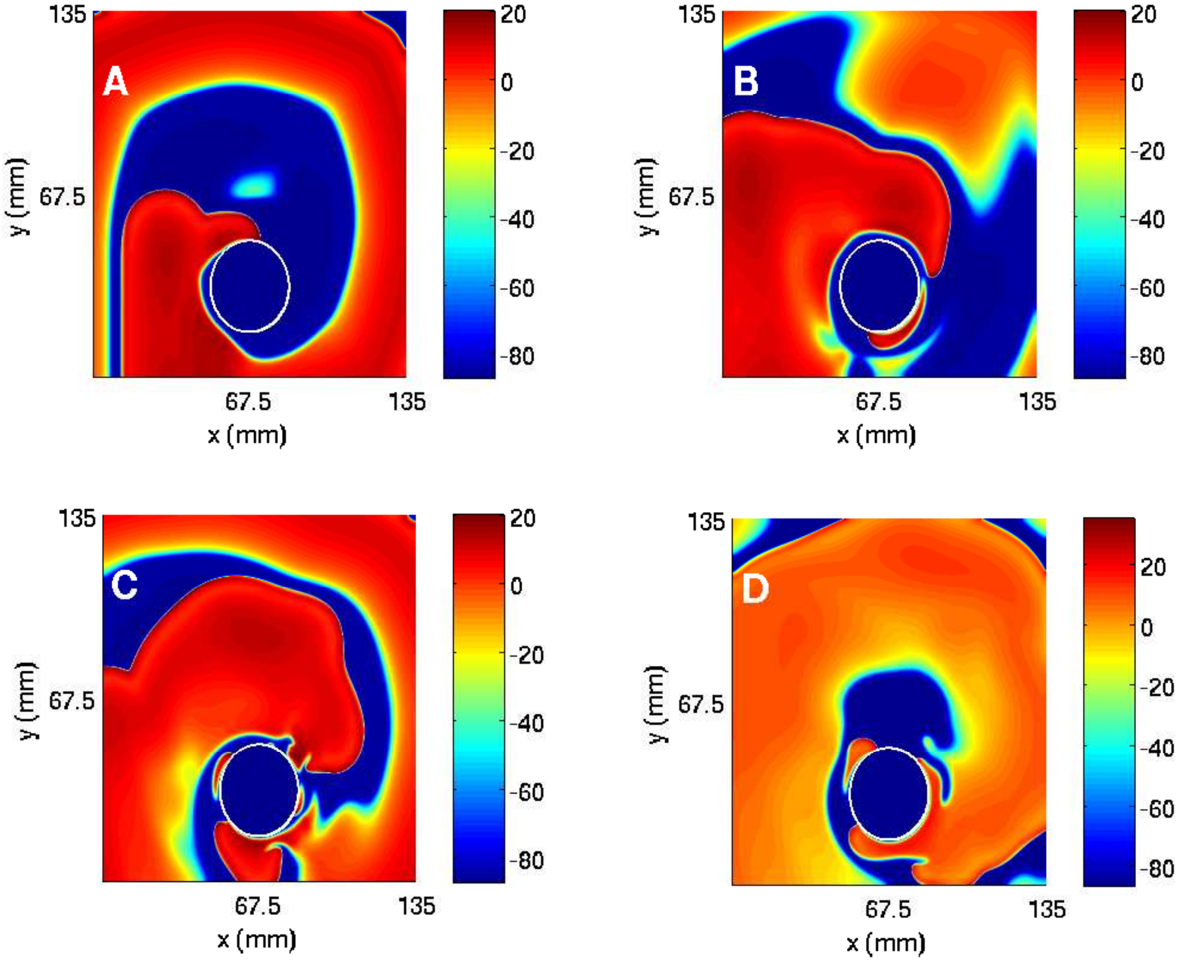

Figure 14: (Color online) The spiral-turbulence (ST) state in the TNNP model, with a circular obstacle of radius $r = 16.875$ mm at (67.5 mm, 33.75 mm) in a square simulation domain with $L = 135$ mm, illustrated via pseudocolor plots of V shown in (A), (B), (C), and (D) at $t = 200$, 400, 800, and 1160 ms, respectively.

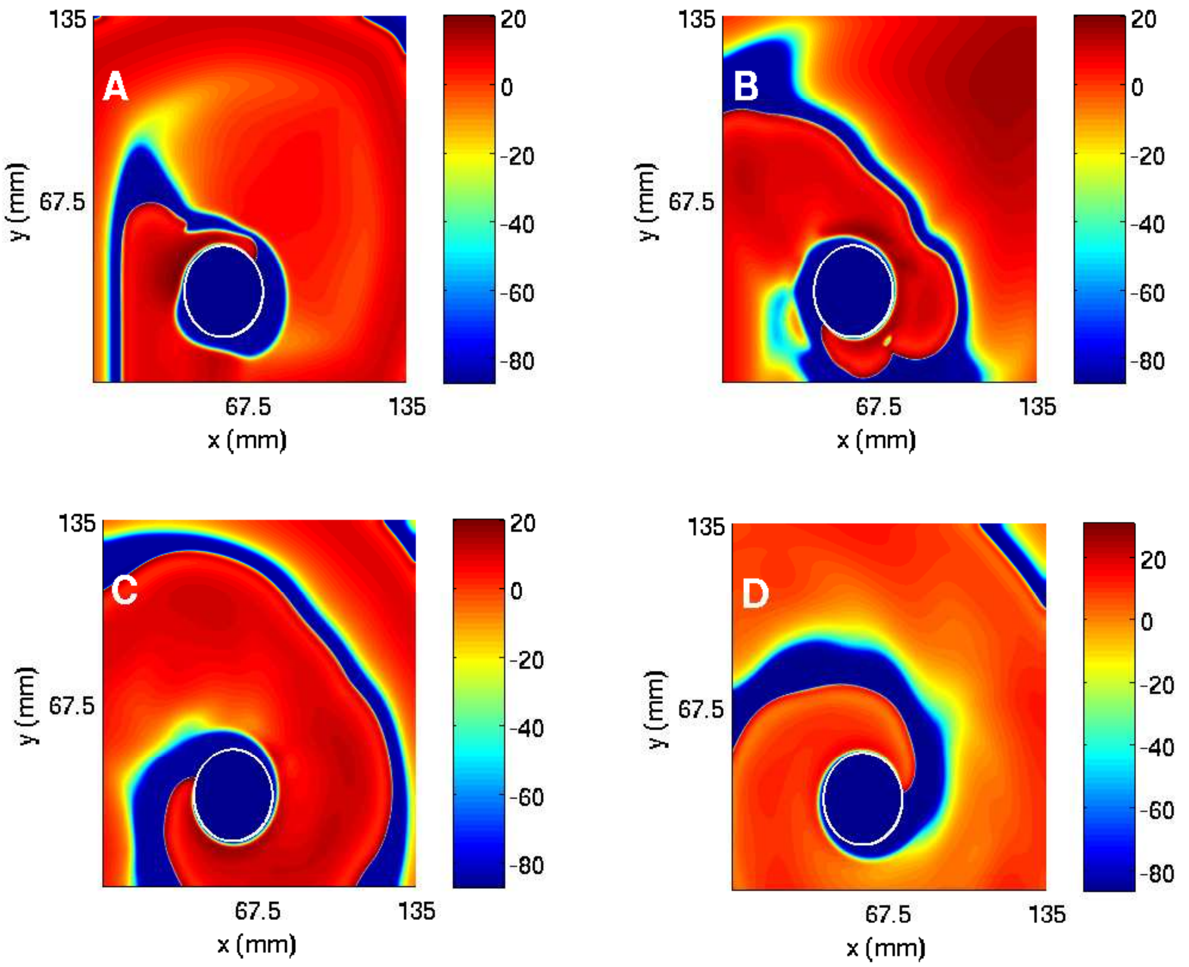

Figure 15: (Color online) Pseudocolor plots of V for the TNNP model showing a spiral wave attached to a circular obstacle of radius 16.875 mm placed at (56.25 mm, 33.75 mm), at (A) $t = 200$ ms, (B) $t = 400$ ms, (C) $t = 800$ ms, and (D) $t = 1160$ ms.

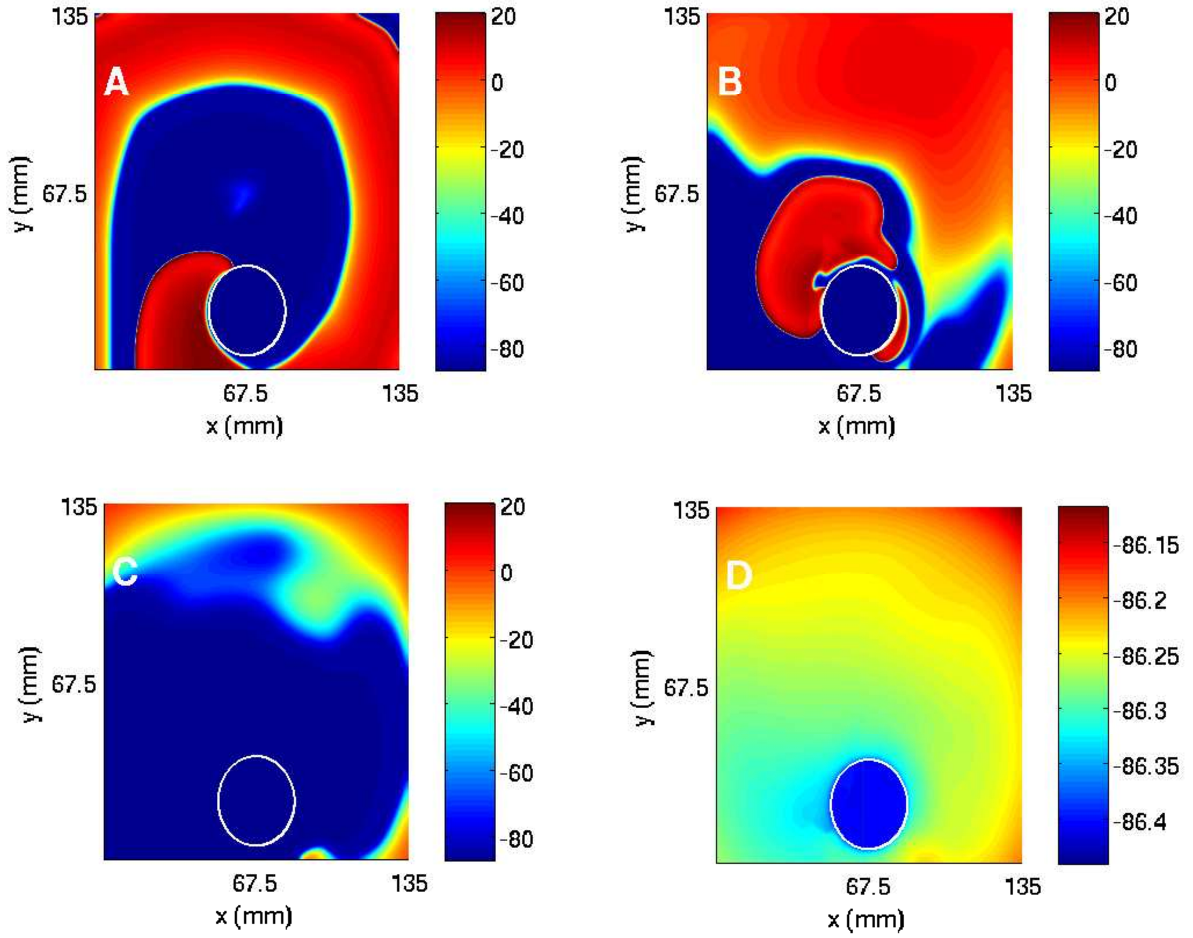

Figure 16: (Color online) The spiral wave moves away from the square simulation domain of side $L = 135$ mm for the TNNP model if a circular obstacle of radius $r = 16.875$ mm is placed at (67.5 mm, 22.5 mm), at (A) $t = 200$ ms, (B) $t = 400$ ms, (C) $t = 800$ ms, and (D) $t = 1160$ ms.

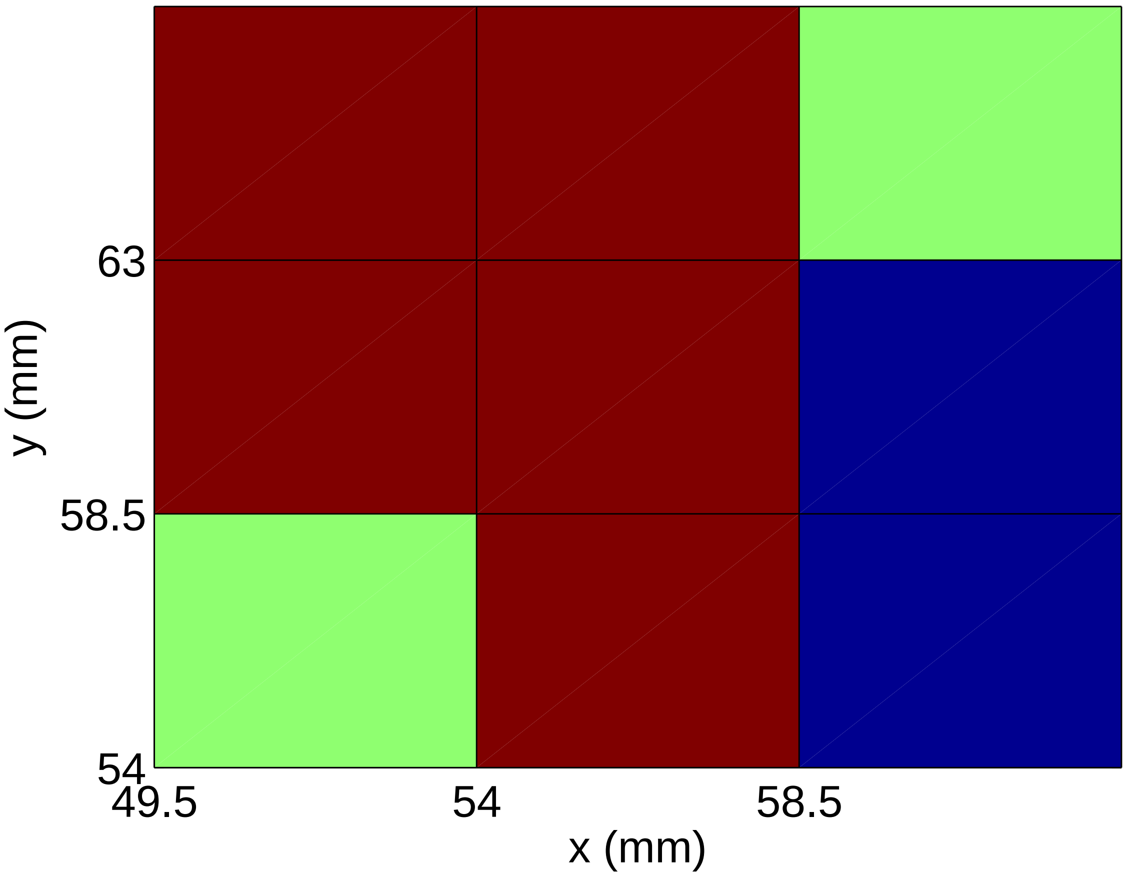

Figure 17: (Color online) Detail of the stability diagram for the LRI model with the initial condition described in the text: A square obstacle of side 22.5 mm is placed at different positions in a square simulation domain with side $L = 90$ mm. For each one of these positions of the obstacle we determine the final state of the system; the colors of the small squares, of side $l_p = 4.5$ mm, indicate the final state of the system when the position of the bottom-left corner of the obstacle coincides with that of the small square (red, blue, and green denote ST, RS, and Q, respectively).

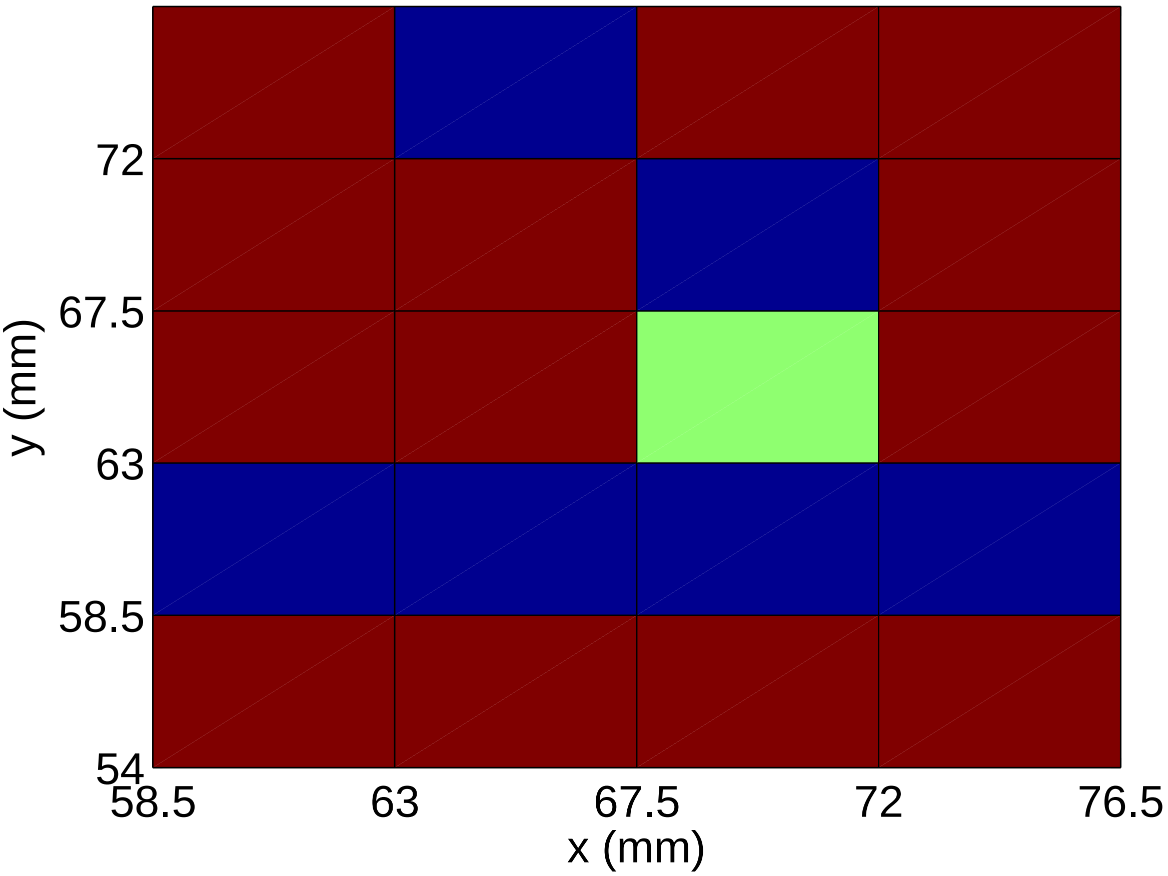

Figure 18: (Color online) Detail of the stability diagram for the RPB model with the initial condition described in the text: A square obstacle of side 22.5 mm is placed at different positions in a square simulation domain with side $L = 115.2$ mm. For each one of these positions of the obstacle we determine the final state of the system; the colors of the small squares, of side $l_p = 4.5$ mm, indicate the final state of the system when the position of the bottom-left corner of the obstacle coincides with that of the small square (red, blue, and green denote ST, RS, and Q, respectively).

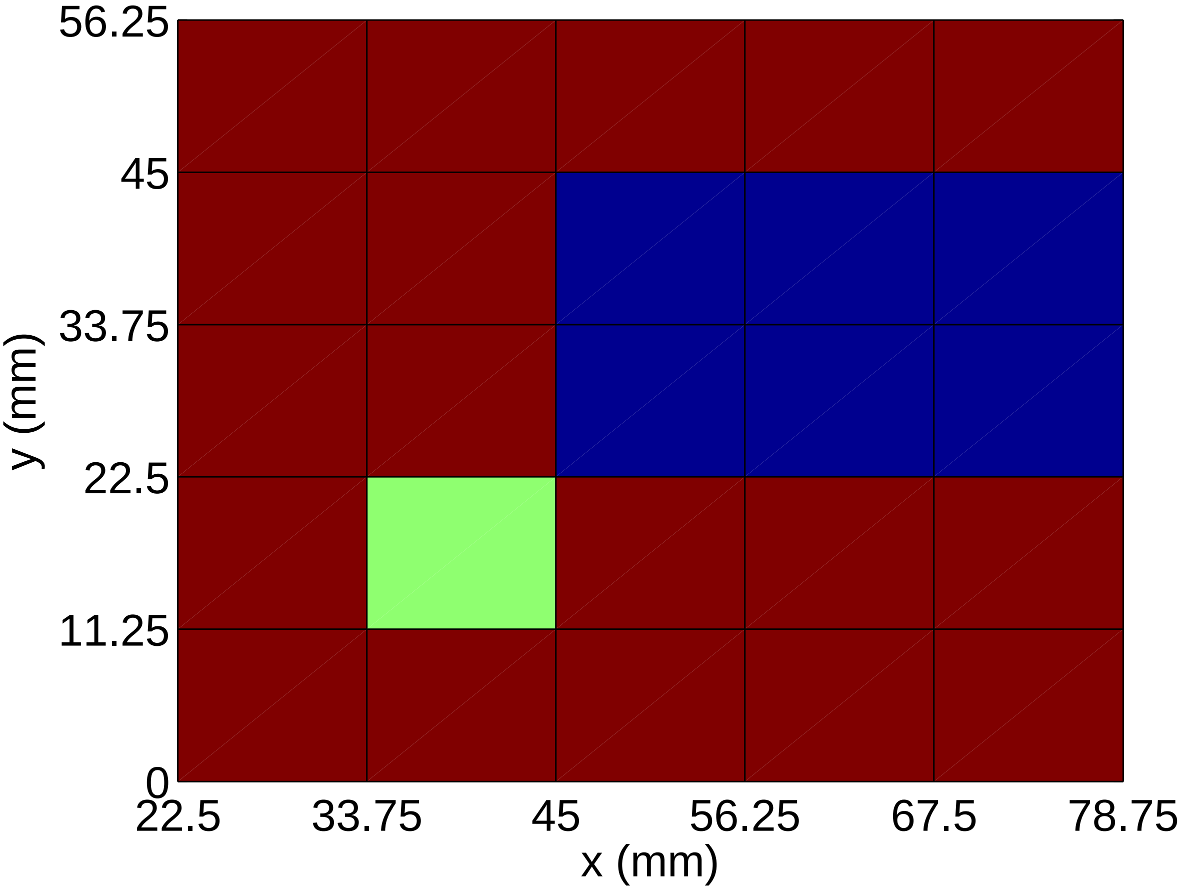

Figure 19: (Color online) Detail of the stability diagram for the TNNP model for $G_{pCa} = 3.825$ and all other parameters as in the 2 C and with the initial condition described in the text: A square obstacle of side 22.5 mm is placed at different positions in a square simulation domain with side $L = 135$ mm. For each one of these positions of the obstacle we determine the final state of the system; the colors of the small squares, of side $l_p = 11.25$ mm, indicate the final state of the system when the position of the bottom-left corner of the obstacle coincides with that of the small square (red, blue, and green denote ST, RS, and Q, respectively).

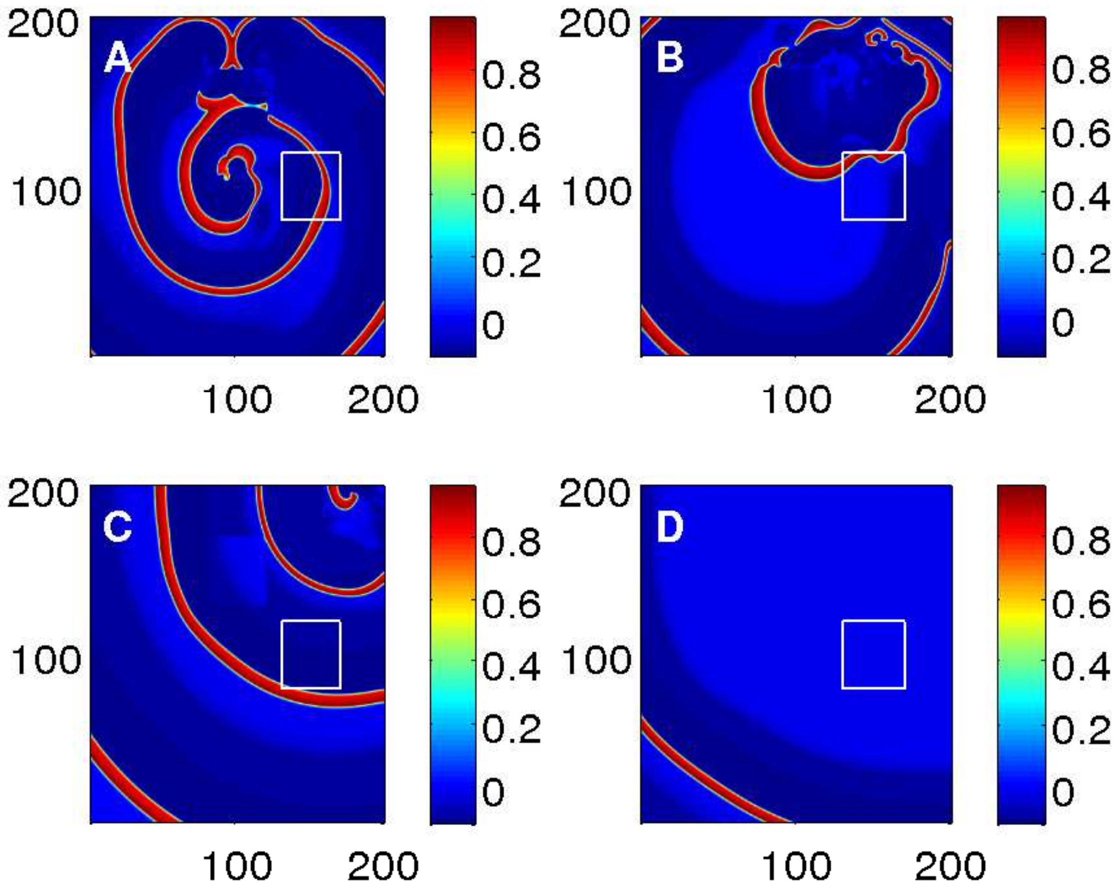

Figure 20: (Color online) Pseudocolor plots of V illustrating the effect of an inhomogeneity in the Panfilov model: With a square inhomogeneity of side 40 mm, $\epsilon_1^{in} = 0.02$ and $\epsilon_1^{out} = 0.01$, at ($x = 130$ mm, $y = 80$ mm) spiral waves move towards the boundaries of the simulation domain where they are eventually absorbed as shown in (A) at $t = 550$ ms, (B) $t = 1100$ ms, (C) $t = 1650$ ms, and (D) at 2200 ms.

We now present simulations of the homogeneous LRI model in a square domain of side 90 mm, with all parameters except G_{si} as in Sec. 2 A and the initial condition described above. In Fig. 26 we show pseudocolor plots of V, which are obtained after 100 ms, and the associated power spectra that we calculate from a time series of V from a representative point; we show plots for $G_{si} = 0.02, 0.035, 0.05$, and 0.07, (units in Sec. 2 A) [2]. (For single-cell action potentials for these values of G_{si}, see Fig. 23.) Data for the time series in Fig. 26 are collected for 262144 iterations, i.e., 2621.44 ms. From this figure we see that, as G_{si} increases, a simple rotating spiral evolves to a state with broken spiral waves and spatiotemporal chaos; the power spectra obtained from the time series of V, taken from a representative point in the simulation domain, also mirror this evolution: At low values of G_{si} the power spectra show sharp peaks that can be indexed with one fundamental frequency and its multiples, a mark of periodic temporal behavior; as G_{si} increases this indexing requires independent, incommensurate frequencies and their multiples (i.e., the temporal evolution is quasiperiodic); and finally at large values of G_{si} we obtain spectra with a significant broad-band background that is a signature of chaos. Figure 26 is the LRI-model analog of Fig. 1 for the Panfilov model.

We now investigate the effects of ionic inhomogeneities in the LRI model in some illustrative cases by introducing inhomogeneities in G_{si} and the potassium conductance G_k. Consider first a simulation domain with $G_{si} = G_{si}^{out} = 0.07$ everywhere except in a square patch with side 18 mm where $G_{si} = G_{si}^{in} = 0.02$ (we will refer to this as a G_{si} inhomogeneity); all other parameters for this LRI simulation are as in Sec. 2 A. With the initial condition given above, we study spiral-wave dynamics in this system with the square G_{si} inhomogeneity placed in different positions. For most positions we find a state with spiral turbulence; but for certain positions, e.g., when the inhomogeneity is at (49.5 mm, 45.0 mm) all spiral waves move towards the boundary where they are absorbed. As in the case of conduction obstacles we find that G_{si} inhomogeneities in the LRI model can affect spiral break up significantly; and the final state of the system depends sensitively on the position of the ionic inhomogeneity. This is illustrated in Figs. 24 A and B for a square G_{si} inhomogeneity of side 2.25 cm with $G_{si}^{in} = 0.07$, $G_{si}^{out} = 0.02$, and a simulation domain of side 90 mm. If this inhomogeneity is at (63 mm, 63 mm), we get a spiral wave that rotates periodically outside the inhomogeneity; and wave-break occurs inside the inhomogeneity. Plots of the interbeat interval (IBI) versus the beat number n from representative points outside and inside the inhomogeneity are shown in Figs. 24 C and D, respectively; the corresponding power spectra are given in Figs. 24 E and F, respectively.

Furthermore, a spiral wave can get anchored to a G_{si} inhomogeneity in the LRI model. For example, if we place the inhomogeneity used in Fig. 24 at (49.5 mm, 58.5 mm), the spiral gets anchored to it as depicted in Figs. 25 A and B via pseudocolor plots of V at 0.8 s and 1.2 s, respectively. Plots of the interbeat interval (IBI) versus the beat number n from representative points outside and inside the inhomogeneity are shown in Figs. 25 C and D, respectively; the corresponding power spectra are given in Figs. 25 E and F, respectively. Since $G_{si}^{in} = 0.07$, we might expect chaotic behavior inside this inhomogeneity by comparison with the homogeneous simulation of Fig. 26 D; the IBI plot of Fig. 25 C and the power spectrum of Fig. 25 E, which has a broad-band background below a few major peaks, confirm this expectation. Similarly, since $G_{si}^{out} = 0.02$, we might expect periodic behavior outside this inhomogeneity; the IBI plot of Fig. 25 D and the power spectrum of Fig. 25 F show that this is nearly the case: the rotation of the anchored spiral turns out to be quasiperiodic (the major peaks in the power spectrum can be indexed as $n_1\omega_1 + n_2\omega_2$, with n_1 and n_2 integers and the frequencies $\omega_1 \simeq 6.5$ Hz and $\omega_2 \simeq 3.4$ Hz, which are not related to each other by simple rational numbers). As in the case of an ϵ_1 inhomogeneity in the Panfilov model, a spiral wave can enter the region spanned by a G_{si} inhomogeneity, so the spatiotemporal patterns are richer than their analogs for a conduction inhomogeneity. We have found similar spatiotemporal behaviors for spiral-wave dynamics in the LRI model with inhomogeneities in G_k, but, given this similarity, we do not discuss these in detail here.

We can obtain stability diagrams for G_{si} inhomogeneities just as we had for the case of conduction inhomogeneities (cf. Figs. 17 and 18). A partial stability diagram for a G_{si} inhomogeneity of side 2.25 cm in a simulation domain of side 9 cm is given in Fig. 27; in this study we change the position of the inhomogeneity in steps of $l_p = 0.45$ cm. The final state of the system is indicated by a color code: red, blue, and green squares denote, respectively, ST, RS, and Q states; in these stability diagrams we do not distinguish between quasiperiodic and periodic behaviors. As in the case of conduction inhomogeneities, we see that the final state of the system depends very sensitively on the position of the inhomogeneity.

Similarly we study ionic inhomogeneities in the RPB model by introducing inhomogeneities in the calcium conductance G_{si}. Here too we find that such G_{si} inhomogeneities can lead to spiral-wave break up, anchoring of the wave at the inhomogeneity, or elimination of all waves from the medium. If we use a square simulation domain of side 11.52 cm with no inhomogeneity, $G_{si} = 0.064$, and the initial condition described above, we get spiral-wave turbulence. Now if we insert a square G_{si} inhomogeneity, in most cases spiral break up and turbulence continue; but, for certain positions of the inhomogeneity, we see different behaviors: For example, when $G_{si}^{out} = 0.064$ and we set $G_{si}^{in} = 0.02$ in a square region of side 22.5 mm at (58.5 mm, 67.5 mm), spiral waves move away from the medium and leave it in the quiescent state Q (Fig. 28); if instead $G_{si}^{out} = 0.02$ and $G_{si}^{in} = 0.064$ in a square region of the same size but placed at (58.5 mm, 58.5 mm), we obtain a single rotating spiral (Fig. 29).

To investigate ionic inhomogeneities in the TNNP model we consider the calcium conductance G_{CaL} that governs the ionic current I_{CaL} (see Sec. on "Results" of the main paper and Sec. 2 C). Figure 30 shows how the action potential (AP) is modified at the single-cell level as we lower G_{CaL} from 0.000175, the maximal

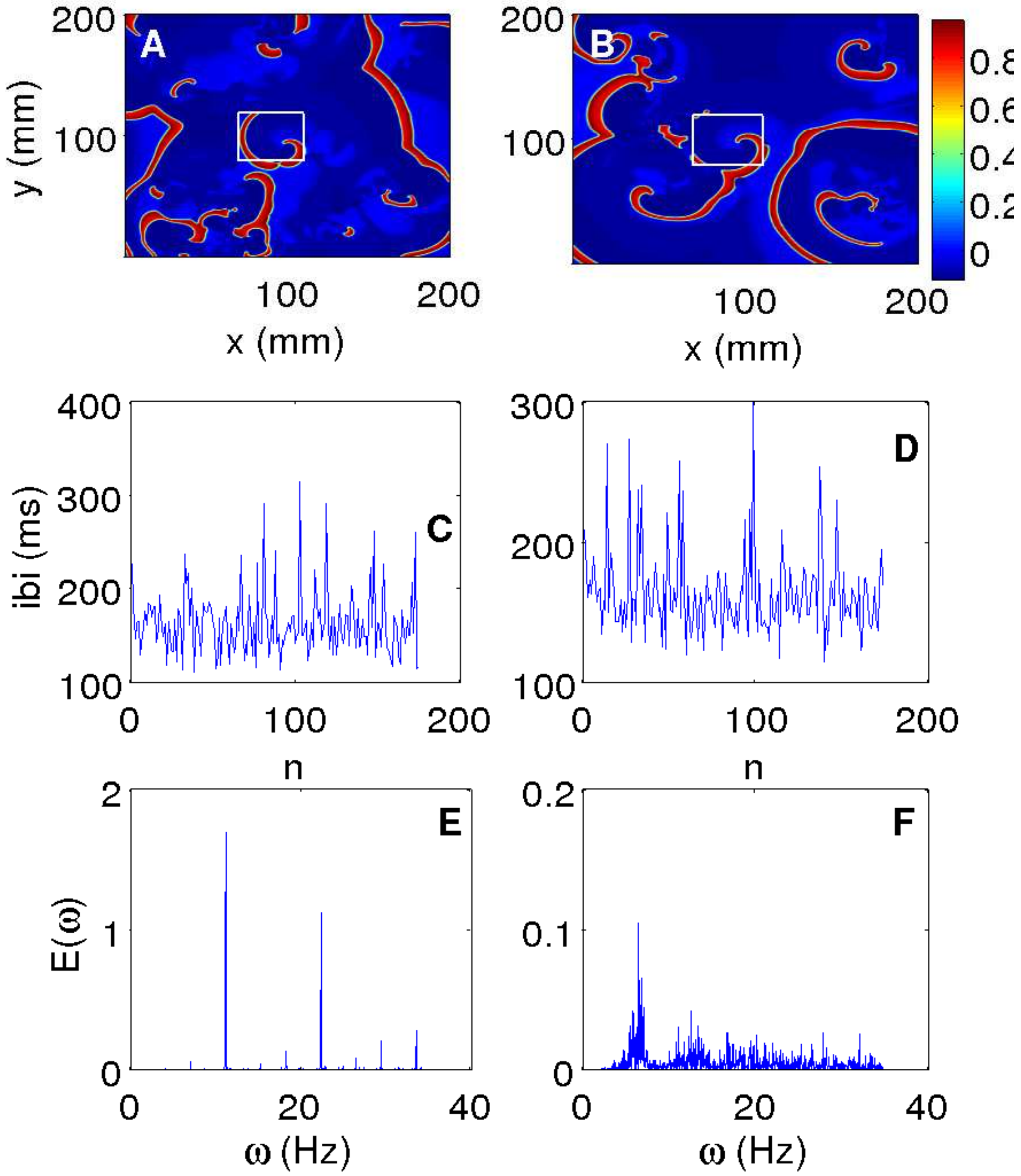

Figure 21: (Color online) Inhomogeneities in the parameter ϵ_1 in the Panfilov model can result in the coexistence of different types of spatiotemporal behaviors in the same system. With $\epsilon_1^{out} = 0.01$ and $\epsilon_1^{in} = 0.02$ (see text) and a square inhomogeneity of side 40 mm, we obtain spatiotemporal chaos outside the inhomogeneity but quasiperiodic behavior inside it if it is placed at (70 mm, 80 mm). The pseudocolor plots in (A) and (B) are taken at $t = 2200$ ms and 3300 ms, respectively; the inter-beat interval (IBI), taken from a point inside the inhomogeneity (C) and from outside it (D), along with corresponding power spectra (E) and (F), obtained from time series of V from representative points. The discrete peaks in (E) can be indexed as $n_1\omega_1 + n_2\omega_2$ with the incommensurate frequencies $\omega_1 \simeq 11.23$ Hz and $\omega_2 \simeq 7.127$ Hz; (F) shows a broad-band background in the power spectrum.

channel conductance, to 0.00011, then to 0.00005, and finally to 0 (i.e., I_{CaL} channel block). Observe that, as G_{CaL} decreases, so does the APD; in addition, the shape of the AP also changes: the extent of the plateau region decreases and, eventually, the shallow minimum after the initial upstroke is lost. We now study the TNNP model in a square simulation domain of side 13.5 cm with the initial condition of Fig. 1 (A)(see main text). As we decrease G_{CaL} the spiral wave breaks up because the slope of the APD restitution curve steepens and eventually exceeds 1: This break up is shown in the pseudocolor plots of V, at $t = 3.2$ s, of Figs. 31 A, B, and C for $G_{CaL} = 0.000175$, 0.00011, and 0.0005, respectively. In Figs. 31 D, E, and F we show power spectra that have been obtained from time series of V recorded from the representative point (90 mm, 90 mm) during spiral-wave activity for $G_{CaL} = 0.000175$, 0.00011, and 0.00005, respectively. The discrete lines in the power spectrum of Figs. 31 D can be indexed by one fundamental frequency $\simeq 8.25$ Hz and integer multiples thereof; this is a signature of the periodic rotation of a single rotating spiral wave. The multiple strong peaks and the broad-band background in the power spectra of Figs. 31 E and F are indicative of quasiperiodic (with three fundamental frequencies $\omega_1 \simeq 8.25$ Hz, $\omega_2 \simeq 9$ Hz, and $\omega_3 \simeq 9.5$ Hz) and chaotic states, the latter associated with the break up of spiral waves. We get similar results increase the plateau Ca^{2+} conductance G_{pCa} instead of changing the L-type Ca^{2+} conductance.

In some cases we observe that the inhomogeneity does not have a significant qualitative effect on the dynamics of spiral waves; e.g., when the obstacle is at (45 mm, 45 mm), the pseudocolor plots of Fig. 32 show that the position of the spiral tip shifts towards bottom-left corner of the simulation domain but we still have a state with a single rotating spiral wave whose arms pass through the inhomogeneity. The periodic behaviors of time series V from a representative point in the simulation domain [Figs. 32 (E) and (H)] and the constant value of the associated IBI plots [Fig. 32(F) and (I)] indicates clearly the existence of a single rotating spiral wave in the medium; and the discrete lines in the power spectra [Figs. 32 (G) and (J)] give additional evidence for this. Like conduction inhomogeneities, ionic inhomogeneity can also remove spirals from the medium to leave the system in a quiescent state, e.g., when our G_{CaL} ionic inhomogeneity is at (45 mm, 22.5 mm) as shown in Fig. 33.

6 Elimination of spiral turbulence

In the main paper we have described the elimination of spiral-turbulence in the two-dimensional TNNP model and the three-dimensional Panfilov model. Here we give similar results for the two-dimensional Panfilov, LRI, and RPB models and additional results for the three-dimensional Panfilov model.

Figure 34 shows our results for the two-dimensional LRI model; here a control current of 20μA/cm^2, applied for 100 ms on a mesh that divides our square simulation domain of side 9 cm into 16 square cells of side 2.25 cm each, suffices to control spiral turbulence. The pseudocolor plots of V in Fig. 34 give (A) the initial spiral-turbulent state and its subsequent evolution after the initiation of the control pulse at (B) 100 ms, (C) 200 ms, and (D) 500 ms, after which all spiral turbulence disappears. Similar results from our simulations of the two-dimensional RPB model are given in Fig. 35; here a control current of 20μA/cm^2, applied for 100 ms on a mesh that divides our square simulation domain of side 11.52 cm into 25 square cells of side $\simeq 2.25$ cm each, suffices to control spiral turbulence. The pseudocolor plots of V in Fig. 35 give (A) the initial spiral-turbulent state and its subsequent evolution after the initiation of the control pulse at (B) 50 ms, (C) 200 ms, and (D) 400 ms, after which all spiral turbulence disappears.

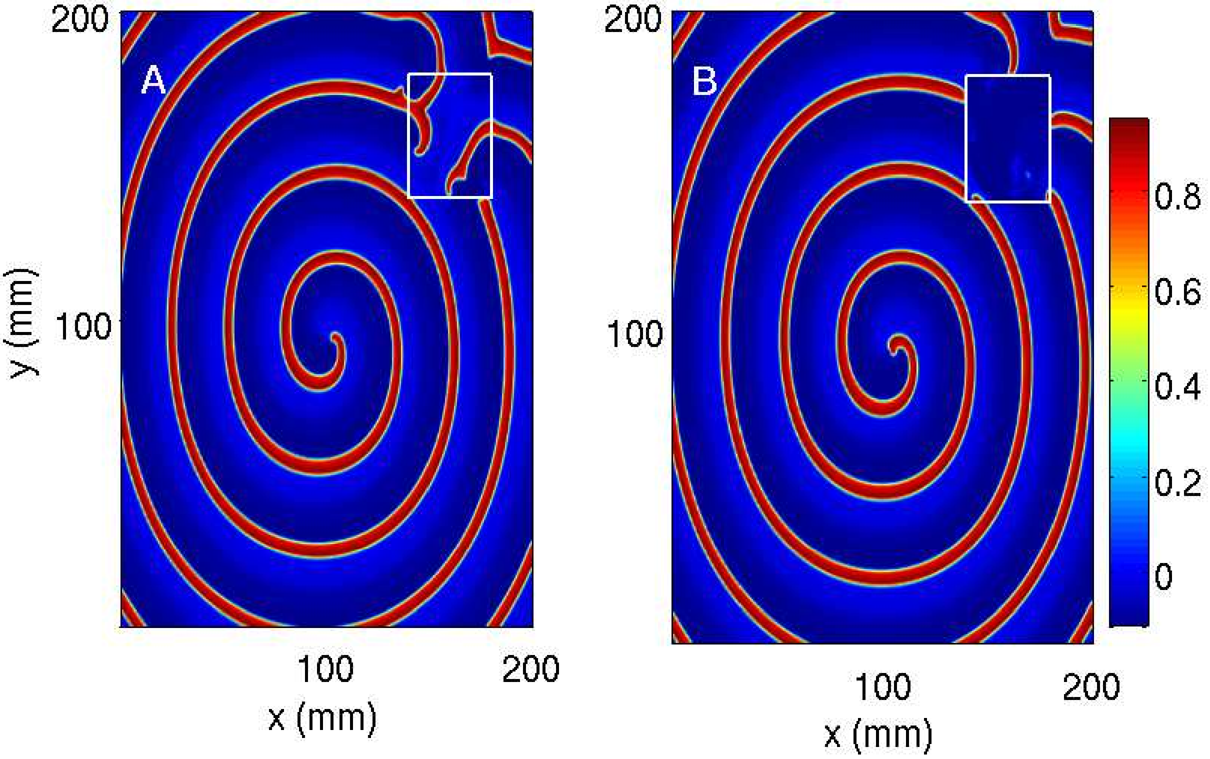

Figure 22: (Color online) Inhomogeneities in the parameter ϵ_1 in the Panfilov model can result in the coexistence of different types of spatiotemporal behaviors in the same system, as in Fig. 21. With $\epsilon_1^{out} = 0.03$ and $\epsilon_1^{in} = 0.01$ (see text) and a square inhomogeneity of side 40 mm, we obtain spatiotemporal chaos inside the inhomogeneity but periodic behavior inside it if it is placed at (140 mm, 140 mm). The pseudocolor plots in (A) and (B) are at $t = 2200$ ms and 3300 ms, respectively.

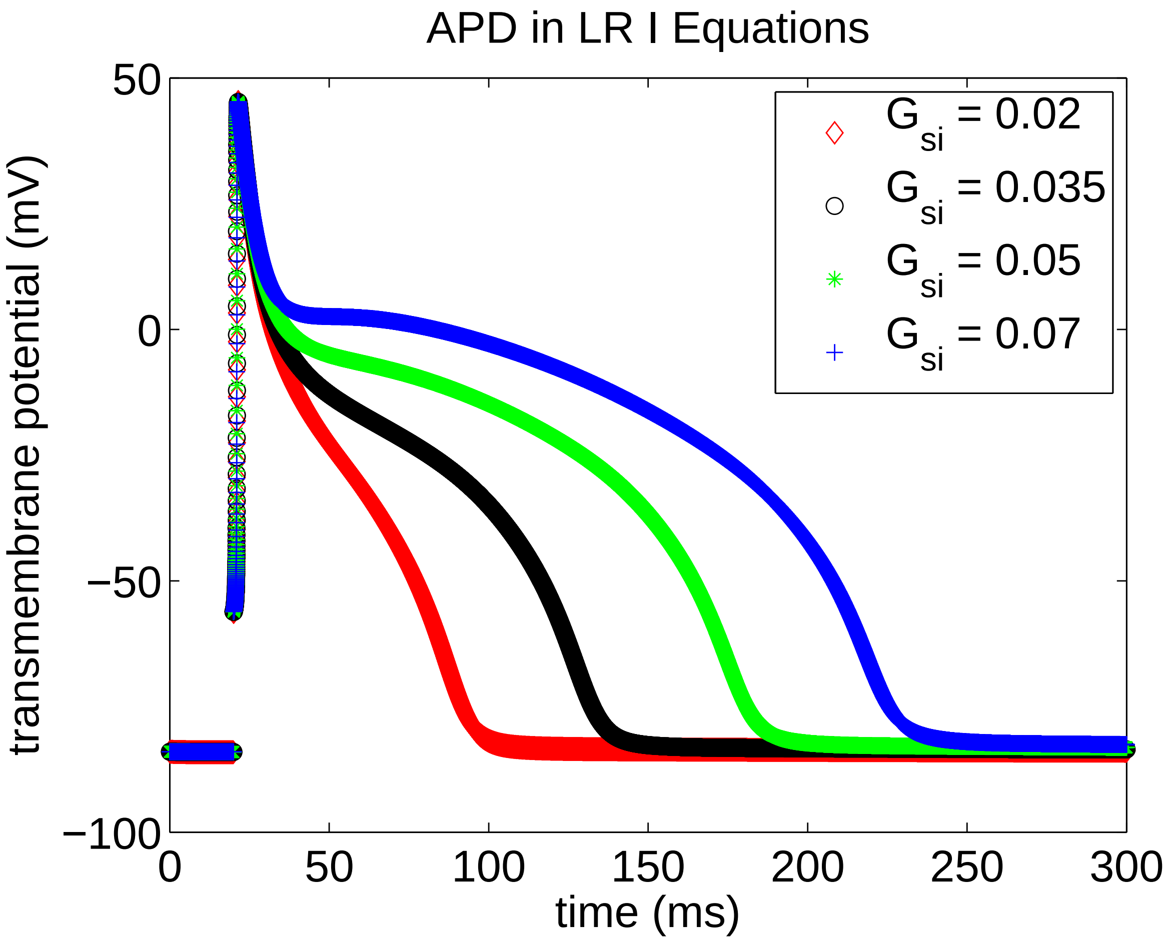

Figure 23: Superimposed plots of the action potentials in the LRI model illustrating their dependence on the conductance G_{si} for the slow inward calcium current. Four values of G_{si} are chosen to illustrate that the APD increases with G_{si}.

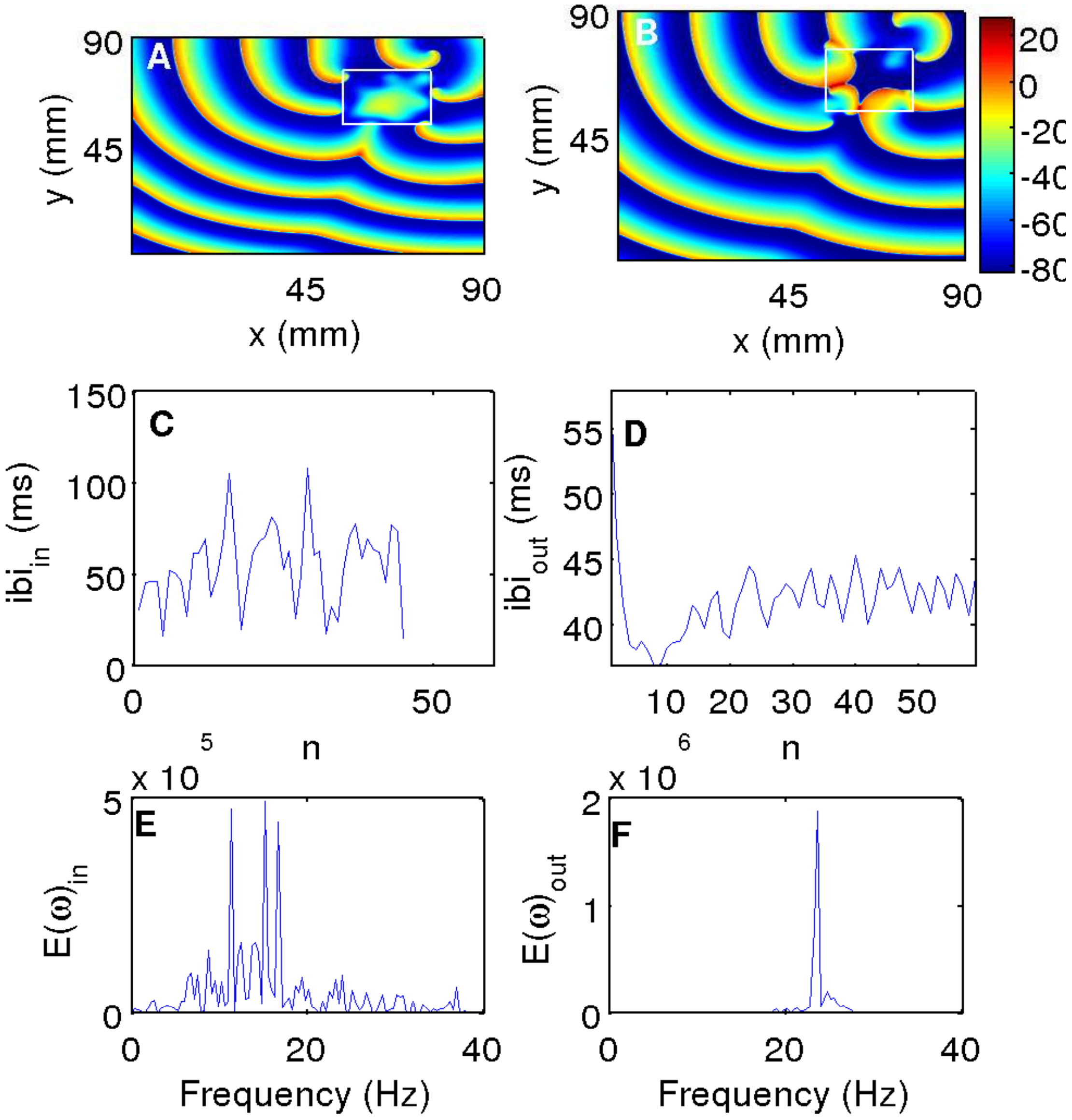

Figure 24: (Color online) Inhomogeneities in G_{si} in the LRI model can result in the coexistence of different types of spatiotemporal behaviors in the same system. With $G_{si}^{out} = 0.02$ and $G_{si}^{in} = 0.07$ in a square inhomogeneity of side 22.5 mm and a square simulation domain of side $L = 9$ cm, we obtain spatiotemporal chaos outside the inhomogeneity but periodic behavior inside it if it is placed at (63 mm, 63 mm). Pseudocolor plots of V are shown in (A) and (B); from representative points outside and inside the inhomogeneity we obtain (C) and (D), the plots of the IBI versus the beat number n, and the associated power spectra shown, respectively, in (E) and (F).

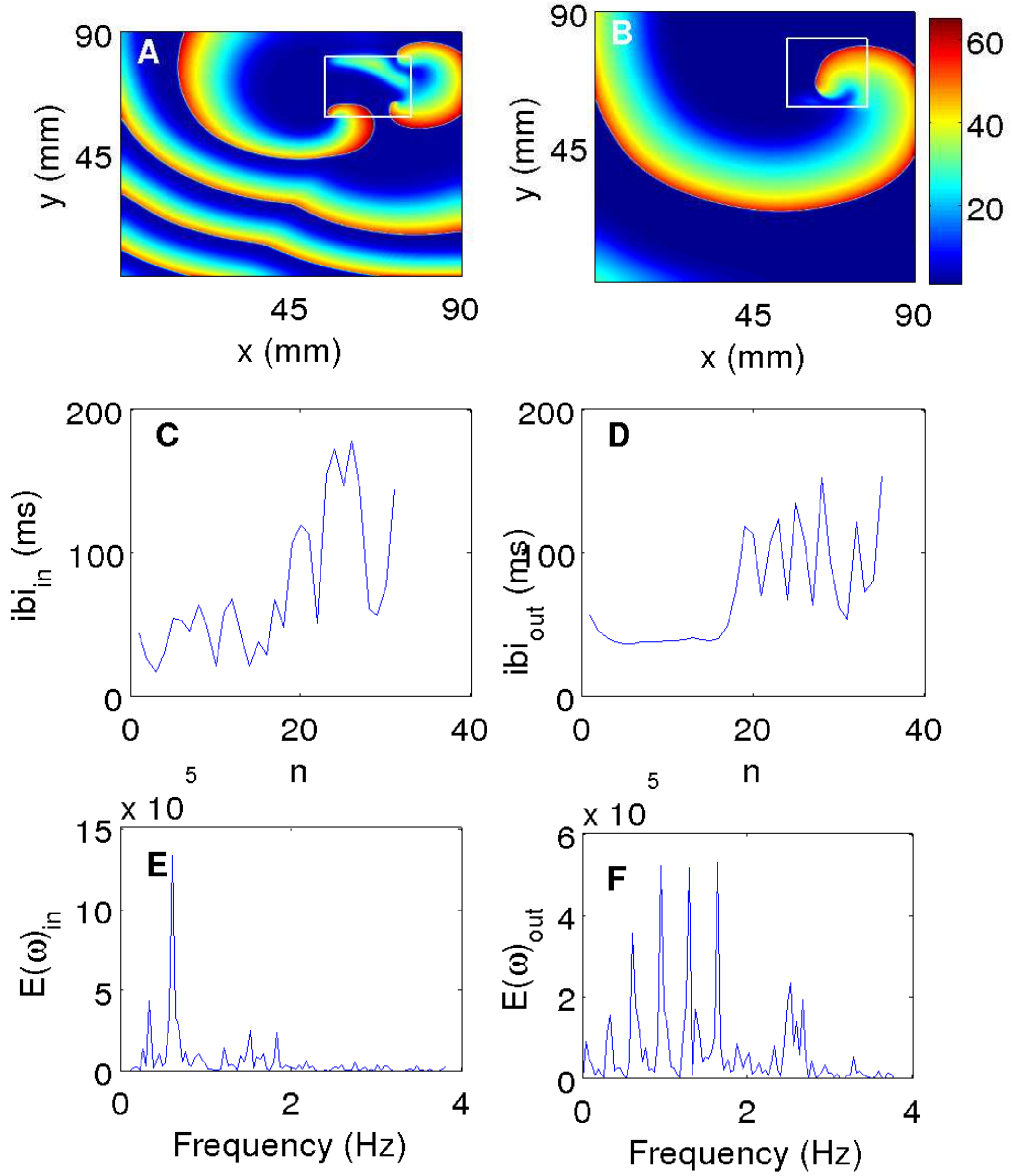

Figure 25: (Color online) A spiral wave anchoring to a G_{si} inhomogeneity in the LRI model: Parameters are the same as those in Fig. 24 but with G_{si}^{in}=0.07 and G_{si}^{out} = 0.02 and the ionic inhomogeneity at (49.5 mm, 58.5 mm). The pseudocolor plots of V shown in (A) and (B) are at $t = 0.8$s and 1.2 s, respectively. (C) and (E) show the plot of the IBI versus the beat number n and the associated power spectrum calculated from the time series of V from a representative point inside the inhomogeneity; and (D) and (F) are analogous plots for a representative point outside the inhomogeneity.

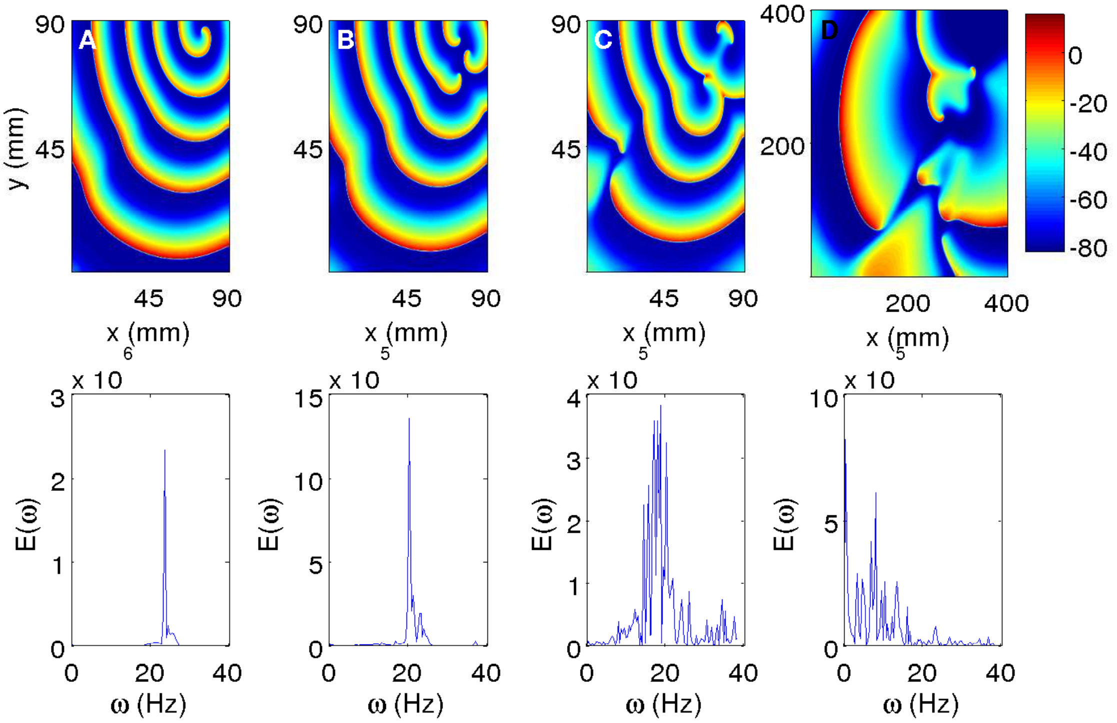

Figure 26: (Color online) The effects of changing G_{si} in the LRI model: Pseudocolor plots of V (top panel), which are obtained after $t = 100$ ms, and the associated power spectra (bottom panel) that we calculate from a time series of V from a representative point; we show plots for $G_{si} = 0.02$, 0.035, 0.05, and 0.07 (units as in Sec. 2 A) [2]. Data for the time series in Fig. 26 are collected for 262144 iterations, i.e., 2621.44 ms. As G_{si} increases, a simple rotating spiral evolves to a state with broken spiral waves and spatiotemporal chaos; the power spectra obtained from the time series of V, taken from a representative point in the simulation domain, also mirror this evolution:

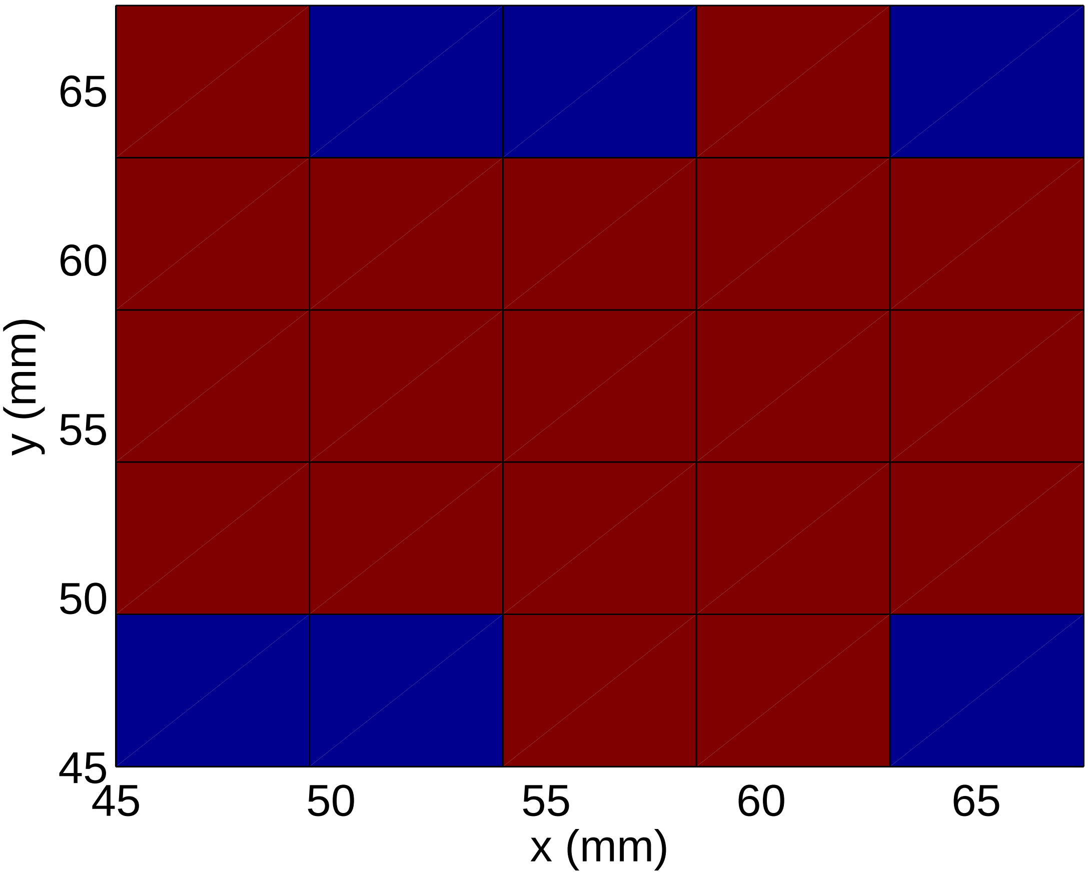

Figure 27: (Color online) Detail of the stability diagram for the LRI model with the initial condition described in the text: A square G_{si} inhomogeneity of side 22.5 mm is placed at different positions in a square simulation domain with side $L = 90$ mm. We have G_{si}^{in}=0.02 inside the inhomogeneity in a simulation domain where G_{si}^{out}=0.07. For each one of these positions of the obstacle we determine the final state of the system; the colors of the small squares, of side $l_p = 4.5$ mm, indicate the final state of the system when the position of the bottom-left corner of the obstacle coincides with that of the small square (red and blue denote ST and Q, respectively).

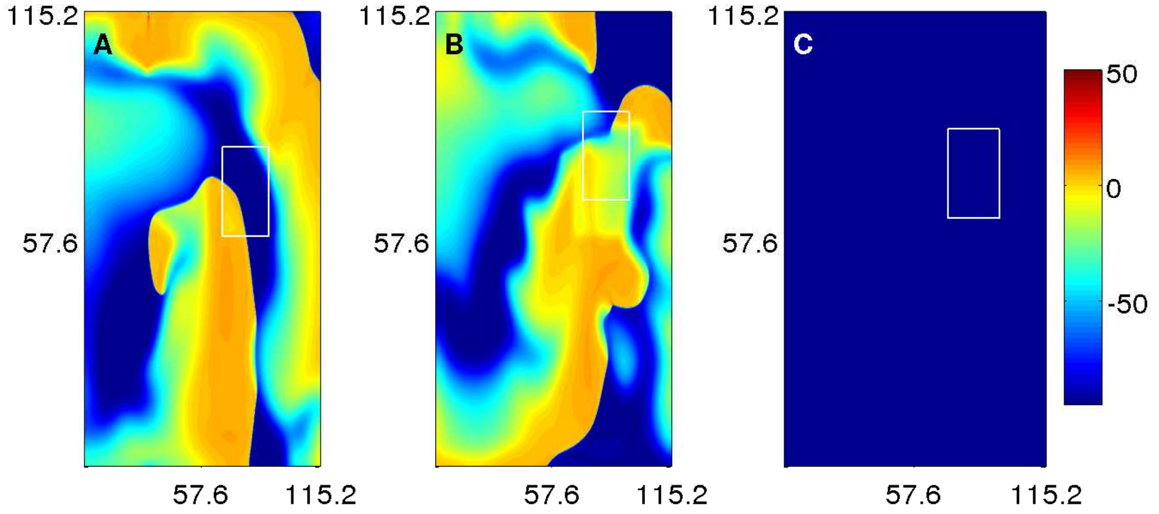

Figure 28: (Color online) Pseudocolor plots of V from our simulation of the RPB model in a square simulation domain with side $L = 11.52$ cm and a square G_{si} inhomogeneity of side 22.5 mm with $G_{si}^{out} = 0.064$ and $G_{si}^{in} = 0.02$ placed at (58.5 mm, 67.5 mm). The plots in A, B, and C are at $t = 0.4, 1.2, 2$ s, respectively; the spiral wave has been absorbed by the boundaries by $t = 2$ s (C).

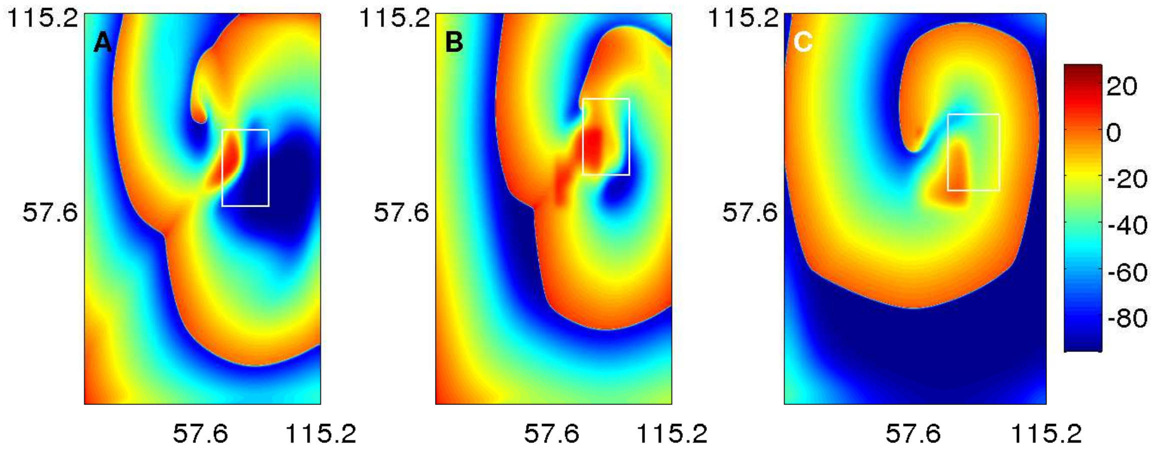

Figure 29: (Color online) Pseudocolor plots of V in the RPB model with the parameters of Fig. 28 but with $G_{si}^{in} = 0.064$ and $G_{si}^{out} = 0.02$. This results in a single rotating spiral anchored at the inhomogeneity as in the plots of A, B, and C at times $t = 0.2, 0.6, 1.0$s, respectively.

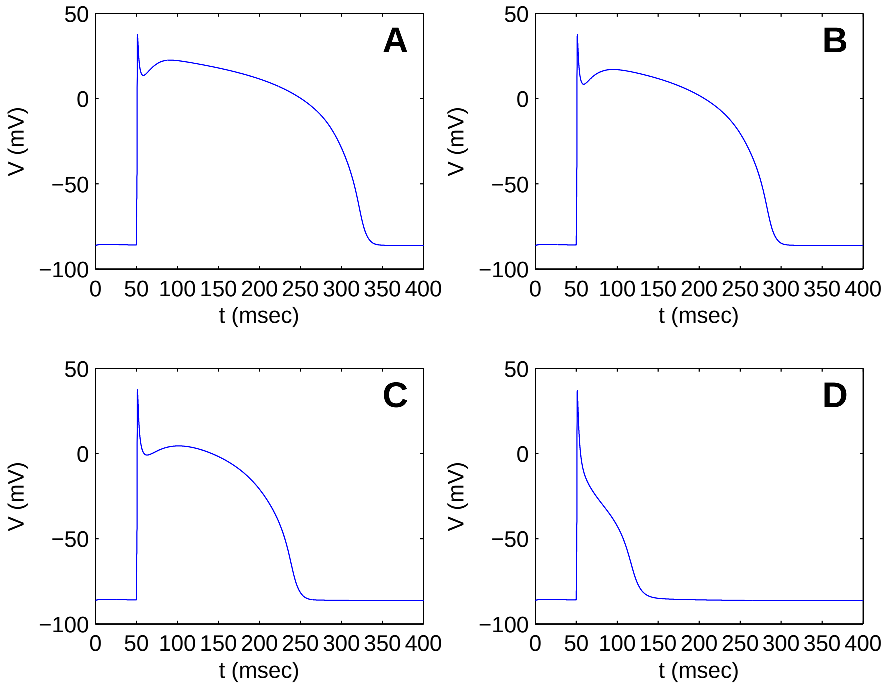

Figure 30: Effect of the G_{CaL} conductance on the action potential in the TNNP model: (A) G_{CaL}=0.000175 (maximal channel conductance); (B) G_{CaL}=0.00011; (C) G_{CaL}=0.00005; and (D) G_{CaL}=0 (I_{CaL} channel block).

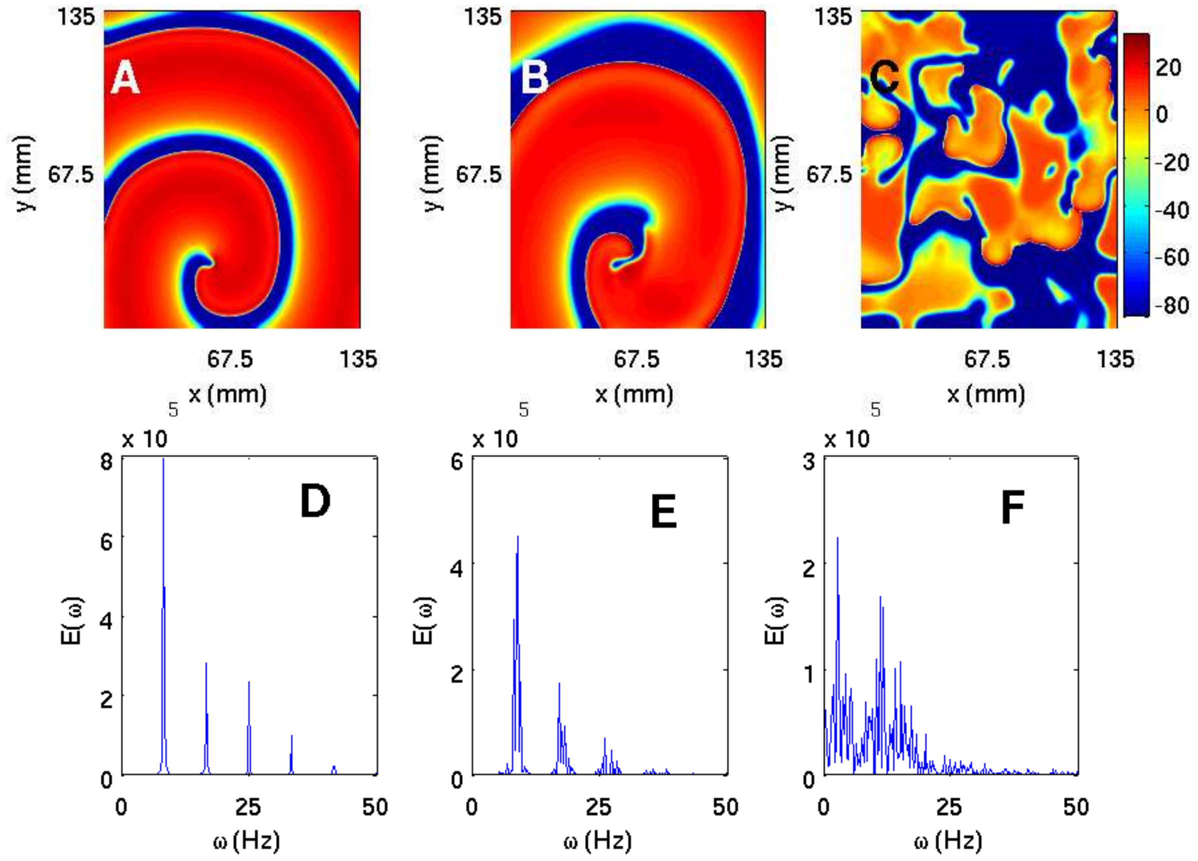

Figure 31: (Color online) The effect of G_{CaL} on spiral waves in the TNNP model shown via pseudocolor plots of V at time $t = 3.2$ s and (A) $G_{CaL} = 0.000175$, (B) $G_{CaL} = 0.00011$, and (C) $G_{CaL} = 0.00005$. Panels (D), (E), and (F) show the corresponding power spectra of V (from a time series of 200000 iterations after the removal of the initial 80000 iterations) from the representative point (90 mm, 90 mm) in the simulation domain. These plots indicate that as G_{CaL} decreases the system goes from a state with a single rotating spiral wave to the spiral-turbulence state.

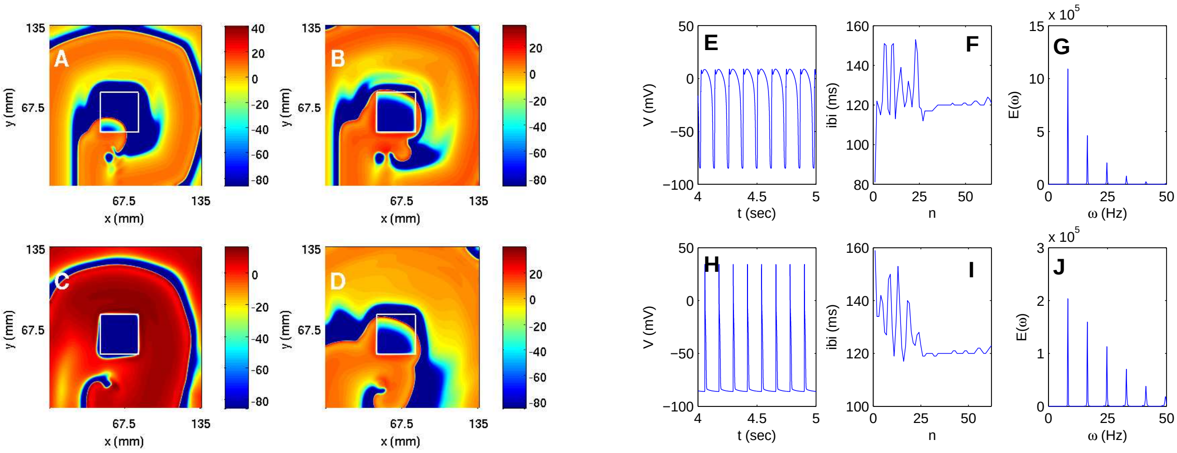

Figure 32: (Color online) The effect of a square G_{CaL} inhomogeneity, of side 33.75 mm in a square simulation domain of side 135 mm, with $G_{CaL}^{out} = 0.000175$ (maximal value) and $G_{CaL}^{in} = 0.00003$, and placed at $(45\ mm, 45\ mm)$, on spiral-wave dynamics in the TNNP model: pseudocolor plots of V are shown at (A) $t = 0.16$ s, (B) $t = 0.32$ s, (C) $t = 1.2$ s, and (D) 2 s. (E) the time series of V (from a sample of 50000 iterations after the removal of the initial 200000 iterations) taken from the point (11.25 mm, 11.25 mm) that lies outside the inhomogeneity. Associated plots of (F) the IBI versus the beat number n (a sample of 400000 iterations) and (G) the power spectrum of V (from a sample of 200000 iterations after the removal of the initial 200000 iterations) indicating periodic temporal evolution [the peaks in the power spectrum can be indexed (see text) in terms of one frequency (8.25 Hz) and its harmonics]. Figures (H), (I), and (J) are the analogs of (E), (F), and (I), respectively, when data for V are recorded from the point (56.25 mm, 56.25 mm) that lies inside the inhomogeneity; here too we have periodic temporal evolution with one underlying fundamental frequency (8.25 Hz). In both (F) and (I) the IBI approaches a constant value ($\simeq 118$ ms).

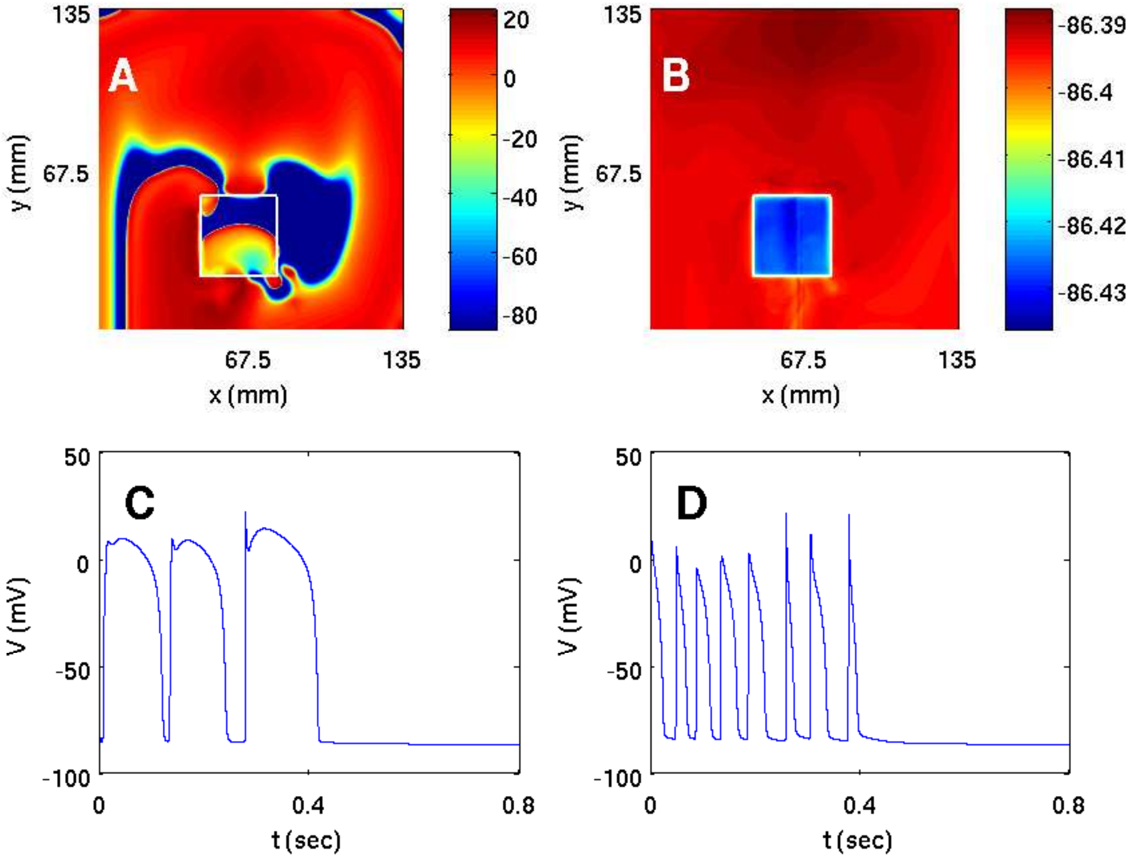

Figure 33: (Color online)The analog of Fig. 32 but with the G_{CaL} inhomogeneity at (45 mm, 22.5 mm). The pseudocolor plots of V at (A) 0.32 s and (B) 1.2 s and the representative time series of V (from a sample of 40000 iterations) for (C) a point outside (11.25 mm, 11.25 mm) and (D) a point inside (56.25 mm, 33.75 mm) the inhomogeneity show clearly that the spiral wave moves away from the simulation domain in this case.

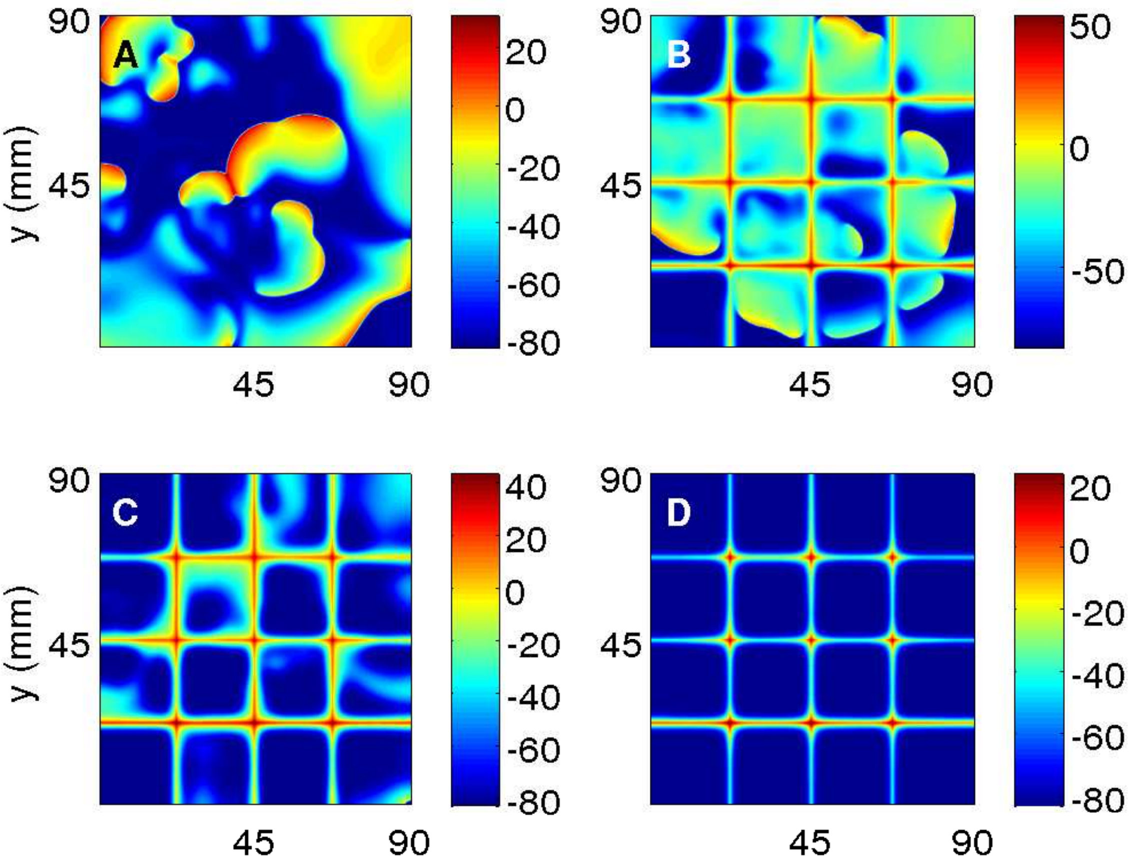

Figure 34: (Color online) Suppressing spiral turbulence in the LRI model: here a control current of 20 $\mu A/cm^2$, applied for 100 ms on a mesh that divides our square simulation domain of side 9 cm into 16 square cells of side 2.25 cm each, suffices to control spiral turbulence. Pseudocolor plots of V: (A) the initial spiral-turbulent state and its subsequent evolution after the initiation of the control pulse at (B) 100 ms, (C) 200 ms, and (D) 500 ms, after which all spiral turbulence disappears.

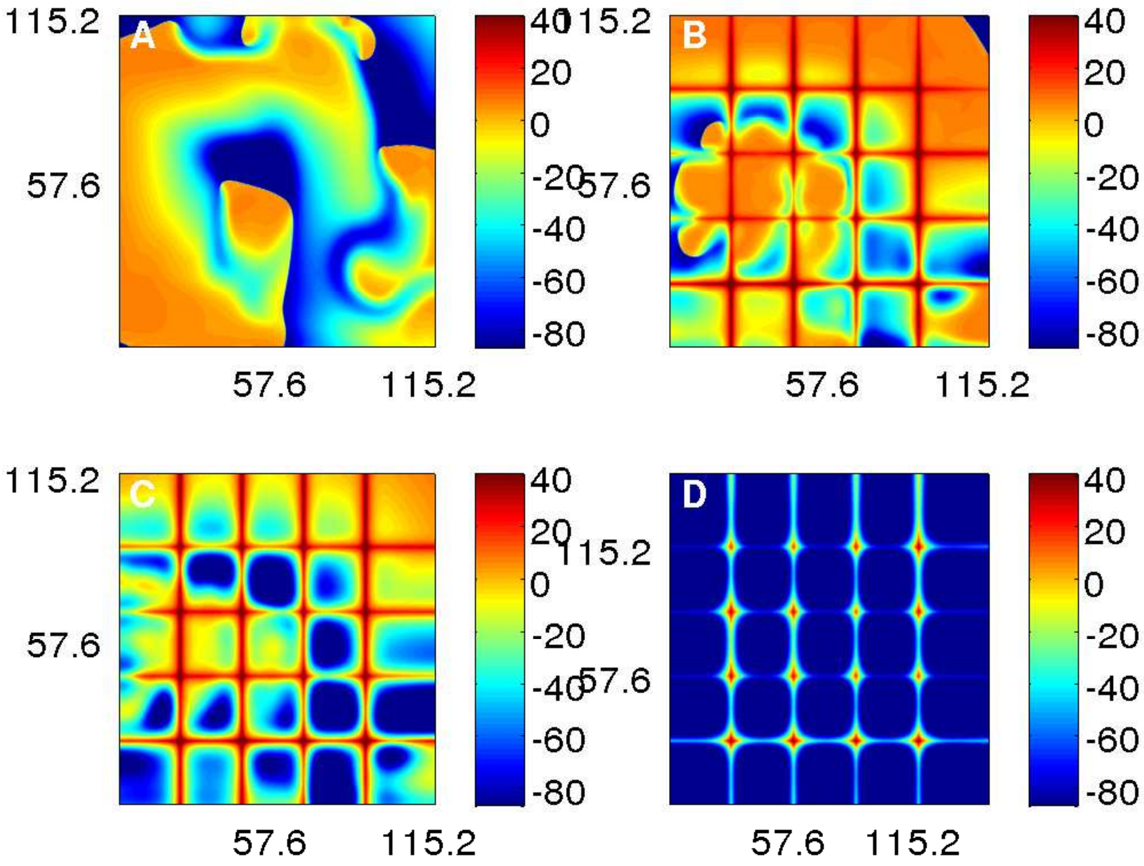

Figure 35: (Color online) Suppressing spiral turbulence in the RPB model: here a control current of 20 μA/cm^2, applied for 100 ms on a mesh that divides our square simulation domain of side 11.52 cm into 25 square cells of side $\simeq$ 2.25 cm each, suffices to control spiral turbulence. The pseudocolor plots of V give (A) the initial spiral-turbulent state and its subsequent evolution after the initiation of the control pulse at (B) 50 ms (C) 200 ms and (D) 400 ms, after which all spiral turbulence disappears.

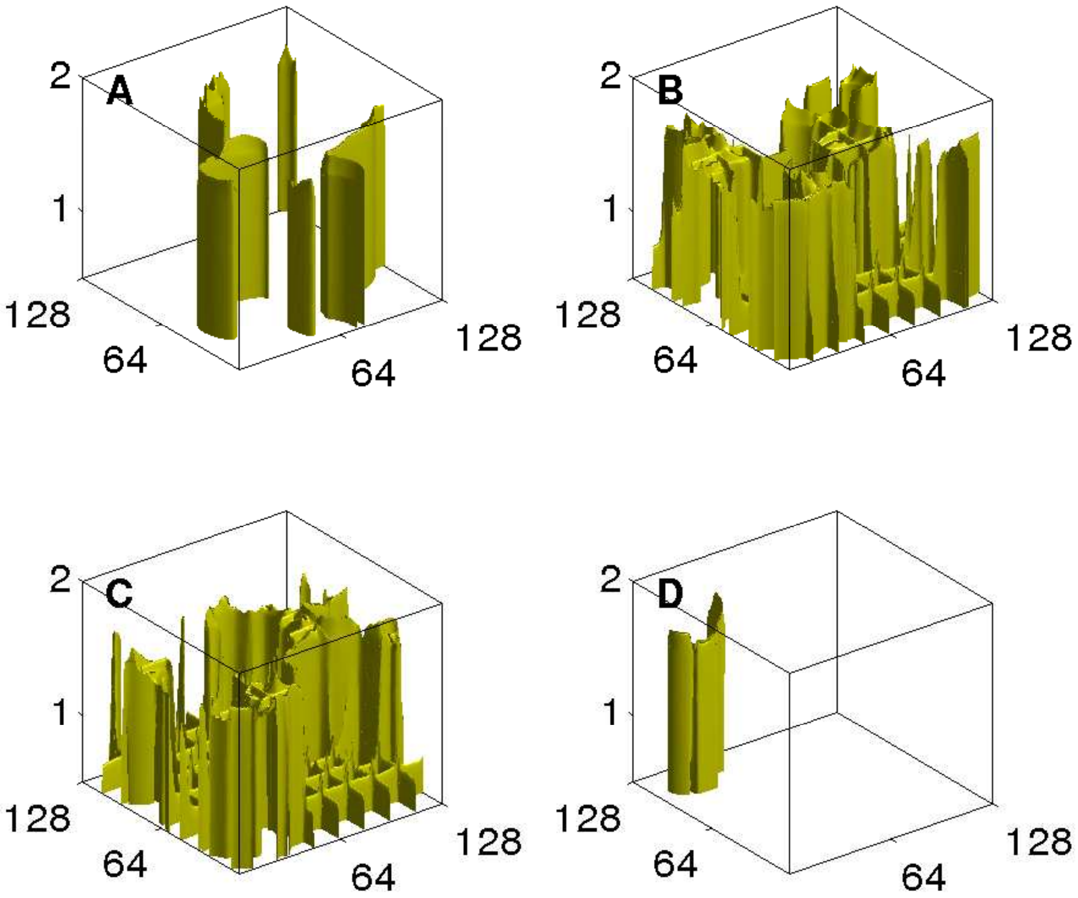

Figure 36: (Color online) The control of scroll-wave turbulence in our simulation of the 3D Panfilov model in a domain of size $128 \times 128 \times 2$ mm^3: The initial state with scroll-wave turbulence is shown via an iso-surface plot of V in (A); the control pulse of strength 0.48 is applied on a mesh with square cells, each of side 16 mm, on the bottom face of the simulation domain for 748 ms; the evolution of this state after the initiation of the control is depicted at (B) 440 ms, (C) 660 ms, and (D) 880 ms. By 880 ms the last part of the scroll wave is moving out of the simulation domain and by 1100 ms the system is completely quiescent (not shown here).

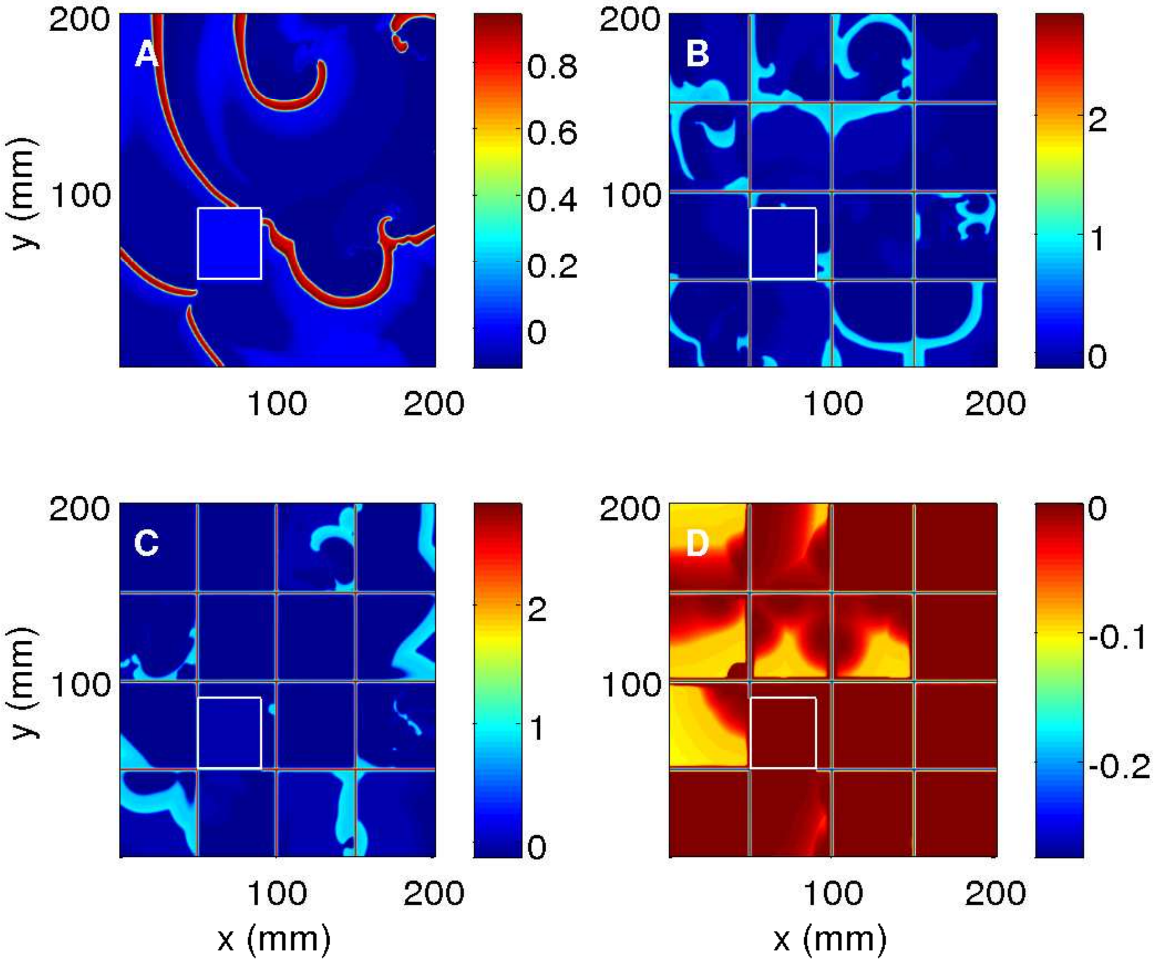

Figure 37: (Color online) Suppressing spiral turbulence in the 2D Panfilov model in the presence of an inhomogeneity: the square simulation domain has side $L = 20$ cm, the obstacle is a square with side $l = 4$ cm and is placed at (50 mm, 50 mm), and the simulation domain is covered by a mesh with $K = 4$, i.e., the square unit cell of this mesh has a lattice constant $\ell = L/K = 5$ cm. Had we not provided a control stimulus on the mesh, the position of the inhomogeneity here is such that spiral turbulence would have been obtained as in (A). We now apply the control pulse, of amplitude 0.8, for 770 ms. The pseudocolor plots of V at 220 ms (B), 770 ms (C), and 1500 ms (D), after the initiation of the control pulse, show that all spiral waves are removed from the system by this control scheme even though an inhomogeneity is present.

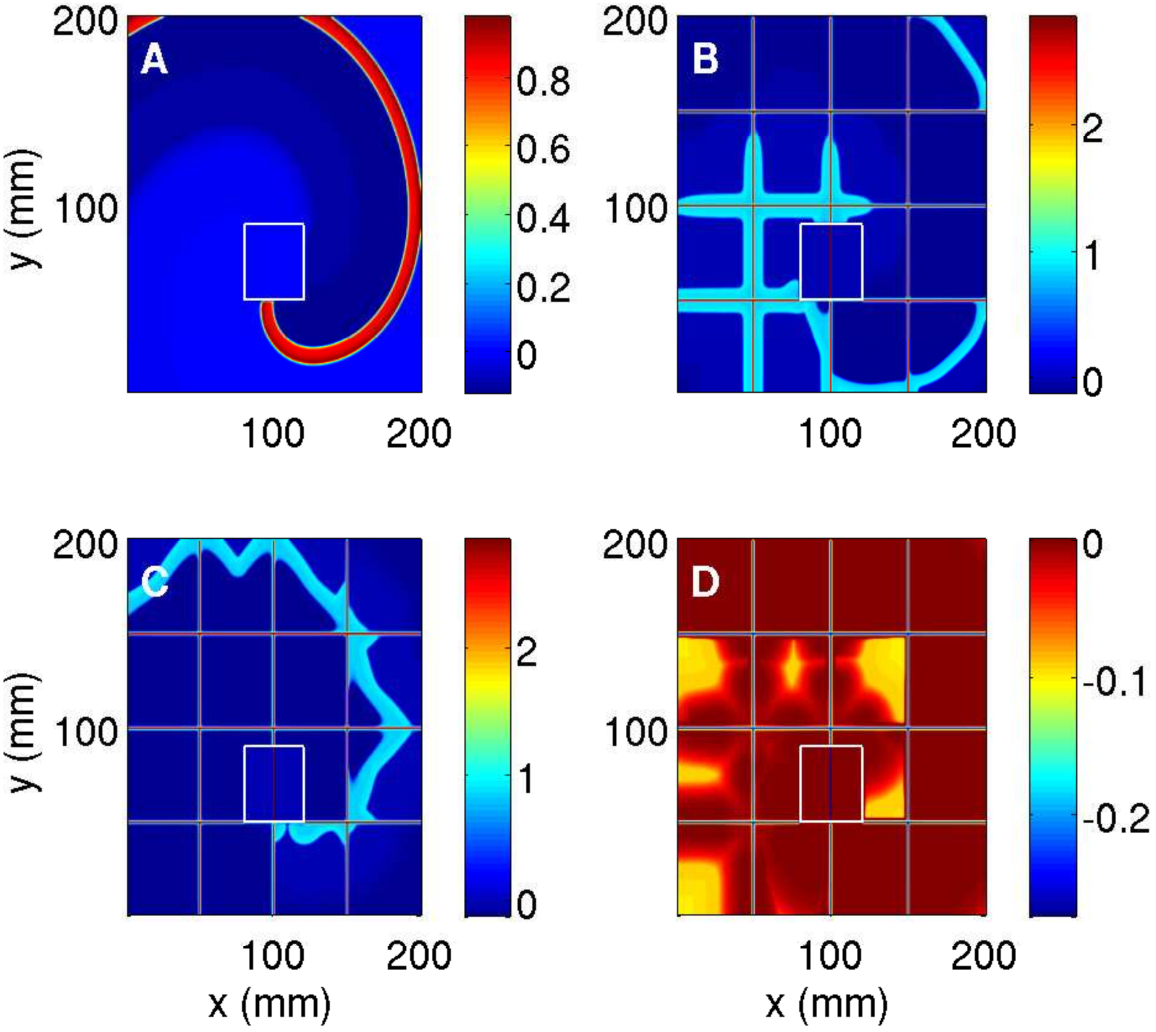

Figure 38: (Color online) Suppressing an anchored spiral in the 2D Panfilov model in the presence of an inhomogeneity: This is as in Fig. 37, but now the obstacle is placed at (80 mm, 50 mm). (A) Pseudocolor plot of V showing that initial spiral wave anchored to the obstacle. We now apply the control pulse, of amplitude 0.8, for 770 ms. The pseudocolor plots of V at 220 ms (B), 770 ms (C), and 1500 ms (D), after the initiation of the control pulse, show that all spiral waves are removed from the system by this control scheme even though an inhomogeneity is present.

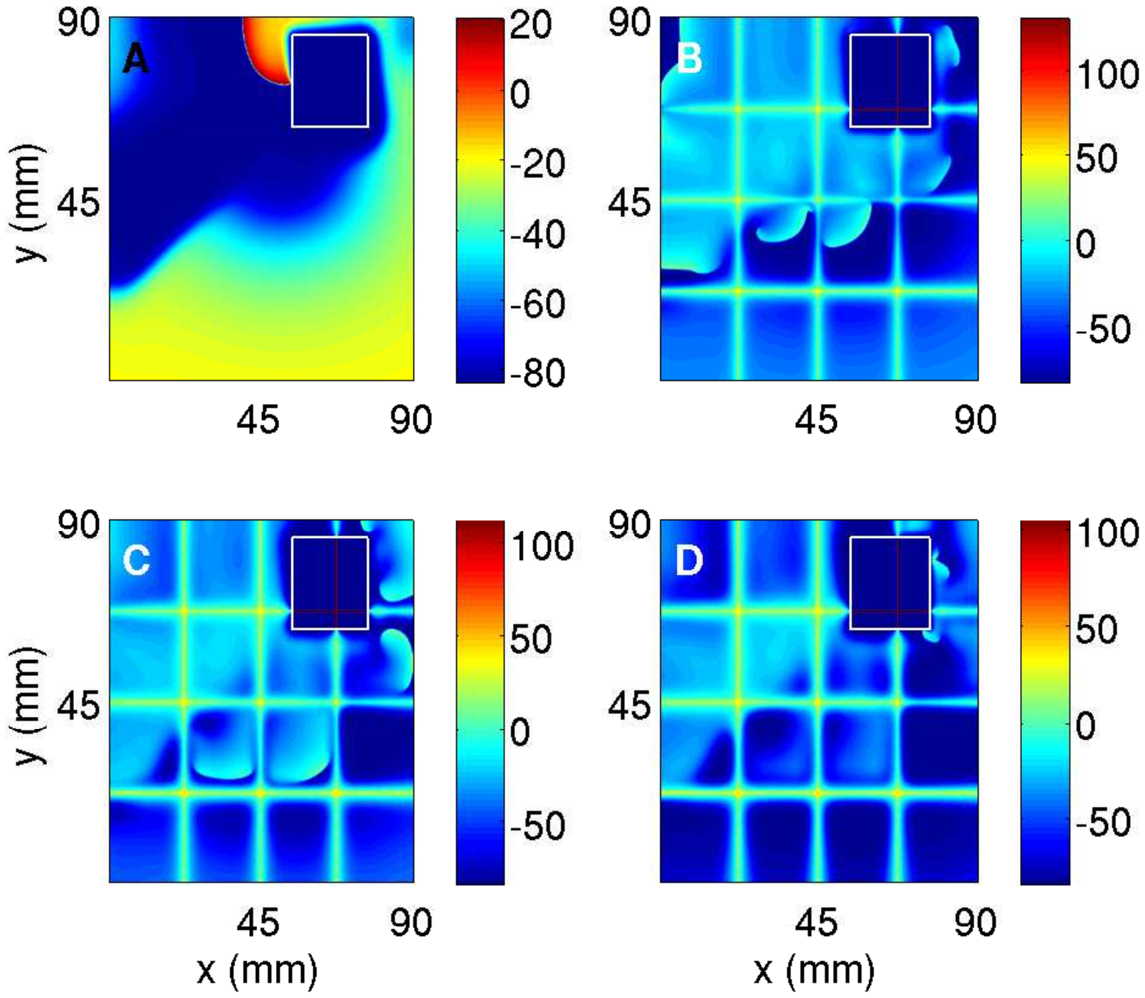

Figure 39: (Color online) Suppressing spiral turbulence in the 2D LRI model in the presence of an inhomogeneity: here a control current of 20 μA/cm^2, applied for 100 ms on a mesh that divides our square simulation domain of side 9 cm into 16 square cells of side 2.25 cm each, suffices to control spiral turbulence, even though there is a square obstacle of side 2.25 cm placed at (63 mm, 54 mm). The pseudocolor plots of V give (A) the initial state with an anchored spiral and its subsequent evolution after the initiation of the control pulse at (B) 100 ms (C) 200 ms and (D) 500 ms, after which all spiral turbulence disappears.

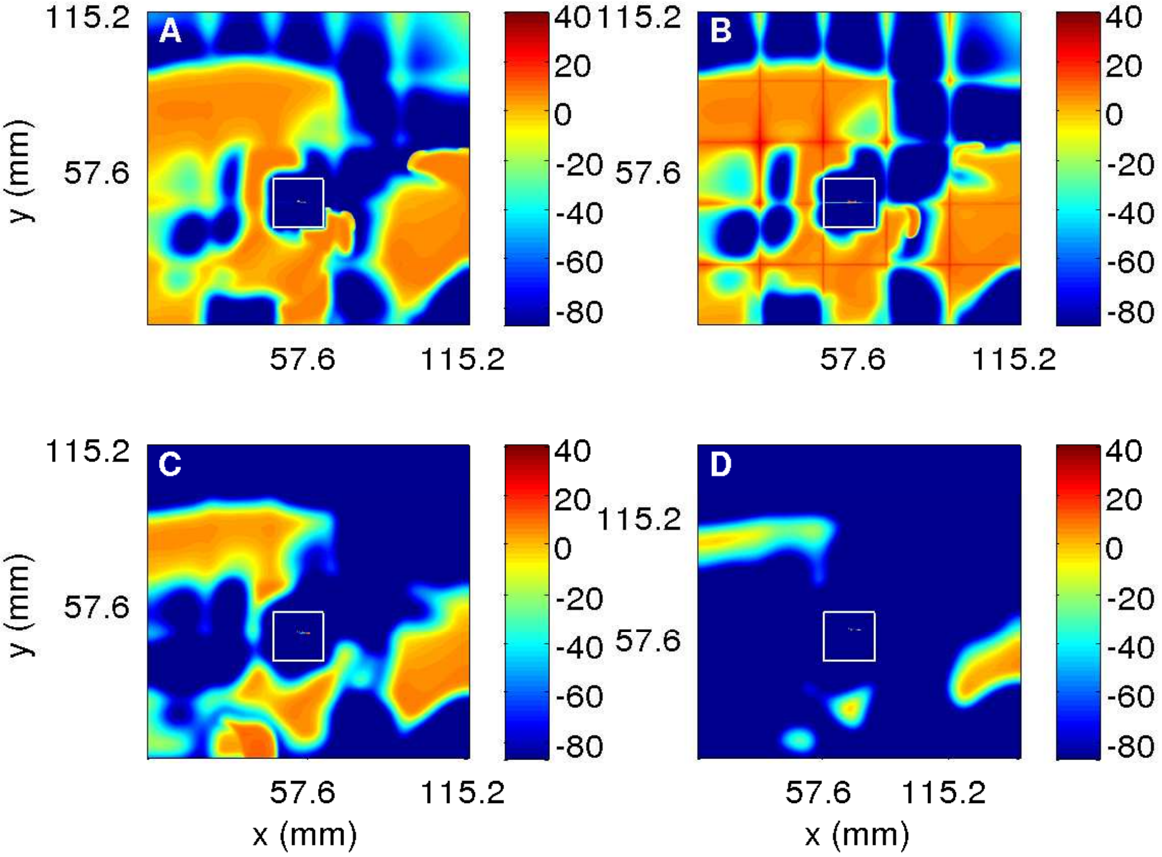

Figure 40: (Color online) Suppressing spiral turbulence in the 2D RPB model in the presence of an inhomogeneity: here a control current of 20 μA/cm^2, applied for 100 ms on a mesh that divides our square simulation domain of side 11.52 cm into 25 square cells of side $\simeq$ 2.25 cm each, suffices to control spiral turbulence, even though there is a square obstacle of side 2.25 cm placed at (45 mm, 36 mm). The pseudocolor plots of V give (A) the initial state with spiral turbulence and its subsequent evolution after the initiation of the control pulse at (B) 50 ms, (C) 200 ms, and (D) 400 ms, after which all spiral turbulence disappears.

In the three-dimensional Panfilov model, even if we apply a control pulse on a mesh on one of the $L \times L$ faces of the $L \times L \times L_z$ simulation domain, we can suppress broken scroll waves (the three-dimensional analogs of broken spiral waves) in the entire medium, provided that $L_z < 2$ mm. We show in Fig. 36 the successful application of such a control scheme in a $128 \times 128 \times 2$ simulation domain via isosurfaces of the transmembrane potential V. The initial state with spiral turbulence is shown in Fig. 36 A; the control pulse of strength 0.48 is applied on a mesh with square cells, each of side 16 mm, on the bottom face of the simulation domain for 748 ms; the evolution of this state after the initiation of the control is depicted at (B) 440 ms, (C) 660 ms, and (D) 880 ms. By 880 ms the last part of the scroll wave is moving out of the simulation domain and by 1100 ms the system is completely quiescent.

The control scheme proposed in Ref. [9] works even in the presence of an inhomogeneity. We first illustrate this for the two-dimensional Panfilov model via the pseudocolor plots of V in Figs. 37 and 38; the square simulation domain has side $L = 20$ cm, the obstacle is a square with side $l = 4$ cm, and the simulation domain is covered by a mesh with $K = 4$, i.e., the square unit cell of this mesh has a lattice constant $\ell = L/K = 5$ cm. The control voltage that we apply on the mesh does does not create any target waves

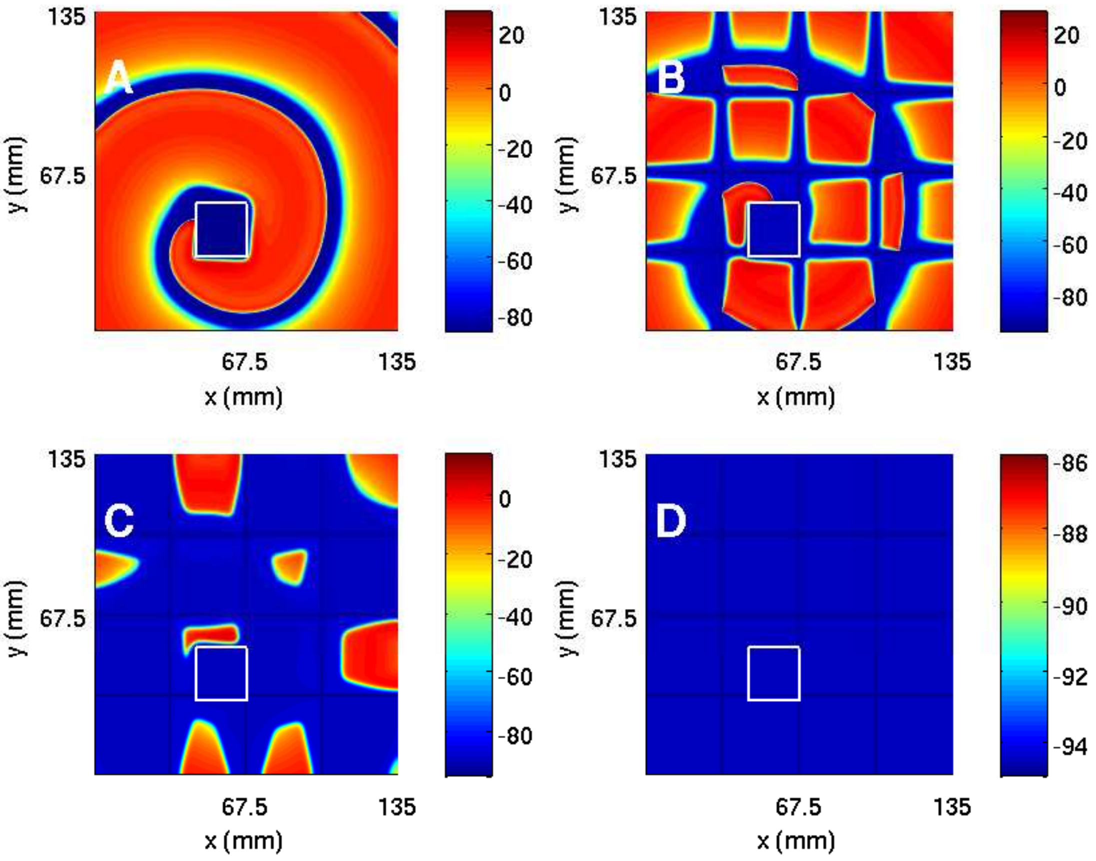

Figure 41: (Color online) Suppressing an anchored spiral in the 2D TNNP model in the presence of an inhomogeneity: A control pulse of amplitude 27.75 μA/cm^2 is applied for $t = 20$ ms on a mesh as described in the paper. The square obstacle is at (45 mm, 31.5 mm) and has side $l = 22.5$ mm. Without control the spiral rotates around the obstacle as shown in Fig. 7 in the main paper. The pseudocolor plots of V in (A) 0 ms, (B) 24 ms, (C) 80 ms, and (D) 280 ms, after the intiation of the control, show the suppression of the spiral wave.

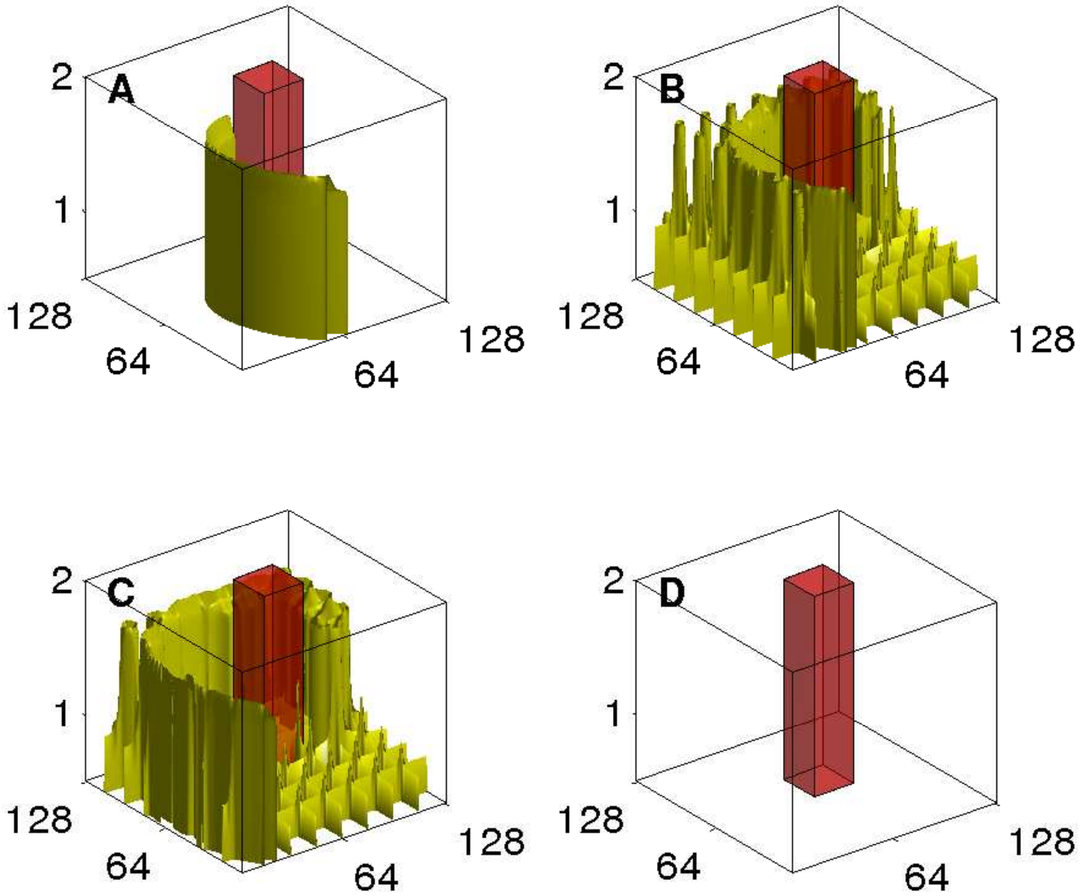

Figure 42: (Color online) Elimination of an anchored scroll wave in our 3D Panfilov-model simulation in a $128 \times 128 \times 2$ mm^3 simulation domain in the presence of an obstacle of size $25 \times 25 \times 2$ mm^3 placed at (60 mm, 60 mm): The scroll wave is anchored to the obstacle in this case as shown by the V iso-surface in (A). We now apply a control pulse of strength 0.48 on a mesh with square cells, each of side 16 mm, on the bottom face of the simulation domain for 748 ms; the evolution of the V iso-surfaces, after the initiation of the control, is depicted at (B) 220 ms, (C) 440 ms, and (D) 880 ms, by which time scroll waves have left the simulation domain and it is completely quiescent.

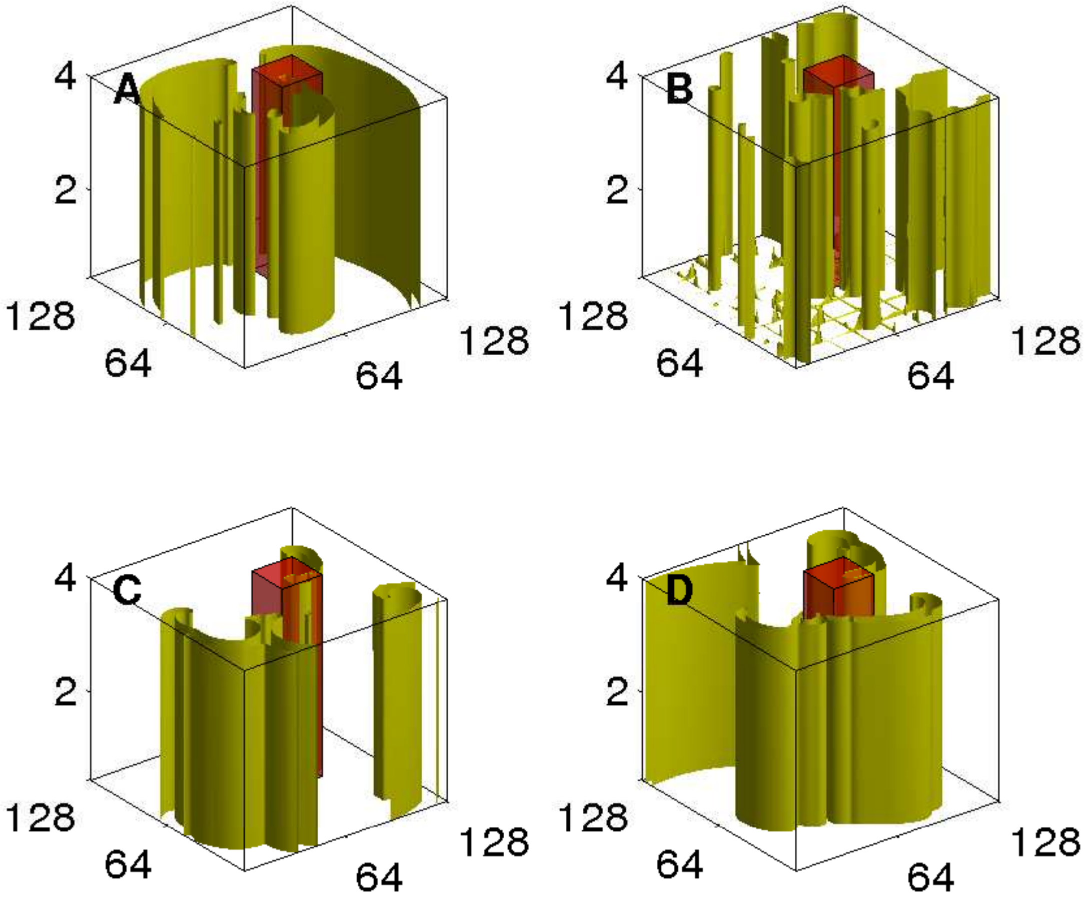

Figure 43: (Color online) Unsuccessful attempt to control scroll-wave turbulence in our 3D Panfilov-model simulation in a $128 \times 128 \times 4$ mm^3 simulation domain in the presence of an obstacle of size $25 \times 25 \times 4$ mm^3 placed at (70 mm, 60 mm): The scroll wave breaks up in this case as shown by the V iso-surface in (A). We now apply control pulses of strength 0.48 on a mesh with square cells, each of side 16 mm, on the bottom face of the simulation domain: instead of using a single long pulse, we use a series of 32 short pulses, each of duration $\tau_w = 2$ iterations (i.e., 0.22 ms), and separated by an interval of $\tau_{ip} = 200$ iterations. The evolution of the V iso-surfaces, after the initiation of the control, is depicted at (B) 220 ms, (C) 440 ms, and (D) 880 ms; scroll waves turbulence persists even after 880 ms.

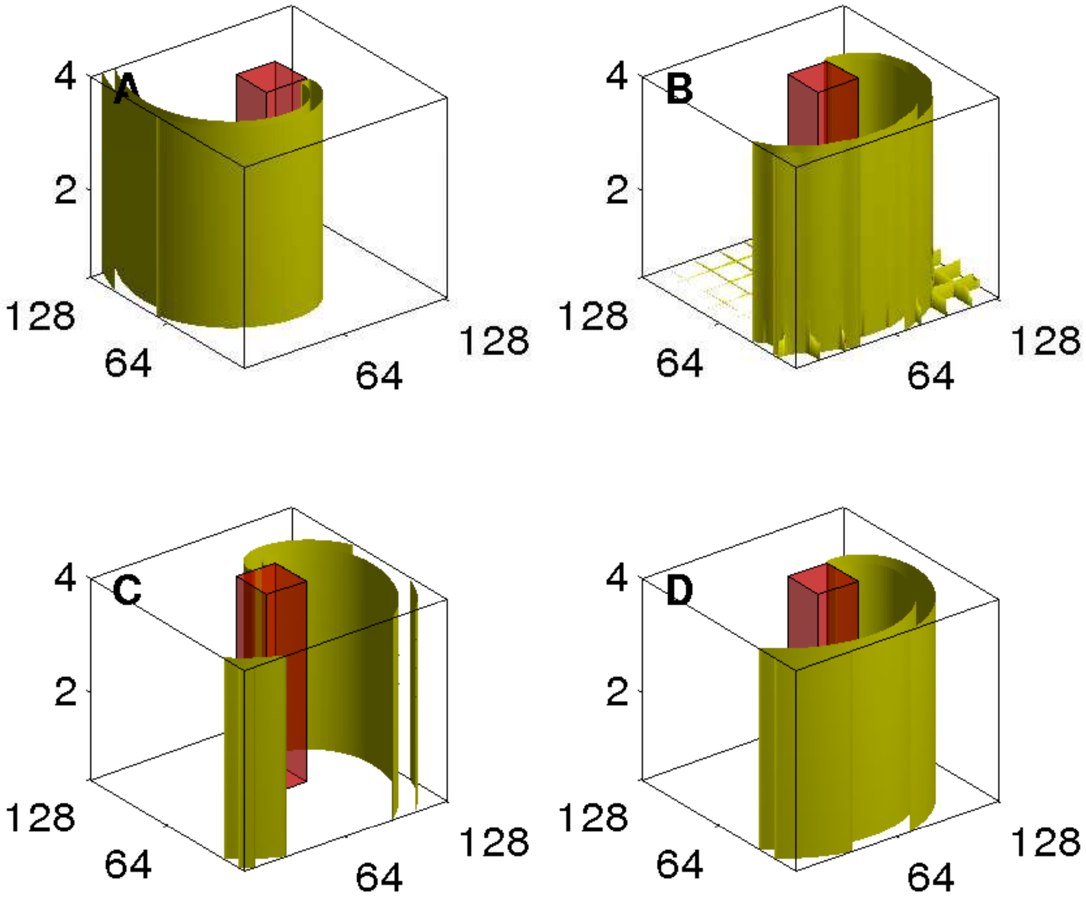

Figure 44: (Color online) Unsuccessful attempt to control an anchored scroll wave in our 3D Panfilov-model simulation in a $128 \times 128 \times 4$ mm^3 simulation domain in the presence of an obstacle of size $25 \times 25 \times 4$ mm^3 placed at (60 mm, 60 mm): The anchored scroll wave is shown by the V iso-surface in (A). We now apply control pulses of strength 0.48 on a mesh with square cells, each of side 16 mm, on the bottom face of the simulation domain: instead of using a single long pulse, we use a series of 32 short pulses, each of duration $\tau_w = 2$ iterations (i.e., 0.22 ms), and separated by an interval of $\tau_{ip} = 200$ iterations. The evolution of the V iso-surfaces, after the initiation of the control, is depicted at (B) 220 ms, (C) 440 ms, and (D) 880 ms; the anchored scroll wave persists even after 880 ms.

that can interact with the obstacle to produce secondary spiral waves that can later break up and sustain spiral turbulence; the small waves created by the control stimulus collapse inside a mesh unit cell soon after they are born. Had we not provided a control stimulus on the mesh, the positions of the inhomogeneities in Figs. 37 and 38 are such that spiral turbulence would have been obtained in the former (Fig. 37 A) and a single rotating spiral wave, anchored at the obstacle (Figs. 38 A), in the latter. We now apply the control pulse, of amplitude 0.8, for 770 ms. The pseudocolor plots of V, displayed in Figs. 37 and 38 at 220 ms (B), 770 ms (C), and 1500 ms (D) after the initiation of the control pulse, show that all spiral waves are removed from the system by this control scheme even though an inhomogeneity is present.

From Ref. [9], the dimensioned value of the control pulse is 122.22 μA/ms^2 times the dimensionless value. So a control pulse of 0.8 would correspond to 97.78 μA/ms^2.

The control scheme of Ref. [9] is also successful in eliminating spiral turbulence in the realistic LRI, RPB, and TNNP models even in the presence of conduction inhomogeneities. We show this by illustrative simulations below: Figure 39 shows our results for the two-dimensional LRI model; here a control current of 20 μA/cm^2, applied for 100 ms on a mesh that divides our square simulation domain of side 9 cm into 16 square cells of side 2.25 cm each, suffices to control spiral turbulence, even though there is a square obstacle of side 2.25 cm at (63 mm, 54 mm). (In the absence of the control pulse, a single rotating spiral wave would have been anchored to this obstacle.) The pseudocolor plots of V in Fig. 39 give (A) the initial state with an anchored spiral and its subsequent evolution after the initiation of the control pulse at (B) 100 ms, (C) 200 ms, and (D) 500 ms, after which all spiral turbulence disappears. Similar results from our simulations of the two-dimensional RPB model are given in Fig. 40; here a control current of 20 μA/cm^2, applied for 100 ms on a mesh that divides our square simulation domain of side 11.52 cm into 25 square cells of side $\simeq$ 2.25 cm each, suffices to control spiral turbulence, even though there is a square obstacle of side 2.25 cm placed at (45 mm, 36 mm). (In the absence of the control pulse, a single rotating spiral wave would have been anchored to this obstacle.) The pseudocolor plots of V in Fig. 40 give (A) the initial spiral-turbulent state and its subsequent evolution after the initiation of the control pulse at (B) 50 ms, (C) 200 ms, and (D) 400 ms, after which all spiral turbulence disappears. We also present results from our simulations of the two-dimensional TNNP model in Fig. 41; here a control current of 27.75 μA/cm^2, applied for 20 ms on a mesh that divides our square simulation domain of side 13.5 cm into 16 square cells of side 3.375 cm each, suffices to control spiral wave, even though there is a square obstacle of side 2.25 cm placed at (45 mm, 31.5 mm) in the simulation domain. (In the absence of the control pulse, a single rotating spiral wave would have been anchored to this obstacle.) The pseudocolor plots of V in Figs. 41 give (A) the initial state and its subsequent evolution after the initiation of the control pulse at (B) 24 ms, (C) 80 ms, and (D) 280 ms, after which all spiral turbulence disappears.

We have shown above how the control scheme of Ref. [9] can be extended to three-dimensions in an $L \times L \times L_z$ simulation domain, if $L_z < 2$ mm. This scheme works even in the presence of an obstacle as is illustrated for representative cases in Fig. 42 with an obstacle of size $25 \times 25 \times 2$ placed at (70 mm, 60 mm); in the absence of control pulses a single scroll wave is anchored to the obstacle. We now apply a control pulse of strength 0.48 on a mesh with square cells, each of side 16 mm, on the bottom face of the simulation domain for 748 ms; the evolution of the states of the system after the initiation of the control are depicted at (B) 220 ms, (C) 440 ms, and (D) 880 ms in both Fig. 42. By 880 ms the scroll waves have left the simulation domain and it is completely quiescent.

When $L_z > 2$ mm, the control scheme described in the previous paragraph fails just as its counterpart did in the absence of an inhomogeneity. As in the homogeneous case we have tried to use a control mesh on one face of the simulation domain, but, instead of using a single long pulse, we have used a series of short pulses, each of duration τ_w and separated by an interval τ_{ip}, to control scroll-wave turbulence in the presence of an obstacle. For the three-dimensional Panfilov model we have, in particular, tried this control scheme in a $128 \times 128 \times 4$ mm^3 simulation domain with an obstacle of size $25 \times 25 \times 4$ mm^3 placed such that on the bottom face one of its corners is at (70 mm, 60 mm); in the absence of control pulses this yields spiral turbulence. We now use a control mesh of square cells, each of side 16 mm, on the bottom face of the domain, set $\tau_w = 2$ iterations (i.e., 0.22 ms), $\tau_{ip} = 200$ iterations (22 ms), and employ a series of 32 pulses each with strength 0.48. We see from the isosurfaces of V in Fig. 43 that this turbulence persists even when

we apply the sequence of control pulses that eliminated it in the absence of the obstacle. Similarly, Fig. 44 illustrates the inadequacy of this control scheme with the same obstacle placed such that on the bottom face one of its corners is at (60 mm, 60 mm); in the absence of control this leads to a scroll wave anchored at the obstacle; this scroll wave persists even when we apply the control pulses.

References

[1] Hodgkin AL, Huxley AF (1952) A quatitative description of membrane current and its application to conduction and excitation in nerve. J Physiol 117:500.

[2] Luo C, Rudy Y (1991) A model of the ventricular cardiac action potential. Circ Res 68:1501.

[3] Qu Z, Garfinkel A (1999) An advanced algorithm for solving pde in cardiac conduction. IEEE Trans Biomed Engg 46:1166.

[4] Bernus O, Wilders R, Zemlin CW, Versschelde H, Panfilov AV (2002) A computationally efficient electrophysiological model of human ventricular cells. Am J Physiol Heart Circ Physiol 282:H2296.

[5] Shajahan TK, Sinha S, Pandit R (2007) Spiral-wave dynamics depends sensitively on inhomogeneities in mathematical models of ventricular tissue. Phys Rev E 75:011929.

[6] Pandit R, Pande A, Sinha S, Sen A (2002) Spiral turbulence and spatiotemporal chaos: Characterization and control in two excitable media. Physica A 306:211.

[7] Pande A, Sinha S, Pandit R (1999) Spiral turbulence: From the oxidation of co on pt(110) to ventricular fibrillation. Journal of Indian Institute of Science 79:31.

[8] Xie F, Qu Z, Garfinkel A, Weiss JN (2001) Electrophysiological heterogeneity and stability of reentry in simulated cardiac tissue. Am J Physiol Heart Circ Physiol 280:H535.

[9] Sinha S, Pande A, Pandit R (2001) Defibrillation via the elimination of spiral turbulence in model for ventricular fibrillation. Phys Rev Lett 86:3678.
